# Synthesis and Antimicrobial Activity of 4-Substituted 1,2,3-Triazole-Coumarin Derivatives

**DOI:** 10.3390/molecules23010199

**Published:** 2018-01-18

**Authors:** Priscila López-Rojas, Monika Janeczko, Konrad Kubiński, Ángel Amesty, Maciej Masłyk, Ana Estévez-Braun

**Affiliations:** 1 Instituto Universitario de Bio-Orgánica Antonio González (CIBICAN), Departamento de Química Orgánica, Universidad de La Laguna, Avda. Astrofísico Fco. Sánchez 2, 38206 La Laguna, Tenerife, Spain; priscila.quim@gmail.com; 2 Department of Molecular Biology, The John Paul II Catholic University of Lublin, ul. Konstantynów 1i, 20-708 Lublin, Poland; mjanec@kul.pl (M.J.); kubin@kul.pl (K.K.)

**Keywords:** triazoles, coumarin, antibacterial activity, *Enterococcus faecalis*, biofilm

## Abstract

A new series of coumarin-1,2,3-triazole conjugates with varied alkyl, phenyl and heterocycle moieties at C-4 of the triazole nucleus were synthesized using a copper(I)-catalysed Huisgen 1,3-dipolar cycloaddition reaction of corresponding *O*-propargylated coumarin (**3**) or *N*-propargylated coumarin (**6**) with alkyl or aryl azides. Based on their minimal inhibitory concentrations (MICs) against selected microorganisms, six out of twenty-six compounds showed significant antibacterial activity towards *Enterococcus faecalis* (MIC = 12.5–50 µg/mL). Moreover, the synthesized triazoles show relatively low toxicity against human erythrocytes.

## 1. Introduction

Antimicrobial resistance has been listed by the World Health Organization (WHO) as one of the biggest threats to global health today [1]. The antibiotic resistance crisis has been attributed to the overuse and misuse of these medications, as well as a lack of new drug development by the pharmaceutical industry due to reduced economic incentives and challenging regulatory requirements [2,3,4,5,6]. Over the past decade, it has become apparent that several highly resistant bacterial pathogens have acquired clever mechanisms to negate the effectiveness of numerous therapeutic agents [7]. *Staphylococcus aureus* is one bacterial pathogen that has emerged as a significant concern to healthcare professionals worldwide. In this sense, isolated strains of *S. aureus* have exhibited resistance to several classes of antibacterial drugs, including β-lactam antibiotics [8], macrolides [9], fluoroquinolones [10,11,12], glycopeptides [13] and oxazolidinones [14]. *Enterococci* were previously considered commensal organisms of little clinical importance but have emerged as serious nosocomial pathogens responsible for e.g. endocarditis and infections of the urinary tract, bloodstream, meninges, wounds and the biliary tract [15]. Recent surveillance data indicate that *Enterococcus* is the third most commonly isolated nosocomial pathogen (12% of all hospital infections), only behind coagulase-negative *Staphylococcus* and *Staphylococcus aureus* [16]. The clinical importance of the genus *Enterococcus* is directly related to its antibiotic resistance, which contributes to the risk of colonization and infection. *Enterococci* are intrinsically resistant to many commonly used antimicrobial agents (penicillins, ampicillins, cephalosporins, clindamycin) and exhibit native resistance to clinically achievable concentrations of aminoglycosides. Although *E. faecalis* is naturally resistant to quinupristin-dalfopristin, this combination is highly active against *E. faecium* strains that lack specific resistance determinants. *Enterococci* are tolerant to the (normally) bactericidal activity of cell-wall active agents, such as β-lactam antibiotics and vancomycin. Tolerance implies that the bacteria can be inhibited by clinically achievable concentrations of the antibiotic but will only be killed by concentrations far in excess of the inhibitory concentration [17]. The emergence of multi-resistant *E. faecalis* strains, complicating the treatment, means that it is important to search for and identify new treatment strategies.

All the information mentioned above highlights the urgent need to develop novel antibacterial agents devoid of cross-resistance to marketed antibiotics.

The use of privileged structures in drug discovery has proven to be an effective strategy allowing the generation of innovative hits⁄leads and successful optimization processes [18,19]. Coumarins are considered to be privileged structures due to their broad range of biological properties including anticoagulant [20], anti-neurodegenerative [21], antioxidant [22], anticancer [23] and antimicrobial activities [24,25,26,27,28]. These interesting properties of coumarins can be ascribed to the chemical attributes of the 2*H*-chromen-2-one core; its aromatic ring can establish a series of hydrophobic, π–π, CH–π and cation–π interactions and the two oxygen atoms in the lactone ring can hydrogen-bond to a series of amino acid residues in different classes of enzymes and receptors. Additionally, the double bond in the lactone helps to make the planar system, allows charge delocalization between the carbonyl group of the lactone and the aromatic ring and confers the characteristic fluorescence of this class of compounds.

On the other hand, 1,2,3-triazoles are nitrogen heterocycles capable of forming hydrogen bonds, which improves their solubility and ability to interact with biomolecular targets [29]. The 1,2,3-triazoles are highly stable to metabolic degradation, compared to other compounds containing three adjacent nitrogen (N) atoms [29]. The triazoles have been used for broad therapeutic applications due to their diverse biological activities [30], i.e. antimicrobial [31,32,33], antiviral [34], anti-inflammatory [35], analgesic [35], anticancer [36,37,38], antifungal [39] and anticonvulsant [40] activities.

Taking into consideration the antimicrobial activity shown by some coumarins and 1,2,3-triazols mentioned above and as a continuation of our project on searching for new antibacterial molecules [41,42,43,44,45], we envisaged that the linkage of coumarin and 1,2,3-triazole pharmacophores through –OCH_2_– or –NCH_2_– linkers would generate novel hybrid molecules with promising antibacterial activities.

Therefore, we herein report the synthesis and antibacterial activity of coumarin-1,2,3-triazole conjugates with varied alkyl, phenyl and heterocycle moieties at C-4 of the triazole nucleus in order to evaluate their contribution to the antimicrobial activity.

## 2. Results and Discussion

### 2.1. Chemistry

**Scheme 1:**
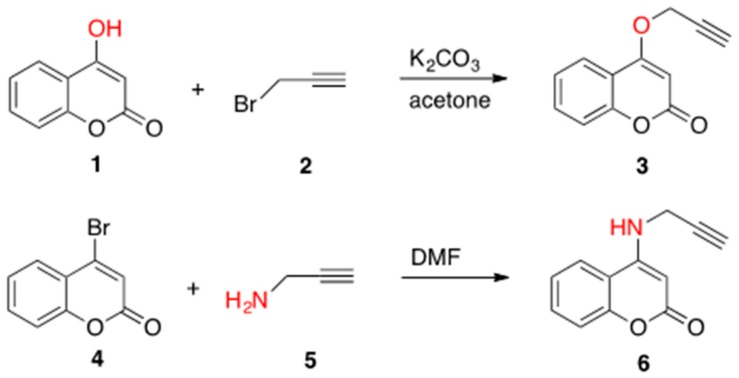
Formation of *O*-propargylated coumarin (**3**) and *N*-propargylated coumarin (**6**).

The required acetylenic dipolarophiles **3** and **6** were obtained as shown in Scheme 1. Thus, the treatment of 4-hydroxy-coumarin (**1**) with 3-bromoprop-1-yne (**2**) employing potassium carbonate in anhydrous acetone yielded the *O*-propargylated coumarin (**3**) in 55% yield. The *N*-propargylated coumarin (**6**) was obtained in 63% yield from 4-bromo-coumarin (**4**) through nucleophilic substitution with prop-2-yn-1-amine (**5**) in dimethylformamide (DMF).

**Scheme 2:**
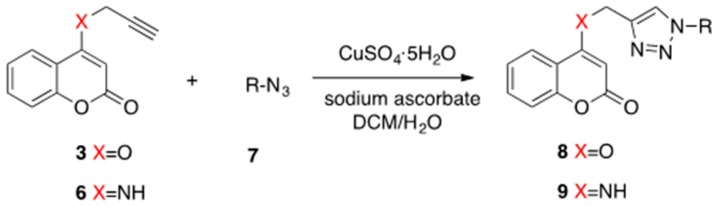
Formation of 4-substituted 1,2,3-triazole-coumarin derivatives **8** and **9**.

The 4-substituted 1,2,3-triazole-coumarin derivatives were synthesized using a copper(I)-catalysed Huisgen 1,3-dipolar cycloaddition reaction [46] of the corresponding *O*-propargylated coumarin (**3**) or *N*-propargylated coumarin (**6**) with alkyl or aryl azides (Scheme 2).

**Scheme 3:**
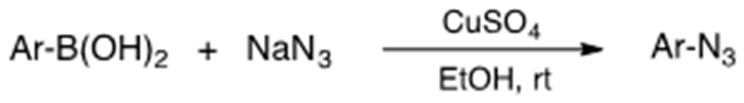
Preparation of azides **7a**–**7j**.

Azides **7a**–**7j** were prepared from the corresponding boronic acids and sodium azide, in the presence of CuSO_4_ in ethanol (EtOH), at room temperature (Table 1 and Scheme 3) [47,48].

**Scheme 4:**
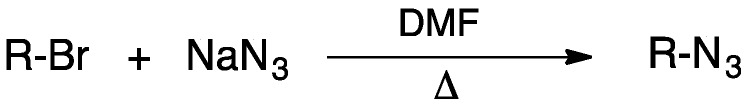
Preparation of azides **7k**–**7m**.

Azides **7k**–**7m** were obtained from the corresponding alkyl bromide or aryl bromide and sodium azide in DMF (Table 2 and Scheme 4) [49,50].

**Figure 1:**
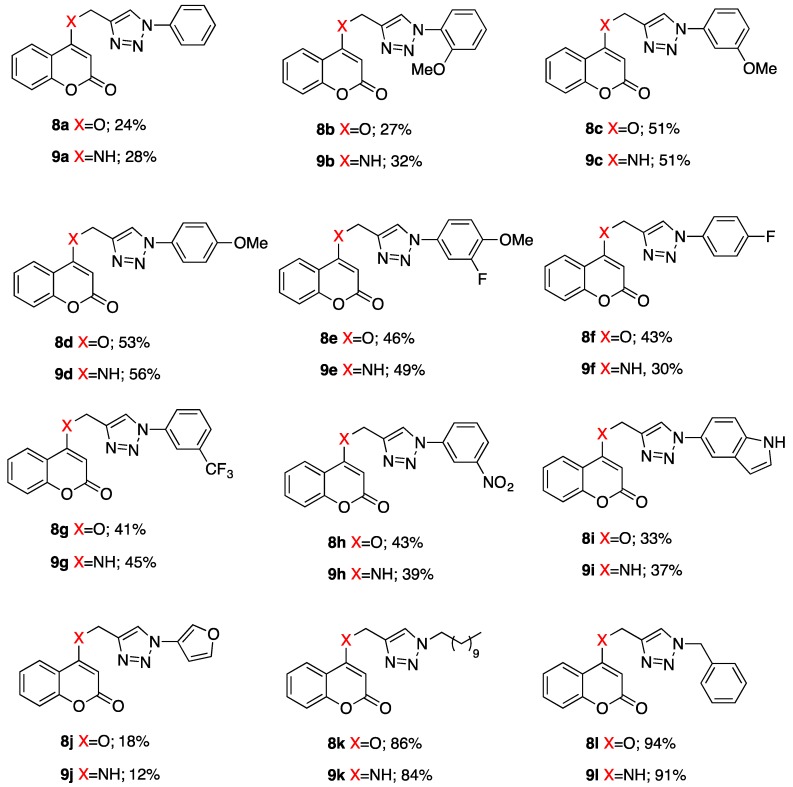

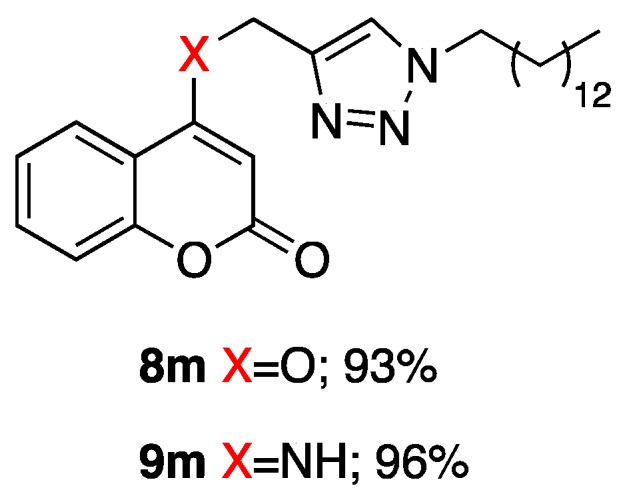
Structures of 4-substituted 1,2,3-triazole-coumarin derivatives (**8a**–**8n**) and (**9a**–**9n**).

Following the reaction shown in Scheme 2 and using azides **7a**–**7m**, compounds **8a**–**8m** and **9a**–**9m** were obtained as illustrated in Figure 1.

As can be seen, two isosteric series of coumarin derivatives were obtained (X=O, X=NH). Each series presents different substituents at the triazole moiety in order to evaluate their influence on the antimicrobial activity. Thus, coumarin derivatives with an aromatic ring having electron-donating groups or electron-withdrawing groups were prepared (**8a**–**8h**, **9a**–**9h**). Coumarin-triazole derivatives with alkyl moieties (**8k**, **9k**, **8l**, **9l**) and coumarin-indole hybrids (**8i**, **9i**) were synthesized as well. Moderate yields were obtained with aromatic azides while the use of the more stable aliphatic azides (**7k**, **7l** and **7m**) led to high yields, in agreement with the more favourable HOMO of the dipole in the 1,3-dipolar cycloaddition. The structures of all adducts were determined by spectroscopic studies. All of them showed the characteristic proton of the triazol ring in the ^1^H-NMR spectral region between δ 7.63 and 9.31. The hydrogen of the coumarin nucleus was detected as a singlet at δ 5.18–6.22 and the methylene hydrogens in the oxygenated series appeared as a singlet at δ 5.35–5.59 and as a doublet at δ 4.66–4.46 (*J* = 5.6 Hz) in the nitrogenated series.

The best yields were obtained from the *N*-propargylated coumarin (**6**) and from the aliphatic azides (**7k**, **7l** and **7m**).

### 2.2. Biology

Since some coumarins and 1,2,3-triazoles have shown potential as antibacterial drugs [41,42,43,44,45,51], these combined pharmacophores could offer some advantages e.g. in overcoming drug resistance as well as improving their biological potency.

The in vitro antimicrobial activity of the novel coumarin-1,2,3-triazole conjugates was tested against the yeast *Candida albicans,* Gram-positive bacteria *Staphylococcus aureus* and *Enterococcus faecalis* and Gram-negative bacteria *Escherichia coli*, *Klebsiella pneumonia* and *Pseudomonas aeruginosa*. The minimum inhibitory concentrations (MICs) were determined and given in Table 3. As can be seen, most of the coumarin-triazole hybrids did not exhibit considerable activity against the tested microorganisms. The best results were obtained with conjugates **8a**, **8b**, **8f**, **9h** and **9k**, which displayed promising activity against *Enterococcus faecalis* at MICs ranging from 12.5 to 50.0 µg/mL. Compound **8b** having a 2-OMe–Ph group attached at the triazol nucleus and an –OCH_2_– linker was the best of the series, while the corresponding isoster **9b** (–NHCH_2_–) turned out to be 64-fold less active than **8b**. The position of the OMe group in the phenyl ring also plays an important role in the activity, since compounds **8c** (3-OMe–Ph) and **8d** (4-OMe–Ph) showed an 8- and 16-fold lower antibacterial activity, respectively, than **8b**. In the nitrogenated series, compounds **9h** (3-NO_2_–Ph) and **9k** having an undecyl chain showed the best activities.

In other studies, menthyl 1,4-disubstituted 1,2,3-triazole derivatives of hydroxybenzaldehydes, phenols and bile acids showed a strong inhibitory effect against *E. faecium* with the minimum inhibitory concentration (MIC) values in the range of 1–3 μM [52]. Kant and co-workers reported that 1,2,3-triazole linked chalcone and flavone hybrids showed activity against Gram-positive bacteria (*Staphylococcus aureus*, *Enterococcus faecalis*) and Gram-negative bacteria (*Escherichia coli*, *Pseudomonas aeruginosa*, *Shigella boydii*, *Klebsiella pneumoniae*) with MIC values in the range of 6.25–100 µg/mL [53]. In turn, 1,2,4-triazolo[3,4a]phthalazine derivatives showed inhibitory activity against *Staphylococcus aureus* (MIC 16–128 µg/mL) [54].

**Figure 2:**
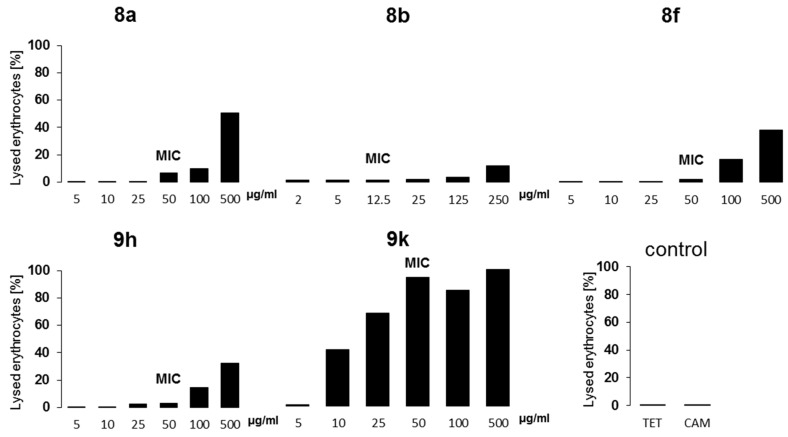
Haemolytic activity of compounds **8a**, **8b**, **8f**, **9h** and **9k**. Tetracycline (TET) and chloramphenicol (CAM) in MIC concentrations were used as a control.

In order to verify if the newly synthesized triazoles could be considered as potential antimicrobial therapeutics, the most active compounds, namely **8a**, **8b**, **8f**, **9h** and **9k** were examined in terms of their haemolytic activity against human erythrocytes. The results are shown in Figure 2.

Compounds **8b**, **8f** and **9h** exhibit minimal toxicity towards human blood cells (1.6–3.1% of lysed cells) in MIC. Although compound **8b** appears to be the most active antimicrobial agent, simultaneously it moderately affects the erythrocytes (6.9% of lysed cells) in MIC. The presence of an undecyl chain in the triazole ring (**9k**) results in a drastic increase in the haemolytic activity (94% of lysed cells) in MIC.

## 3. Materials and Methods

### 3.1. Compounds Synthesis

#### 3.1.1. General Experimental Procedures

IR spectra were obtained using a Fourier Transform Infrared spectrometer. NMR spectra were recorded in CDCl_3_ or DMSO at 500 or 600 MHz for ^1^H NMR and 125 or 150 MHz for ^13^C-NMR. Chemical shifts are given in (δ) parts per million and coupling constants (*J*) in hertz (Hz). ^1^H- and ^13^C-spectra were referenced using the solvent signal as an internal standard. Melting points were taken on a capillary melting point apparatus and are uncorrected. Microwave reactions were conducted in sealed glass vessels (capacity 5 mL) using a CEM Discover microwave reactor. HREIMS were recorded using a high-resolution magnetic trisector (EBE) mass analyser. The analytical thin-layer chromatography plates used were Polygram-Sil G/UV254. Preparative thin-layer chromatography was carried out with Analtech (Newark, NJ, USA) silica gel GF plates (20 × 20 cm, 1000 Microns) using appropriate mixtures of ethyl acetate and hexanes. All solvents and reagents were purified by standard techniques reported in [55] or used as supplied from commercial sources. All compounds were named using the ACD40 Name-Pro program, which is based on IUPAC rules. Azides **7a**–**7m** were synthesized according to procedures previously described in the literature [47,48,49,50,56].

*4-(Prop-2-yn-1-yloxy)-2H-chromen-2-one* (**3**). 259 µL (2.4 mmol) of propargyl bromide were slowly added to a mixture of 330.9 mg (2.0 mmol) of 4-hydroxycoumarin and 552.8 mg (4.0 mmol) of K_2_CO_3_ in 15 mL of acetone. The reaction mixture was refluxed for 8 h until disappearance of the starting coumarin. Then, the solvent was eliminated under reduced pressure, 30 mL of H_2_O were added and the mixture was extracted with AcOEt (3 × 30 mL). The organic phases were collected, washed with H_2_O (20 mL) and brine (20 mL) and dried over anhydrous MgSO_4_. After filtration and elimination of the solvent, the crude extract was purified by silica gel column chromatography using DCM as an eluent and 224.7mg (55%) of compound **3** were obtained as an amorphous white solid. Compound **3** showed identical spectroscopic data to those described in the literature [57].

*4-(Prop-2-yn-1-ylamino)-2H-chromen-2-one* (**6**). 47 µL (0.72 mmol) of prop-2-yn-1-amine were slowly added to 200 mg (0.89 mmol) of 4-bromocoumarin in 2 mL of dimethylformamide (DMF) under argon atmosphere. The reaction mixture was stirred at room temperature for 18 h. Then water was added and the *N*-propargylated coumarin precipitated. After filtration, 111.8 mg (63%) of compound (**6**) was obtained as an amorphous white solid. m.p. 223–224 °C; ^1^H-NMR (600 MHz, (CDCl_3_) δ 7.57 (1H, t, *J* = 8.1 Hz), 7.46 (1H, d, *J* = 8.1 Hz), 7.37 (1H, d, *J* = 8.1 Hz), 7.30 (1H, t, *J* = 8.1 Hz), 5.46 (1H, s), 5.28 (1H, bs), 4.11 (2H, dd, *J* = 5.5, 2.5 Hz), 2.41 (1H, t, *J* = 2.5 Hz); ^13^C-NMR (150 MHz, (CDCl_3_) δ 162.5 (C=O), 153.6 (C), 151.8 (C), 132.0 (CH), 123.6 (CH), 119.9 (CH), 118.1 (CH), 113.9 (C), 85.9 (CH), 77.6 (C), 73.6 (CH_2_), 32.9(CH) ppm; EIMS *m*/*z* 199 ([M^+^], 71); 198 (61); 197 (13); 171 (100); 170 (53); 144 (13); 143 (22); 142 (27); 119 (12); 118 (16); 115 (15); 90 (11); 77 (18); 63 (14); 51 (14); HREIMS 199.0638 (calcd. for C_12_H_9_NO_2_ [M^+^] 199.0633); FT-IR (ATR) ν_max_ 3325, 3259, 3093, 3074, 2934, 2122, 1807, 1668, 1612, 1552, 1484, 1445, 1389, 1354, 1328, 1271, 1192, 1147, 1045, 983, 937, 865, 811 cm^−1^.

#### 3.1.2. General Procedures for the Preparation of 4-Substituted 1,2,3-Triazole-Coumarin Derivatives

*Method A.* Corresponding boronic acid (0.24 mmol) and 78.5 mg (1.2 mmol) of sodium azide in 1.5 mL of H_2_O were added to a vigorously stirred mixture of 3.4 mg (0.0241 mmol) of Cu_2_O in 0.06 mL of 20% of NH_3_ and 0.12 mL of H_2_O. The reaction mixture was stirred for 16 h at room temperature under an oxygen atmosphere. Then, 0.14 mmol of propargylated coumarin (**3** or **6**), 8.11 mg (0.041 mmol) of sodium ascorbate, 1.5 mL of H_2_O and 3 mL of acetone were added. The reaction was left at room temperature for 48h. Then, the reaction mixture was extracted with EtOAc. The aqueous phase was acidified with 5% HCl until pH = 2 and extracted with EtOAc (3 × 15 mL). The organic phases were collected, dried over anhydrous MgSO_4_ and after elimination of the solvent, the corresponding residue was purified by silica gel CC or TLC-preparative with DCM or 5% DCM/MeOH.

*Method B.* To a solution of 0.28 mmol of the corresponding azide in 3 mL of DCM, 0.14 mmol of propargylated coumarin (**3** or **6**), 3.6 mg (0.02 mmol) of sodium ascorbate, 1.2 mg (0.004 mmol) of CuSO_4_·5H_2_O and 3 mL of H_2_O, were added. The reaction mixture was stirred for 48 h at room temperature. The reaction mixture was extracted with EtOAc (3 × 15 mL). The organic phases were collected, dried over anhydrous MgSO_4_ and after elimination of the solvent, the corresponding residue was purified by silica gel CC or TLC-preparative with DCM or 5% DCM/MeOH.

*4-((1-Phenyl-1H-1,2,3-triazol-4-yl)methoxy)-2H-chromen-2-one* (**8a**). Following the experimental procedure described in method A, from 31.2 mg (0.24 mmol) of phenyl boronic acid and 28.0 mg (0.14 mmol) of *O*-propargylated coumarin (**3**), 10.9 mg (24%) of compound **8a** were obtained as an amorphous orange solid [58]. m.p. 193–194 °C; ^1^H-NMR (500 MHz, (CD_3_)_2_SO) δ 9.09 (1H,s), 7.95 (2H, d, *J* = 7.5 Hz), 7.83 (1H, dd, *J* = 7.9, 1.5 Hz), 7.65–7.63 (3H, m), 7.52 (1H, t, *J* = 7.4 Hz), 7.41 (1H, dd, *J* = 8.3, 0.7 Hz), 7.34 (1H, t, *J* = 7.6 Hz), 6.21 (1H, s), 5.53 (2H, s) ppm; ^13^C-NMR (125 MHz, (CD_3_)_2_SO) δ 164.4 (C=O), 161.6 (C), 152.8 (C), 142.3 (C), 136.5 (C), 132.9 (CH), 129.9 (2CH), 128.9 (CH), 124.2 (CH), 123.4 (CH), 123.1 (CH), 120.3 (2CH), 116.5 (CH), 115.1 (C), 91.5 (CH), 62.8 (CH_2_) ppm; EIMS *m*/*z* 319 ([M^+^], 26); 131 (11); 130 (100); 103 (11); 77 (47); 51 (12); HREIMS319.0967 (calcd. for C_18_H_13_N_3_O_3_ [M^+^] 319.0957); FT-IR (ATR) ν_max_ 3386, 3146, 3097, 1716, 1621, 1563, 1494, 1460, 1397, 1367, 1337, 1243, 1208, 1186, 1136, 1100, 1029, 925, 835 cm^−1^.

*4-((1-(2-Methoxyphenyl)-1H-1,2,3-triazol-4-yl)methoxy)-2H-chromen-2-one* (**8b**). Following the experimental procedure described in method B, from 42.3 mg (0.28 mmol) of 1-azido-2-methoxybenzene and 28 mg (0.14 mmol) of *O*-propargylated coumarin (**3**), 14.2 mg (27%) of compound **8b** were obtained as an amorphous white solid. m.p. 200–201 °C; ^1^H-NMR (500 MHz, CDCl_3_) δ 8.33 (1H, s), 7.84 (2H, dd, *J* = 7.9, 1.3 Hz), 7.56 (1H, t, *J* = 8.5 Hz), 7.47 (1H, t, *J* = 8.5 Hz), 7.32 (1H, dd, *J* = 8.3, 0.6 Hz), 7.25 (1H, td, *J* = 7.8, 1.1 Hz), 7.16–7.11 (2H, m), 5.93 (1H, s), 5.43 (2H, s), 3.93 (3H, s) ppm; ^13^C-NMR (125 MHz, CDCl_3_) δ 165.2 (C=O), 162.8 (C), 153.4 (C), 151.1 (C), 140.8 (C), 132.6 (CH), 130.6 (CH), 126.0 (C), 125.9 (CH), 125.5 (CH), 124.0 (CH), 123.4 (CH), 121.4 (CH), 116.8 (CH), 115.6 (C), 112.4 (CH), 91.2 (CH), 62.8 (CH_2_), 56.2 (CH_3_) ppm; EIMS *m*/*z* 349 ([M^+^], 37); 161 (12); 160 (100); 145 (28); 120 (14); 92 (11); 77 (16); HREIMS 349.1050 (calcd. for C_19_H_15_N_3_O_4_ [M^+^] 349.1063); FT-IR (ATR) ν_max_ 3400, 3129, 3093, 3009, 2942, 2841, 2287, 1707, 1617, 1563, 1507, 1493, 1477, 1460, 1410, 1368, 1233, 1186, 1136, 1103, 1052, 1018, 970, 930, 883, 868, 811 cm^−1^.

*4-((1-(3-Methoxyphenyl)-1H-1,2,3-triazol-4-yl)methoxy)-2H-chromen-2-one* (**8c**). Following the experimental procedure described in method B, from 42.3 mg (0.28 mmol) of 1-azido-2-methoxybenzene and 28 mg (0.14 mmol) of *O*-propargylated coumarin (**3**), 26.8 mg (51%) of compound **8c** were obtained as an amorphous white solid. m.p. 188–189 °C; ^1^H-NMR (500 MHz, CDCl_3_) δ 8.16 (1H, s), 7.82 (1H, d, *J* = 7.6 Hz), 7.56 (1H, t, *J* = 7.4 Hz), 7.45 (1H, t, *J* = 8.1 Hz), 7.37 (1H, s), 7.33 (1H, d, *J* = 8.1 Hz), 7.30–7.21 (2H, m), 7.00 (1H, d, *J* = 7.8 Hz), 5.91 (1H, s), 5.43 (2H, s), 3.90 (3H, s) ppm;^13^C-NMR (125 MHz, CDCl_3_) δ 165.1 (C=O), 162.7 (C), 160.8 (C), 153.5 (C), 142.2 (C), 137.9 (C), 132.7 (CH), 130.8 (CH), 124.1 (CH), 123.3 (CH), 121.9 (CH), 116.9 (CH), 115.6 (C), 115.2 (CH), 112.7 (CH), 106.7 (CH), 91.4 (CH), 62.7 (CH_2_), 55.8 (CH_3_) ppm; EIMS *m*/*z* 349 ([M^+^], 49); 160 (100); 145 (18); 130 (16); 117 (12); 107 (14); 92 (18); 77 (21); HREIMS 349.1078 (calcd. for C_19_H_15_N_3_O_4_ [M^+^] 349.1063); FT-IR (ATR) ν_max_ 3401, 3148, 3093, 2933, 2838, 1719, 1620, 1610, 1565, 1493, 1456, 1400, 1368, 1337, 1237, 1189, 1158, 1103, 1044, 1004, 931, 833 cm^−1^.

*4-((1-(4-Methoxyphenyl)-1H-1,2,3-triazol-4-yl)methoxy)-2H-chromen-2-one* (**8d**). Following the experimental procedure described in method B, from 42.3 mg (0.28 mmol) of 1-azido-4-methoxybenzene and 28.0 mg (0.14 mmol) of *O*-propargylated coumarin (**3**), 27.7 mg (53%) of compound **8d** were obtained as an amorphous white solid. m.p. 195–196 °C; ^1^H-NMR (500 MHz, CDCl_3_) δ 8.07 (1H, s), 7.83 (1H, d, *J* = 8.0 Hz), 7.66 (2H, d, *J* = 8.9 Hz), 7.56 (1H, t, *J* = 7.8 Hz), 7.33 (1H, d, *J* = 8.3 Hz), 7.4 (1H, t, *J* = 7.9 Hz), 7.05 (2H, d, *J* = 8.9 Hz), 5.91 (1H, s), 5.42 (2H, s), 3.89 (3H, s) ppm; ^13^C-NMR (125 MHz, CDCl_3_) δ 165.1 (C=O), 162.7 (C), 160.3 (C), 153.5 (C), 142.1 (C), 132.7 (CH), 130.3 (C), 124.1 (CH), 123.3 (CH), 122.5 (2CH), 121.9 (CH), 116.9 (CH), 115.6 (C), 115.0 (2CH), 91.4 (CH), 62.8 (CH_2_), 55.8 (CH_3_) ppm; EIMS *m*/*z* 349 ([M^+^], 29); 161 (12); 160 (100); 145 (13); 92 (12); 77 (13); HREIMS 349.1061 (calcd. for C_19_H_15_N_3_O_4_ [M^+^] 349.1063); FT-IR (ATR) ν_max_ 3140, 3095, 3006, 2942, 2840, 2381, 2055, 1719, 1619, 1564, 1519, 1457, 1400, 1369, 1257, 1240, 1185, 1137, 1102, 1030, 927, 824 cm^−1^.

*4-((1-(3-Fluoro-4-methoxyphenyl)-1H-1,2,3-triazol-4-yl)methoxy)-2H-chromen-2-one* (**8e**). Following the experimental procedure described in method B, from 47.4 mg (0.28 mmol) of 1-azido-3-fluoro-4-methoxybenzene and 28 mg (0.14 mmol) of *O*-propargylated coumarin (**3**), 25.3 mg (46%) of compound **8e** were obtained as an amorphous white solid. m.p. 190–191 °C; ^1^H-NMR (600 MHz, CDCl_3_) δ 8.07 (1H, s), 7.82 (1H, dd, *J* = 8.0, 1.0 Hz), 7.60–7.51 (2H, m), 7.48 (1H, d, *J* = 8.8 Hz), 7.33 (1H, d, *J* = 8.3 Hz), 7.24 (1H, t, *J* = 7.9 Hz), 7.11 (1H, t, *J* = 8.7 Hz), 5.91 (1H, s), 5.42 (2H, s), 3.97 (3H, s) ppm; ^13^C-NMR (150 MHz, CDCl_3_) δ 165.1 (C=O), 162.7 (C), 153.4 (C), 152.3 (*J*^1^_C–F_ = 249.1 Hz), 148.6 (C, *J*^2^_C–F_ = 28.7 Hz), 142.4 (C), 132.8 (CH), 124.1 (CH), 123.3 (CH), 121.8 (CH), 117.0 (CH), 116.8 (CH, *J*^3^_C–F_ = 3.6 Hz), 115.6 (C), 114.0 (CH, *J*^3^_C–F_ = 1.9 Hz), 110.0 (CH, *J*^2^_C–F_ = 22.6 Hz), 91.5 (CH), 62.7 (CH_2_), 56.7 (CH_3_) ppm; EIMS *m*/*z* 367 ([M^+^], 27); 179 (12); 178 (100); 163 (21); HREIMS367.0898 (calcd. for C_19_H_14_N_3_O_4_F [M^+^] 367.0968); FT-IR (ATR) ν_max_ 3488, 3143, 3089, 2976, 2944, 2844, 2361, 1715, 1620, 1565, 1527, 1493, 1451, 1401, 1368, 1328, 1287, 1243, 1233, 1186, 1159, 1133, 1104, 1017, 952, 933, 880, 833, 808 cm^−1^.

*4-((1-(4-Fluoro-phenyl)-1H-1,2,3-triazol-4-yl)methoxy)-2H-chromen-2-one* (**8f**). Following the experimental procedure described in method A, from 34.8 mg (0.24 mmol) of 4-fluorophenyl boronic acid and 28 mg (0.14 mmol) of *O*-propargylated coumarin (**3**), 22.1 mg (43%) of compound **8f** were obtained as an amorphous white solid. m.p. 233–235 °C; ^1^H-NMR (500 MHz, (CD_3_)_2_SO) δ 9.07 (1H, s), 8.02–7.98 (2H, m), 7.83 (1H, dd, *J =* 7.9, 1.5 Hz), 7.67 (1H, ddd, *J =* 8.7, 7.4, 1.6 Hz), 7.49 (2H, t, *J =* 8.8 Hz), 7.42 (1H, dd, *J =* 8.3, 0.6 Hz), 7.35 (1H, t, *J =* 7.6 Hz), 6.20 (1H, s), 5.52 (2H, s) ppm; ^13^C-NMR (125 MHz, (CD_3_)_2_SO) δ 164.3 (C=O), 161.5 (C), 161.4 (C, *J*^1^_C–F_ = 245.7 Hz), 152.7 (C), 142.3 (C), 133.0 (C, *J*^4^_C–F_ = 3.4 Hz), 132.9 (CH), 124.2 (CH), 123.6 (CH), 123.0 (CH), 122.7 (2 CH, *J*^3^_C–F_ = 8.6 Hz), 116.8 (2 CH, *J*^2^_C–F_ = 24.1 Hz), 116.5 (CH), 115.1 (C), 91.5 (CH), 62.8 (CH_2_); EIMS *m*/*z* 337 ([M^+^], 23); 149 (11); 148 (100); 95 (25); HREIMS 337.0859 (calcd. for C_18_H_12_N_3_O_3_F [M^+^] 337.0863). FT-IR (ATR) ν_max_ 3148, 3098, 2384, 2294, 2050, 1718, 1624, 1567, 1516, 1496, 1465, 1454, 1401, 1370, 1233, 1184, 1105, 1051, 1022, 951, 931, 833 cm^−1^.

*4-((1-(3-Trifluoromethyl-phenyl)-1H-1,2,3-triazol-4-yl)methoxy)-2H-chromen-2-one* (**8g**). Following the experimental procedure described in method B, from 53.2 mg (0.28 mmol) of 1-azido-3-trifluoromethylbenzene and 28 mg (0.14 mmol) of *O*-propargylated coumarin (**3**), 24.0 mg (41%) of compound **8g** were obtained as an amorphous white solid. m.p. 192–193 °C; ^1^H-NMR (500 MHz, (CD_3_)_2_SO) δ 9.27 (1H, s), 8.34 (1H, s), 8.32 (1H, d, *J* = 8.2 Hz), 7.93–7.86 (2H, m), 7.85 (1H, dd, *J* = 8.0, 1.5 Hz), 7.68 (1H, ddd, *J* = 8.8, 7.4, 1.6 Hz), 7.43 (1H, d, *J* = 7.7 Hz), 7.37 (1H, t, *J* = 7.6 Hz), 6.22 (1H, s), 5.56 (2H, s) ppm; ^13^C-NMR (125 MHz, (CD_3_)_2_SO) δ 164.3 (C=O), 165.1 (C), 152.7 (C), 142.5 (C), 136.9 (C), 132.8 (CH), 131.3 (CH), 130.5 (C, *J*^2^_C–F_ = 29.6 Hz), 125.4 (CH, *J*^3^_C–F_ = 4.1 Hz), 124.2 (CH), 124.1 (CH), 123.5 (C, *J*^1^_C–F_ = 276.4 Hz), 123.7 (CH), 123.0 (CH), 116.9 (CH, *J*^3^_C–F_ = 4.5 Hz), 116.4 (CH), 115.0 (C), 91.5 (CH), 62.7 (CH_2_);EIMS *m*/*z* 387 ([M^+^], 12); 386 (42); 358 (23); 357 (45); 329 (10); 199 (12); 198 (100); 159 (26); 145 (50); HREIMS 387.0846 (calcd. for C_19_H_12_N_3_O_3_F_3_ [M^+^] 387.0831); FT-IR (ATR) ν_max_ 3531, 3409, 1685, 1618, 1559, 1069, 972 cm^–1^.

*4-((1-(3-Nitro-phenyl)-1H-1,2,3-triazol-4-yl)methoxy)-2H-chromen-2-one* (**8h**). Following the experimental procedure described in method B, from 46.6 mg (0.28 mmol) of 1-azido-3-nitrobenzene and 28 mg (0.14 mmol) of *O*-propargylated coumarin (**3**), 23.1 mg (43%) of compound **8g** were obtained as an amorphous white solid. m.p. 215–217 °C; ^1^H-NMR (600 MHz, (CD_3_)_2_SO) δ 9.31 (1H, s), 8.78 (1H, s), 8.45 (1H, d, *J* = 7.8 Hz), 8.36 (1H, d, *J* = 7.9 Hz), 7.93 (1H, t, *J* = 8.1 Hz), 7.85 (1H, d, *J* = 7.8 Hz), 7.67 (1H, t, *J* = 7.6 Hz), 7.42 (1H, d, *J* = 8.3 Hz), 7.36 (1H, t, *J* = 7.5 Hz), 6.21 (1H, s), 5.56 (2H, s) ppm; ^13^C-NMR (150 MHz, (CD_3_)_2_SO) δ 164.4 (C=O), 161.6 (C), 152.8 (C), 148.6 (C), 142.8 (C), 137.1 (C), 133.0 (CH), 131.7 (CH), 126.4 (CH), 124.3 (CH), 123.9 (CH), 123.4 (CH), 123.1 (CH), 116.5 (CH), 115.2 (CH), 115.1 (C), 91.6 (CH), 62.8 (CH_2_) ppm; EIMS *m*/*z* 364 ([M^+^], 59); 176 (11); 175 (100); 162 (23); 145 (14); 129 (92); 128 (37); 121 (14); 120 (39); 92 (18); 77 (11); 76 (37); HREIMS 364.0820 (calcd. for C_18_H_12_N_4_O_5_ [M^+^] 364.0808); FT-IR (ATR) ν_max_ 3431, 3146, 3091, 2925, 1713, 1617, 1565, 1531, 1493, 1464, 1406, 1352, 1245, 1185, 1138, 1104, 1045, 1009, 980, 952, 929, 830 cm^–1^.

*4-((1-(1H-Indole-5-yl)-1H-1,2,3-triazol-4-yl)methoxy)-2H-chromen-2-one* (**8i**). Following the experimental procedure described in method B, from 44.9 mg (0.28 mmol) of 5-azido-1*H*-indol and 28 mg (0.14 mmol) of *O*-propargylated coumarin (**3**), 18.1 mg (33%) of compound **8i** were obtained as an amorphous white solid. m.p. 191–193 °C; ^1^H-NMR (500 MHz, (CD_3_)_2_SO) δ 11.46 (1H, s), 8.99 (1H, s), 8.05 (1H, s), 7.84 (1H, d, *J* = 7.1 Hz), 7.67 (1H, t, *J* = 7.2 Hz), 7.63–7.57 (2H, m), 7.53–7.51 (1H, m), 7.42 (1H, d, *J* = 8.3 Hz), 7.35 (1H, t, *J* = 7.5 Hz), 6.59 (1H, s), 6.22 (1H, s), 5.52 (2H, s); ^13^C NMR (125 MHz, (CD_3_)_2_SO) 164.4 (C=O), 161.6 (C), 152.8 (C), 141.8 (C), 135.6 (C), 132.9 (CH), 129.3 (C), 127.8 (CH), 127.6 (C), 124.3 (CH), 123.7 (CH), 123.1 (CH), 116.5 (CH), 115.1 (C), 114.4 (CH), 112.4 (CH), 112.3 (CH), 102.0 (CH), 91.4 (CH), 62.9 (CH_2_) ppm; EIMS *m*/*z* 358 ([M^+^], 28); 169 (100); 168 (19); 162 (30); 121 (13); 120 (39); 116 (28); 92 (14); HREIMS 358.1068 (calcd. for C_20_H_14_N_4_O_3_ [M^+^] 358.1066); FT-IR (ATR) ν_max_ 3399, 3299, 1699, 1685, 1616, 1563, 1494, 1403, 1350, 1327, 1228, 1097, 1048, 1025, 993 cm^–1^.

*4-((1-(Furan-3-yl)-1H-1,2,3-triazol-4-yl)methoxy)-2H-chromen-2-one* (**8j**). Following the experimental procedure described in method A, from 34.8 mg (0.24mmol) of 3-furylboronic acid and 28 mg (0.14 mmol) of *O*-propargylated coumarin (**3**), 8.1 mg (18%) of compound **8j** were obtained as an amorphous white solid. m.p. 189–190 °C; ^1^H-NMR (500 MHz, (CD_3_)_2_SO) δ 8.88 (1H,s), 8.48 (1H, s), 7.91 (1H, t, *J* = 1.9 Hz), 7.80 (1H, dd, *J* = 7.9, 1.5 Hz), 7.67 (1H, t, *J* = 7.6Hz), 7.42 (1H, dd, *J* = 8.3, 0.7 Hz), 7.35 (1H, t, *J* = 7.8 Hz), 7.16 (1H, dd, *J* = 2.0, 0.9 Hz), 6.19 (1H, s), 5.51 (2H, s) ppm; ^13^C-NMR (125 MHz, (CD_3_)_2_SO) δ 164.4 (C=O), 161.6 (C), 152.8 (C), 144.9 (CH), 141.9 (C), 134.3 (CH), 132.9 (CH), 125.9 (C), 124.3 (CH), 124.2 (CH), 123.0 (CH), 116.5 (CH), 115.1 (C), 105.3 (CH), 91.5 (CH), 62.7 (CH_2_) ppm; EIMS *m*/*z* 309 ([M^+^], 59); 279 (25); 198 (21); 159 (23); 120 (100); 94 (18); 65 (13); HREIMS 309.0662 (calcd. for C_16_H_11_N_3_O_4_ [M^+^] 309.0671). FT-IR (ATR) ν_max_ 3514, 3399, 3084, 2924, 2387, 2094, 2064, 1992, 1696, 1619, 1606, 1563, 1492, 1450, 1410, 1365, 1270, 1248, 1193, 1105, 1053, 1023, 942, 911, 871, 839 cm^−1^.

*4-((1-Undecyl-1H-1,2,3-triazol-4-yl)methoxy)-2H-chromen-2-one* (**8k**). Following the experimental procedure described in method B, from 56.1 mg (0.28mmol) of 1-azido-undecane and 28 mg (0.14 mmol) of *O*-propargylated coumarin (**3**), 51.4 mg (86%) of compound **8k** were obtained as an amorphous white solid. m.p. 144–145 °C; ^1^H-NMR (500 MHz, CDCl_3_) δ 7.78 (1H, dd, *J* = 13.2, 6.5 Hz), 7.75 (1H, s), 7.54 (1H, t, *J* = 7.5 Hz), 7.30 (1H, d, *J* = 7.9 Hz), 7.24 (1H, t, *J* = 7.6 Hz), 5.87 (1H, s), 5.34 (1H, s), 4.41 (2H, t, *J* = 7.3 Hz), 1.96 (2H, t, *J* = 7.2 Hz), 1.38–1.23 (16H, m), 0.87 (3H, t, *J* = 6.9 Hz) ppm; ^13^C-NMR (125 MHz, CDCl_3_) δ 165.1 (C=O), 162.7 (C), 153.4 (C), 141.4 (C), 132.6 (CH), 124.0 (CH), 123.4 (CH), 123.2 (CH), 116.8 (CH), 115.5 (C), 91.2 (CH), 62.8 (CH_2_), 50.7 (CH_2_), 31.9 (CH_2_), 30.3 (CH_2_), 29.6 (CH_2_), 29.5 (CH_2_), 29.4 (CH_2_), 29.3 (CH_2_), 29.0 (CH_2_), 26.6 (CH_2_), 22.7 (CH_2_), 14.2 (CH_3_) ppm; EIMS *m*/*z* 397 ([M^+^], 18); 236 (17); 209 (15); 208 (100); 68 (21); 57 (16); 55 (15); HREIMS 397.2351 (calcd. for C_23_H_31_N_3_O_3_ [M^+^] 397.2365); FT-IR (ATR) ν_max_ 3134, 3075, 2918, 2849, 1725, 1623, 1610, 1566, 1492, 1458, 1424, 1381, 1328, 1272, 1248, 1187, 1154, 1138, 1106, 1059, 1031, 979, 931, 882, 851, 814 cm^−1^.

*4-((1-Benzyl-1H-1,2,3-triazol-4-yl)methoxy)-2H-chromen-2-one* (**8l**). Following the experimental procedure described in method B, from 40.8 mg (0.28 mmol) of benzylazide and 28 mg (0.14 mmol) of *O*-propargylated coumarin (**3**), 47.0 mg (94%) of compound **8l** were obtained as an amorphous white solid. m.p. 210–211 °C; ^1^H-NMR (500 MHz, CDCl_3_) δ 7.76 (1H, dd, *J* = 7.9, 1.5 Hz), 7.63 (1H, s), 7.56–7.52 (1H, t, *J* = 7.6 Hz), 7.47–7.36 (3H, m), 7.35–7.28 (3H, m), 7.23 (1H, t, *J* = 7.6 Hz), 5.84 (s, 1H), 5.59 (s, 2H), 5.31 (2H, d, *J* = 3.8 Hz) ppm; ^13^C-NMR (125 MHz, CDCl_3_) δ 165.1 (C=O), 162.5 (C), 153.7 (C), 142.1 (C), 134.4 (C), 132.6 (CH), 129.5 (2CH), 129.2 (CH), 128.4 (2CH), 124.0 (CH), 123.3 (CH), 123.2 (CH), 117.0 (CH), 115.8 (C), 91.5 (CH), 62.9 (CH_2_), 54.6 (CH_2_) ppm; EIMS *m*/*z* 333 ([M^+^], 39); 172 (13); 144 (62); 104 (11); 92 (14); 91 (100); HREIMS 333.1116 (calcd. for C_19_H_15_N_3_O_3_[M^+^] 333.1113); FT-IR (ATR) ν_max_ 3075, 1722, 1625, 1569, 1498, 1459, 1421, 1377, 1331, 1277, 1250, 1187, 1141, 1111, 1063, 1035, 983, 933, 885, 849, 814 cm^−1^.

*4-((1-Tetradecyl-1H-1,2,3-triazol-4-yl)methoxy)-2H-chromen-2-one* (**8m**). Following the experimental procedure described in method B, from 68.0 mg (0.28 mmol) of 1-azido-tetradecane and 28 mg (0.14 mmol) of *O*-propargylated coumarin (**3**), 61.3 mg (93%) of compound **8m** were obtained as an amorphous white solid. m.p. 146–147 °C; ^1^H-NMR (500 MHz, CDCl_3_) δ 7.79 (1H, dt, *J* = 7.9, 1.5 Hz), 7.72 (1H, s), 7.54 (1H, tt, *J* = 9.1, 1.7 Hz), 7.31 (1H, dd, *J* = 8.4, 1.5 Hz), 7.24 (1H, t, *J* = 7.6 Hz), 5.87 (1H, s), 5.35 (2H, s), 4.41 (2H, t, *J* = 7.3 Hz), 1.96 (2H, t, *J* = 7.1 Hz), 1.38–1.23 (22H, m), 0.88 (3H, t, *J* = 6.9 Hz) ppm; ^13^C-NMR (125 MHz, CDCl_3_) δ 165.1 (C=O), 162.8 (C), 153.4 (C), 141.4 (C), 132.6 (CH), 124.0 (CH), 123.4 (CH), 123.3 (CH), 116.8 (CH), 115.6 (C), 91.2 (CH), 62.8 (CH_2_), 50.8 (CH_2_), 32.0 (CH_2_), 31.0 (CH), 30.4 (CH_2_), 29.9 (CH_2_), 29.8 (CH_2_), 29.7 (2CH_2_), 29.6 (CH_2_), 29.5 (CH_2_), 29.4 (CH_2_), 29.1 (CH_2_), 26.6 (CH_2_), 22.8 (CH_2_), 14.2 (CH_3_) ppm; EIMS *m*/*z* 439 ([M^+^], 11); 278 (26); 251 (19); 250 (100); 215 (20); 120 (10); 71 (14); 70 (12); 68 (23); 57 (22); 56 (10); 55 (19); HREIMS 439.2852 (calcd. for C_26_H_37_N_3_O_3_ [M^+^] 439.2835); FT-IR (ATR) ν_max_ 3135, 3075, 2918, 2849, 1817, 1726, 1624, 1567, 1468, 1423, 1381, 1328, 1273, 1248, 1185, 1154, 1139, 1107, 1058, 979, 931, 882, 851, 814 cm^−1^.

*4-(((1-Phenyl-1H-1,2,3-triazol-4-yl)methyl)amino)-2H-chromen-2-one* (**9a**). Following the experimental procedure described in method A, from 31.2 mg (0.24 mmol) of phenyl boronic acid and 28 mg (0.14 mmol) of *N*-propargylated coumarin (**6**), 13.8 mg (28%) of compound **9a** were obtained as an amorphous white solid. m.p. 194–195 °C; ^1^H-NMR (500 MHz, (CD_3_)_2_SO) δ 8.82 (1H, s), 8.31 (1H, t, *J* = 5.6 Hz), 8.10 (1H, dd, *J* = 8.0, 1.0 Hz), 7.90 (2H, d, *J* = 7.6 Hz), 7.61–7.56 (3H, m), 7.48 (1H, t, *J* = 7.4 Hz), 7.36–7.30 (2H, m), 5.30 (1H, s), 4.64 (2H, d, *J* = 5.6 Hz) ppm; ^13^C-NMR (125 MHz,(CD_3_)_2_SO) δ 161.5 (C=O), 153.1 (C),153.0 (C), 144.6 (C), 136.6 (C), 132.0 (CH), 130.0 (CH), 128.7 (CH), 123.4 (CH), 122.6 (CH), 121.5 (CH), 120.0 (CH), 117.0 (CH), 114.5 (C), 82.5 (CH), 37.8 (CH_2_) ppm; EIMS *m*/*z* 318([M^+^], 64); 290 (21); 289 (44); 261 (16); 198 (15); 159 (22); 130 (100); 77 (56); HREIMS 318.1117 (calcd. for C_18_H_14_N_4_O_2_ [M^+^] 318.1117); FT-IR (ATR) ν_max_ 3500, 3297, 3138, 3082, 2940, 1707, 1609, 1557, 1503, 1480, 1146, 1377, 1340, 1321, 1188, 1054, 954, 922, 861, 823 cm^−1^.

*4-(((1-(2-methoxyphenyl)-1H-1,2,3-triazol-4-yl)methyl)amino)-2H-chromen-2-one* (**9b**). Following the experimental procedure described in method B, from 68.0 mg (0.28 mmol) of 1-azido-2-methoxybenzene and 28 mg (0.14 mmol) of *N*-propargylated coumarin (**6**), 16.9 mg (32%) of compound **9b** were obtained as an amorphous white solid. m.p. 187–189 °C; ^1^H-NMR (500 MHz, (CD_3_)_2_SO) δ 8.36 (1H, s), 8.09 (1H, t, *J* = 5.5 Hz), 8.01 (1H, dd, *J* = 8.1, 1.2 Hz), 7.54 (1H, dd, *J* = 7.9, 1.6 Hz), 7.51 (1H, dd, *J* = 11.3, 4.2 Hz), 7.44 (1H, t, *J* = 7.9 Hz), 7.27–7.20 (m, 3H), 7.06 (1H, td, *J* = 7.7, 1.0 Hz, ), 5.30 (1H, s), 4.56 (2H, d, *J* = 5.7 Hz), 3.77 (3H, s) ppm; ^13^C-NMR (125 MHz, (CD_3_)_2_SO) δ 161.2 (C=O), 153.0 (C), 152.8 (C), 151.4 (C), 142.9 (C), 131.7 (CH), 130.4 (CH), 125.7 (C), 125.4 (CH), 125.1 (CH), 123.1 (CH), 122.4 (CH), 120.8 (CH), 116.7 (CH), 114.4 (C), 113.0 (CH), 82.5 (CH), 56.0 (CH_3_), 37.5 (CH_2_) ppm; EIMS *m*/*z* 348 ([M^+^], 20); 320 (17); 319 (20); 160 (100); 159 (20); 145 (11); 120 (11); 77 (19); HREIMS 348.1228 (calcd. for C_19_H_16_N_4_O_3_ [M^+^] 348.1222); FT-IR (ATR) ν_max_ 3524, 3318, 3173, 3085, 2944, 2847, 2376, 1649, 1607, 1555, 1505, 1476, 1446, 1384, 1327, 1290, 1260, 1244, 1197, 1120, 1044, 1017, 957, 937, 865 cm^−1^.

*4-(((1-(3-Methoxyphenyl)-1H-1,2,3-triazol-4-yl)methyl)amino)-2H-chromen-2-one* (**9c**). Following the experimental procedure described in method B, from 42.3 mg (0.28 mmol) of 1-azido-3-methoxybenzene and 28 mg (0.14 mmol) of *N*-propargylated coumarin (**6**), 26.9 mg (51%) of compound **9c** were obtained as an amorphous white solid. m.p. 246–248 °C; ^1^H-NMR (500 MHz, (CD_3_)_2_SO) δ 8.71 (1H, s), 8.08 (1H, t, *J* = 5.5 Hz), 8.02 (1H, dd, *J* = 8.1, 1.2 Hz), 7.52 (1H, t, *J* = 7.8 Hz), 7.44–7.36 (3H, m), 7.27 (1H, d, *J* = 0.8 Hz), 7.24 (1H, t, *J* = 7.7 Hz), 6.97 (1H, dt, *J* = 6.8, 2.5 Hz), 5.25 (1H, s), 4.57 (2H, d, *J* = 5.6 Hz), 3.78 (3H, s) ppm; ^13^C-NMR (125 MHz, (CD_3_)_2_SO) δ 161.2 (C=O), 160.1 (C), 153.0 (C), 152.7 (C), 144.3 (C), 137.5 (C), 131.7 (CH), 130.6 (CH), 123.1 (CH), 123.0 (CH), 121.5 (CH), 116.7 (CH), 114.4 (C), 114.2 (CH), 111.9 (CH), 105.7 (CH), 82.5 (CH), 55.5 (CH_3_), 37.7 (CH_2_) ppm; EIMS *m*/*z* 348 ([M^+^], 40); 319 (25); 291 (10); 198 (11); 161 (13); 160 (100); 159 (30); 123 (11); 107 (24); 92 (19); 77 (26); HREIMS 348.1221 (calcd. for C_19_H_16_N_4_O_3_ [M^+^] 348.1222); FT-IR (ATR) ν_max_ 3297, 3143, 3085, 3000, 2936, 2830, 1708, 1609, 1558, 1483, 1446, 1373, 1322, 1246, 1192, 1158, 1142, 1118, 1048, 954, 922, 859, 846, 819 cm^−1^.

*4-(((1-(4-Methoxyphenyl)-1H-1,2,3-triazol-4-yl)methyl)amino)-2H-chromen-2-one* (**9d**). Following the experimental procedure described in method B, from 42.3 mg (0.28 mmol) of 1-azido-4-methoxybenzene and 28 mg (0.14 mmol) of *N*-propargylated coumarin (**6**), 29.6 mg (56%) of compound **9d** were obtained as an amorphous white solid. m.p. 246–248 °C; ^1^H-NMR (500 MHz, (CD_3_)_2_SO) δ 8.58 (1H, s), 8.07 (1H, t, *J* = 5.4 Hz), 8.01 (1H, dd, *J* = 8.1, 1.1 Hz), 7.74–7.69 (2H, m), 7.52 (1H, t, *J* = 7.8 Hz), 7.24 (2H, dd, *J* = 14.4, 7.6 Hz), 7.07–7.01 (2H, m), 5.25 (1H, s), 4.55 (2H, d, *J* = 5.6 Hz), 3.75 (3H, s) ppm; ^13^C-NMR (125 MHz, (CD_3_)_2_SO) δ 161.2 (C=O), 159.2 (C), 153.0 (C), 152.8 (C), 144.1 (C), 131.7 (CH), 130.0 (C), 123.1 (CH), 122.4 (CH), 121.6 (2CH), 121.3 (CH), 116.7 (CH), 114.7 (2CH), 114.4 (C), 82.5 (CH), 55.4 (CH_3_), 37.7 (CH_2_) ppm; EIMS *m*/*z* 348 ([M^+^], 21); 320 (14); 319 (22); 161 (12); 160 (100); 159 (26); 123 (10); 77 (15); HREIMS 348.1225 (calcd. for C_19_H_16_N_4_O_3_ [M^+^] 348.1222); FT-IR (ATR) ν_max_ 3528, 3285, 3137, 3081, 3067, 3003, 2945, 2924, 2829, 2310, 2051, 1700, 1609, 1556, 1517, 1446, 1378, 1307, 1247, 1187, 1140, 1055, 1041, 989, 953, 920, 860, 822 cm^−1^.

*4-(((1-(3-Fluoro-4-methoxyphenyl)-1H-1,2,3-triazol-4-yl)methyl)amino)-2H-chromen-2-one* (**9e**). Following the experimental procedure described in method B, from 47.4 mg (0.28 mmol) of 1-azido-3-fluoro-4-methoxybenzene and 28 mg (0.14 mmol) of *N*-propargylated coumarin (**6**), 27.3 mg (49 %) of compound **9e** were obtained as an amorphous white solid. m.p. 204–205 °C; ^1^H-NMR (500 MHz, (CD_3_)_2_SO) δ 8.76 (1H, s), 8.29 (1H, t, *J* = 5.7 Hz), 8.09 (1H, dd, *J* = 8.1, 1.2 Hz), 7.87 (1H, dd, *J* = 12.1, 2.6 Hz), 7.72 (1H, ddd, *J* = 8.9, 2.5, 1.4 Hz), 7.60 (1H, t, *J* = 7.8 Hz), 7.39–7.30 (3H, m), 5.28 (1H, s), 4.62 (2H, d, *J* = 5.6 Hz), 3.90 (3H, s); ^13^C-NMR (125 MHz, (CD_3_)_2_SO) δ 162.4 (C=O), 153.7 (C), 153.4 (C), 151.6 (C, *J*^1^_C–F_ = 247.8 Hz), 147.7 (C, *J*^2^_C–F_ = 20.3 Hz), 144.9 (C), 132.7 (CH), 129.9 (C, *J*^3^_C–F_ = 9.0 Hz), 124.1 (CH), 122.8 (CH), 122.1 (CH), 117.4 (CH), 117.0 (CH,*J*^3^_C–F_ = 2.8 Hz), 114.9 (C), 114.7 (CH), 109.2 (CH, *J*^2^_C–F_ = 22.5 Hz), 82.8 (CH), 56.7 (CH_3_), 38.0 (CH_2_) ppm; EIMS *m*/*z* 366 ([M^+^], 30); 338 (16); 337 (22); 198 (15); 179 (13); 178 (100); 159 (22); HREIMS 366.1138 (calcd. for C_19_H_15_N_4_O_3_F [M^+^] 366.1128); FT-IR (ATR) ν_max_ 3301, 3133, 3078, 3009, 2936, 2849, 1699, 1609, 1557, 1518, 1477, 1446, 1377, 1317, 1184, 1123, 1082, 1053, 953, 921, 884, 859, 817 cm^−1^.

*4-(((1-(4-Fluoro-phenyl)-1H-1,2,3-triazol-4-yl)methyl)amino)-2H-chromen-2-one* (**9f**). Following the experimental procedure described in method A, from 34.8 mg (0.24mmol) of 4-fluoro-phenyl boronic acid and 28 mg (0.14 mmol) of *N*-propargylated coumarin (**6**), 15.2 mg (30 %) of compound **9f** were obtained as an amorphous white solid. m.p. 212–213 °C; ^1^H-NMR (500 MHz, (CD_3_)_2_SO) δ 8.79 (1H, s), 8.32 (1H, t, *J =* 6.0 Hz), 8.16 (1H, d, *J =* 7.8 Hz), 7.97–7.91 (2H, m), 7.61 (1H, t, *J =* 8.4 Hz), 7.45 (2H, t, *J =* 8.8 Hz), 7.35–7.30 (2H, m), 5.30 (1H, s), 4.6 (2H, d, *J =* 5.5 Hz) ppm; ^13^C-NMR (125 MHz, (CD_3_)_2_SO) δ 161.4 (C=O), 153.1 (C), 153.0 (C), 144.7 (C), 133.1 (C), 132.0 (CH), 123.4 (CH), 122.6 (CH), 122.3 (2CH, *J*^3^_C–F_ = 9.1 Hz), 121.7 (C), 116.9 (CH), 116.7 (2CH, *J*^2^_C–F_ = 23.9 Hz), 114.5 (C), 82.6 (CH), 37.4 (CH_2_) ppm; EIMS *m*/*z* 336 ([M^+^], 54); 308 (19); 307 (36); 198 (15); 159 (27); 148 (100); 95 (38); HREIMS 336.1030 (calcd. for C_18_H_13_N_4_O_2_F [M^+^] 336.1023); FT-IR (ATR) ν_max_ 3537, 3417, 3307, 3143, 3084, 2454, 2288, 2167, 2051, 1985, 1707, 1610, 1558, 1541, 1516, 1481, 1447, 1377, 1230, 1183, 1051, 993, 957, 920, 825 cm^−1^.

*4-(((1-(3-Trifluoromethylphenyl)-1H-1,2,3-triazol-4-yl)methyl)amino)-2H-chromen-2-one* (**9g**). Following the experimental procedure described in method B, from 53.2 mg (0.28 mmol) of 1-azido-3-trifluoromethylbenzene and 28 mg (0.14 mmol) of *N*-propargylated coumarin (**6**), 26.2 mg (45 %) of compound **9g** were obtained as an amorphous white solid. m.p. 231–232 °C; ^1^H-NMR (500 MHz, (CD_3_)_2_SO) δ 9.00 (1H, s), 8.32 (1H, t, *J* = 5.7 Hz), 8.29–8.24 (2H, m), 8.09 (1H, dd, *J* = 8.1, 1.2 Hz), 7.88–7.80 (2H, m), 7.60 (1H, dd, *J* = 15.6, 1.4 Hz), 7.34 (2H, ddd, *J* = 9.2, 8.2, 1.0 Hz), 5.29 (1H, s), 4.66 (2H, d, *J* = 5.7 Hz) ppm; ^13^C-NMR (125MHz, (CD_3_)_2_SO) δ 161.5 (C=O), 153.1 (C), 153.0 (C), 145.0 (C), 137.1 (C), 132.0 (CH), 131.4 (CH), 130.5 (C, *J*^2^_C–F_ = 32.5 Hz), 125.2(CH, *J*^3^_C–F_ = 3.1 Hz), 123.9 (CH), 123.6 (C, *J*^1^_C–F_ = 272.5 Hz), 123.4 (CH), 122.6 (CH), 121.8 (CH), 117.0 (CH), 116.7 (CH, *J*^3^_C-F_= 3.8 Hz), 114.5 (C), 82.6 (CH), 37.8 (CH_2_); EM-IE *m*/*z* 386 ([M^+^], 72); 358 (26); 357 (50); 198 (100); 159 (29); 145 (49); HREIMS 386.0977 (calcd. for C_19_H_13_N_4_O_2_F_3_ [M^+^] 386.0991); FT-IR (ATR) ν_max_ 3425, 3318, 3142, 3085, 2942, 1701, 1610, 1557, 1540, 1482, 1448, 1377, 1342, 1321, 1298, 1266, 1248, 1172, 1142, 1110, 1070, 1046, 955, 923, 896, 861, 819 cm^–1^.

*4-(((1-(3-Nitrophenyl)-1H-1,2,3-triazol-4-yl)methyl)amino)-2H-chromen-2-one* (**9h**). Following the experimental procedure described in method B, from 46.6 mg (0.28 mmol) of 1-azido-3-nitrobenzene and 28 mg (0.14 mmol) of *N*-propargylated coumarin (**6**), 21.2 mg (39 %) of compound **9h** were obtained as an amorphous white solid. m.p. 240–241 °C; ^1^H-NMR (600 MHz, (CD_3_)_2_SO) δ 9.06 (1H, s), 8.73 (1H, t, *J* = 1.9 Hz), 8.42 (1H, d, *J* = 7.8 Hz), 8.32 (2H, dd, *J* = 8.2, 2.1 Hz), 8.10 (1H, d, *J* = 7.9 Hz), 7.93–7.83 (1H, m), 7.61 (1H, t, *J* = 7.4 Hz), 7.37–7.27 (2H, m), 5.29 (1H, s), 4.67 (2H, d, *J* = 5.4 Hz) ppm; ^13^C-NMR (150 MHz, (CD_3_)_2_SO) δ 161.4 (C=O), 153.1 (C), 153.0 (C), 148.6 (C), 145.2 (C), 137.1 (C), 132.0 (CH), 131.5 (CH), 126.0 (CH), 123.4 (CH), 123.1 (CH), 122.5 (CH), 122.0 (CH), 116.9 (CH), 114.7 (CH), 114.5 (C), 82.61 (CH), 37.7 (CH_2_) ppm; EIMS *m*/*z* 363 ([M^+^], 48); 335 (31); 334 (69); 333 (100); 286 (23); 242 (30); 198 (28); 197 (31); 175 (40); 161 (38); 159 (31); 129 (40); 92 (27); 76 (30); 65 (27); HREIMS 363.0983 (calcd. for C_18_H_13_N_5_O_4_ [M^+^] 363.0968); FT-IR (ATR) ν_max_ 3426, 3137, 3106, 3082, 2927, 1707, 1614, 1561, 1531, 1481, 1448, 1352, 1315, 1269, 1189, 1142, 1119, 1043, 1001, 960, 925, 863, 818 cm^−1^.

*4-(((1-(1H-Indole-5-yl)-1H-1,2,3-triazol-4-yl)methyl)amino)-2H-chromen-2-one* (**9i**). Following the experimental procedure described in method B, from 44.9 mg (0.28 mmol) of 5-azido-1*H*-indole and 28.0 mg (0.14 mmol) of *N*-propargylated coumarin (**6**), 20.7 mg (37 %) of compound **9i** were obtained as an amorphous white solid. m.p. 236–237 °C;^1^H-NMR (500 MHz, (CD_3_)_2_SO) δ 11.42 (1H, bs), 8.72 (1H, s), 8.29 (1H, t, *J* = 5.6 Hz), 8.11 (1H, d, *J* = 7.2 Hz), 8.00 (1H, s), 7.63–7.53 (3H, m), 7.50 (1H, s), 7.33 (2H, dd, *J* = 12.4, 7.8 Hz), 6.55 (1H, s, *J* = 2.1 Hz), 5.34 (1H, s), 4.63 (2H, d, *J* = 5.6 Hz) ppm; ^13^C-NMR (125 MHz, (CD_3_)_2_SO)δ162.4 (C=O), 161.5 (C), 153.1 (C), 153.0(C), 144.1 (C), 135.5 (C), 132.0 (CH), 129.5 (C), 127.7 (CH), 127.6 (C), 123.4 (CH), 122.6 (CH), 121.9 (CH), 116.9 (CH), 114.5 (C), 114.2 (CH), 112.3 (CH), 111.9 (CH), 101.9 (CH), 82.5 (CH), 37.9 (CH_2_); EIMS *m*/*z* 357 ([M^+^], 27); 329 (14); 328 (23); 170 (16); 169 (100); 168 (30); 156 (23); 132 (21); 116 (57); 115 (16); 89 (19); HREIMS 357.1217 (calcd. for C_20_H_15_N_5_O_2_ [M^+^] 357.1226); FT-IR (ATR) ν_max_ 3522, 3397, 3324, 1682, 1616, 1562, 1486, 1354, 1092, 1053, 886 cm^−1^.

*4-(((1-(Furan-3-yl)-1H-1,2,3-triazol-4-yl)methyl)amino)-2H-chromen-2-one* (**9j**). Following the experimental procedure described in method A, from 21.3 mg (0.24 mmol) of 3-furyl boronic acid and 28 mg (0.14 mmol) of *N*-propargylated coumarin (**6**), 5.4 mg (12%) of compound **9j** were obtained as an amorphous white solid. m.p. 198–199 °C; ^1^H-NMR (500 MHz, (CD_3_)_2_SO) δ 8.58 (1H, s), 8.42 (1H, d, *J* = 0.8 Hz), 8.30 (1H, t, *J* = 5.6 Hz), 8.08 (1H, d, *J* = 6.9 Hz), 7.86 (1H, t, *J* = 1.8 Hz), 7.60 (1H, t, *J* = 7.8 Hz), 7.32 (2H, dd, *J* = 8.1, 4.6 Hz), 7.12 (1H, dd, *J* = 2.0, 0.8 Hz), 5.27 (1H, s), 4.62 (2H, d, *J* = 5.6 Hz) ppm; ^13^C-NMR (125 MHz, (CD_3_)_2_SO) δ 161.4 (C=O), 153.1 (C), 153.0 (C), 144.8 (CH), 144.3 (C), 133.9 (CH), 132.0 (CH), 126.0 (C), 123.4 (CH), 122.6 (CH), 122.2 (CH), 117.0 (CH), 114.5 (C), 105.1 (CH), 82.5 (CH), 37.7 (CH_2_) ppm; EIMS *m*/*z* 308 ([M^+^], 99); 120 (67); 93 (28); 91 (12); 66 (23); 65 (100); 58 (17); HREIMS 308.1000 (calcd. for C_16_H_12_N_4_O_3_ [M^+^] 308.0988); FT-IR (ATR) ν_max_ 3293, 3134, 3083, 2924, 2853, 2285, 1706, 1610, 1557, 1480, 1446, 1378, 1323, 1262, 1230, 1191, 1143, 1118, 1089, 1039, 1017, 955, 921, 867, 821 cm^−1^.

*4-(((1-Undecyl-1H-1,2,3-triazol-4-yl)methyl)amino)-2H-chromen-2-one* (**9k**). Following the experimental procedure described in method B, from 56.1 mg (0.28 mmol) of 1-azido-undecane and 28 mg (0.14 mmol) of *N*-propargylated coumarin (**6**), 50.3 mg (84%) of compound **9k** were obtained as an amorphous white solid. m.p. 154–156 °C; ^1^H-NMR (500 MHz, (CD_3_)_2_SO) δ 8.08–7.93 (3H, m), 7.51 (1H, t, *J* = 7.7 Hz), 7.25 (1H, s), 7.22 (1H, t, *J* = 7.9 Hz), 5.18 (1H, s), 4.46 (2H, d, *J* = 5.6 Hz), 4.24 (2H, t, *J* = 7.0 Hz), 1.80–1.59 (2H, m), 1.14 (16H, s), 0.78 (3H, t, *J* = 6.9 Hz) ppm; ^13^C-NMR (125 MHz, (CD_3_)_2_SO) δ 161.1 (C=O), 153.0 (C), 152.7 (C), 143.1 (C), 131.6 (CH), 123.1 (CH), 122.8 (CH), 122.3 (CH), 116.7 (CH), 114.4 (C), 82.3 (CH), 49.2 (CH_2_), 37.7 (CH_2_), 31.0 (CH_2_), 29.4 (CH_2_), 28.7 (CH_2_), 28.6 (CH_2_), 28.6 (CH_2_), 28.4 (CH_2_), 28.1 (CH_2_), 25.6 (CH_2_), 21.8 (CH_2_), 13.6 (CH_3_) ppm; EIMS *m*/*z* 396 ([M^+^], 88); 395 (16); 368 (40); 367 (100); 339 (15); 297 (18); 283 (17); 235 (20); 227 (37); 199 (23); 198 (21); 162 (19); 57 (19); 55 (23); HREIMS 396.2504 (calcd. for C_23_H_32_N_4_O_2_ [M^+^] 396.2525); FT-IR (ATR) ν_max_ 3523, 3399, 3325, 3127, 3069, 2955, 2920, 2849, 1679, 1607, 1553, 1481, 1465, 1446, 1376, 1096, 1055 cm^−1^.

*4-(((1-Benzyl-1H-1,2,3-triazol-4-yl)methyl)amino)-2H-chromen-2-one* (**9l**). Following the experimental procedure described in method B, from 56.1 mg (0.28 mmol) of azidomethyl-benzene and 28 mg (0.14 mmol) of *N*-propargylated coumarin (**6**), 45.5 mg (91%) of compound **9l** were obtained as an amorphous white solid. m.p. 217–219 °C; ^1^H-NMR (500 MHz, (CD_3_)_2_SO) δ 8.03 (2H, d, *J* = 8.7 Hz), 7.96 (1H, dd, *J* = 8.0, 1.2 Hz), 7.50 (1H, t, *J* = 7.8 Hz), 7.31–7.19 (7H, m), 5.50 (2H, s), 5.19 (1H, s), 4.47 (2H, d, *J* = 5.8 Hz) ppm; ^13^C-NMR (125 MHz, (CD_3_)_2_SO) δ 161.2 (C=O), 153.0 (C), 152.8 (C), 143.5 (C), 135.9 (C), 131.7 (CH), 128.5 (2CH), 127.9 (CH), 127.7 (2CH), 123.2 (CH), 123.1 (CH), 122.3 (CH), 116.7 (CH), 114.3 (C), 82.4 (CH), 52.7 (CH_2_), 37.6 (CH_2_) ppm; EIMS *m*/*z* 332 ([M^+^], 41); 303 (14); 213 (18); 144 (13); 91 (100); 65 (11); HREIMS 332.1279 (calcd. for C_19_H_16_N_4_O_2_ [M^+^] 332.1273); FT-IR (ATR) ν_max_ 3283, 3141, 3075, 2922, 2852, 2366, 2323, 1697, 1608, 1557, 1482, 1446, 1380, 1322, 1262, 1222, 1188, 1124, 1058, 955, 920, 861, 827 cm^−1^.

*4-(((1-Tetradecyl-1H-1,2,3-triazol-4-yl)methyl)amino)-2H-chromen-2-one* (**9m**). Following the experimental procedure described in method B, from 68.0 mg (0.28 mmol) of 1-azido-tetradecane and 28 mg (0.14 mmol) of *N*-propargylated coumarin (**6**), 63.5 mg (96%) of compound **9m** were obtained as an amorphous white solid. m.p. 156–157 °C; ^1^H-NMR (500 MHz, (CD_3_)_2_SO) δ 8.23 (1H, t, *J* = 5.7 Hz), 8.07 (1H, s), 8.05 (1H, d, *J* = 8.1 Hz), 7.58 (1H, d, *J* = 7.5 Hz), 7.33–7.29 (2H, m), 5.24 (1H, s), 4.53 (2H, d, *J* = 5.7 Hz), 4.31 (2H, t, *J* = 7.0 Hz), 1.75 (2H, t, *J* = 7.3 Hz ), 1.23–1.13 (22H, m), 0.85 (3H, t, *J* = 6.9 Hz) ppm; ^13^C-NMR (150 MHz, (CD_3_)_2_SO) δ 161.8 (C=O), 153.5 (C), 153.3 (C), 143.7 (C), 132.4 (CH), 123.8 (CH), 123.5 (CH), 122.9 (CH), 117.4 (CH), 114.9 (C), 82.8 (CH), 49.7 (CH_2_), 31.7 (CH_2_), 30.1 (CH_2_), 29.5 (2CH_2_), 29.4 (2CH_2_), 29.3 (2CH_2_), 29.1 (CH_2_), 28.7 (CH_2_), 26.2 (CH_2_), 22.5 (CH_2_), 14.4 (CH_3_) ppm; EIMS *m*/*z* 438 ([M^+^], 92); 410 (55); 409 (100); 367 (37); 227 (51); 199 (44); 198 (60); 162 (39); 57 (42); 55 (47); HREIMS 438.2978 (calcd. for C_26_H_38_N_4_O_2_ [M^+^] 438.2995); FT-IR (ATR) ν_max_ 3320, 3127, 3070, 2918, 2848, 2416, 2167, 2051, 1983, 1658, 1606, 1550, 1467, 1377, 1322, 1258, 1202, 1150, 1118, 1055, 966, 938, 867, 835 cm^−1^.

### 3.2. Microbial Strains

*Staphylococcus aureus* (ATCC 6538), *Enterococcus faecalis* (PCM 2673), *Escherichia coli* (ATCC 8739), *Klebsiella pneumoniae* (PCM1, *Pseudomonas aeruginosa* (PCM 2562) and yeast *Candida albicans* (ATCC 10231) were obtained from the Department of Molecular Biology, The John Paul II Catholic University of Lublin, Poland.

### 3.3. MIC Determination

The in vitro antimicrobial studies were carried out with the microbroth dilution method against test organisms, as described previously [59,60]. The bacterial strains were inoculated in Mueller Hinton Broth medium (Biocorp, Warsaw, Poland) and the *Candida* strain was inoculated in Sabouraud Dextrose liquid medium (Biocorp, Poland) and incubated at 37 °C and at 30 °C, respectively, with vigorous shaking (200 rpm) for 24 h. Bacterial cell suspensions at initial inoculums of 5 × 10^5^ in Mueller-Hinton liquid medium and adequate yeast suspensions at initial inoculums of 3 × 10^3^ cfu/mL in Sabouraud Dextrose Broth were exposed to the examined compound at relevant concentrations (range 0.001–2 mg/mL) for 24 h at 37 °C for the bacteria and for 48 h at 30 °C in the case of the fungi. Simultaneously, the standard antibiotics, chloramphenicol for antibacterial activity and ketoconazole for antifungal activity (as a positive control), were tested against the pathogens. The MIC was the lowest concentration of the compounds that inhibited the visible growth of the microorganism. The experiments were performed in triplicate.

### 3.4. Haemolytic Assay

Haemolytic properties of the selected compounds were determined according to the method described previously [45]. The human blood samples were centrifuged at 500× *g* for 10 min at 4 °C and the supernatant was discarded. Next, the erythrocytes were resuspended with PBS buffer (10 mM phosphate, pH 7.5; 150 mM NaCl) and centrifuged as previously. The washing procedure was repeated until a transparent supernatant was obtained. The washed erythrocytes were finally resuspended in PBS buffer to a final concentration of 2%. Simultaneously, appropriate concentrations (5, 10, 25, 50, 100 and 500 µg/mL for **8a**, **8f**, **9h** and **9k**, or 2, 5, 12.5, 50, 125 and 250 µg/mL for **8b**) of the examined compounds were prepared in a final volume of 50 mL DMSO. The compounds prepared in this way were mixed with 450 mL of 2% erythrocyte suspension and incubated for 1 h at 37 °C. Then, the samples were centrifuged at 5000× *g* for 10 min and absorbance at wavelength 415 nm was measured.

## 4. Conclusions

In conclusion, twenty-eight coumarin-triazole conjugates were synthesized through a copper(I)-catalysed Huisgen 1,3-dipolar cycloaddition reaction of the corresponding *O*-propargylated coumarin (**3**) or *N*-propargylated coumarin (**6**) with alkyl or aryl azides. Five of them (**8a**, **8b**, **8f**, **9h** and **9k**) displayed promising activity against *Enterococcus faecalis* at MICs ranging from 12.5 to 50.0 µg/mL. Compound **8b** having a 2-OMe-Ph group attached at the triazol nucleus and an –OCH_2_– linker was the best of the series. The most active compounds showed minimal toxicity towards human blood cells.

## Figures and Tables

**Table 1:** Synthesized azides **7a**–**7j**.

Entry	Azide	Ar	Yield ^a^
1	**7a**	Ph	58
2	**7b**	2-OMe–Ph	61
3	**7c**	3-OMe–Ph	97
4	**7d**	4-OMe–Ph	96
5	**7e**	3-F, 4-OMe–Ph	96
6	**7f**	4-F–Ph	41
7	**7g**	3-CF_3_–Ph	31
8	**7h**	3-NO_2_–Ph	82
9	**7i**	5-*1H*-indol	35
10	**7j**	3-furyl	- ^b^

^a^ Isolated yield; ^b^ It was not isolated but was reacted in situ.

**Table 2:** Synthesized azides **7k**–**7m**.

Entry	Azide	R	Yield ^a^
1	**7k**	(CH_2_)_10_CH_3_	86
2	**7l**	(CH_2_)_13_CH_3_	73
3	**7m**	CH_2_Ph	82

^a^ Isolated yield; ^b^ MW irradiation at 120 °C for 35 min was used.

**Table 3:** Antimicrobial activity (MIC µg/mL) of the synthesized compounds.

Compound	*Candida albicans*	*Staphylococcus aureus*	*Enterococcus faecalis*	*Escherichia coli*	*Klebsiella pneumoniae*	*Pseudomonas aeruginosa*
**8a**	1600	400	50	1600	-	1600
**8b**	800	200	12.5	1600	1600	1600
**8c**	1600	800	100	1600	800	1600
**8d**	-	-	200	1600	800	1600
**8e**	400	400	100	200	800	800
**8f**	1600	1600	50	1600	400	800
**8g**	800	1600	100	800	400	800
**8h**	800	400	400	1600	800	1600
**8i**	1600	1600	200	1600	1600	1600
**8j**	1600	800	800	1600	1600	1600
**8k**	-	-	400	1600	1600	1600
**8l**	-	-	400	1600	1600	800
**9a**	1600	1600	400	1600	1600	-
**9b**	-	1600	800	1600	800	1600
**9c**	200	800	400	1600	1600	800
**9d**	200	400	100	800	800	800
**9e**	200	200	100	800	800	800
**9f**	200	-	1600	1600	1600	-
**9g**	-	200	-	-	-	-
**9h**	1600	100	50	800	800	800
**9i**	1600	1600	100	1600	800	1600
**9j**	-	800	800	1600	1600	-
**9k**	1600	200	50	-	-	-
**9l**	1600	-	800	1600	1600	1600
**CAM**	n.d.	5	5	1.2	5	5
**KET**	8	n.d.	n.d.	n.d.	n.d.	n.d.

no activity; n.d.—not determined, CAM—chloramphenicol; KET—ketoconazole.

## Supplementary Materials

The following are available online: ^1^H-NMR and ^13^C-NMR spectra of compounds **6**, **8a**–**8m** and **9a**–**9m**.
