# Supplementary material for: Synthesis and Antimicrobial Activity of 4-Substituted 1,2,3-Triazole-Coumarin Derivatives
Source: Molecules. 2018 Jan 18;23(1):199. doi: 10.3390/molecules23010199 (PMC6017388; doi:10.3390/molecules23010199)

Supporting Information

Synthesis and antimicrobial activity of 4-substituted 1,2,3-triazole-coumarin derivatives

Priscila López-Rojas,[1] Monika Janeczko,[2] Konrad Kubiński,[2] Ángel Amesty,[1,*] Maciej Masłyk[2*], Ana Estévez-Braun[1,*]

[1]Instituto Universitario de Bio-Orgánica Antonio González (CIBICAN), Departamento de QuímicaOrgánica, Universidad de La Laguna, Spain

[2]Department of Molecular Biology, The John Paul II Catholic University of Lublin, Poland

Contents

^{1}HNMR and ^{13}CNMR spectra of compounds **6**, **8a-8m** and **9a-9m**

^{1}H-NMR ($CDCl_3$, 500 MHz) of compound 6.

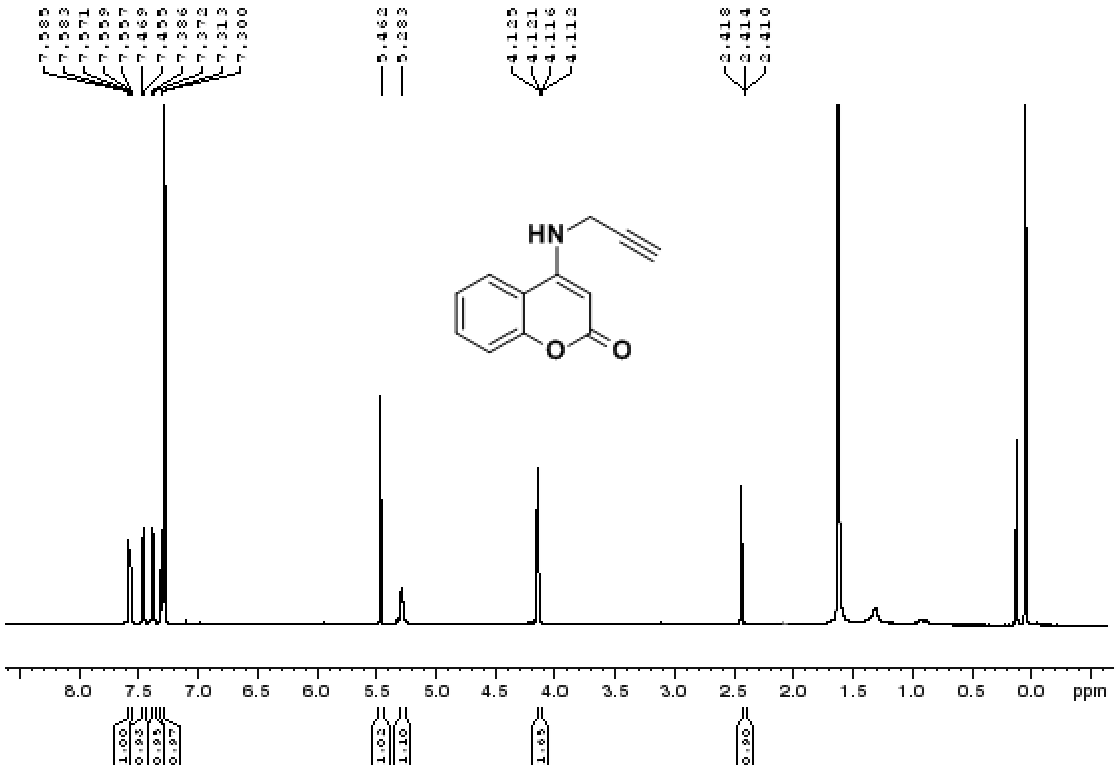

^{13}C-NMR ($CDCl_3$, 125 MHz) of compound 6.

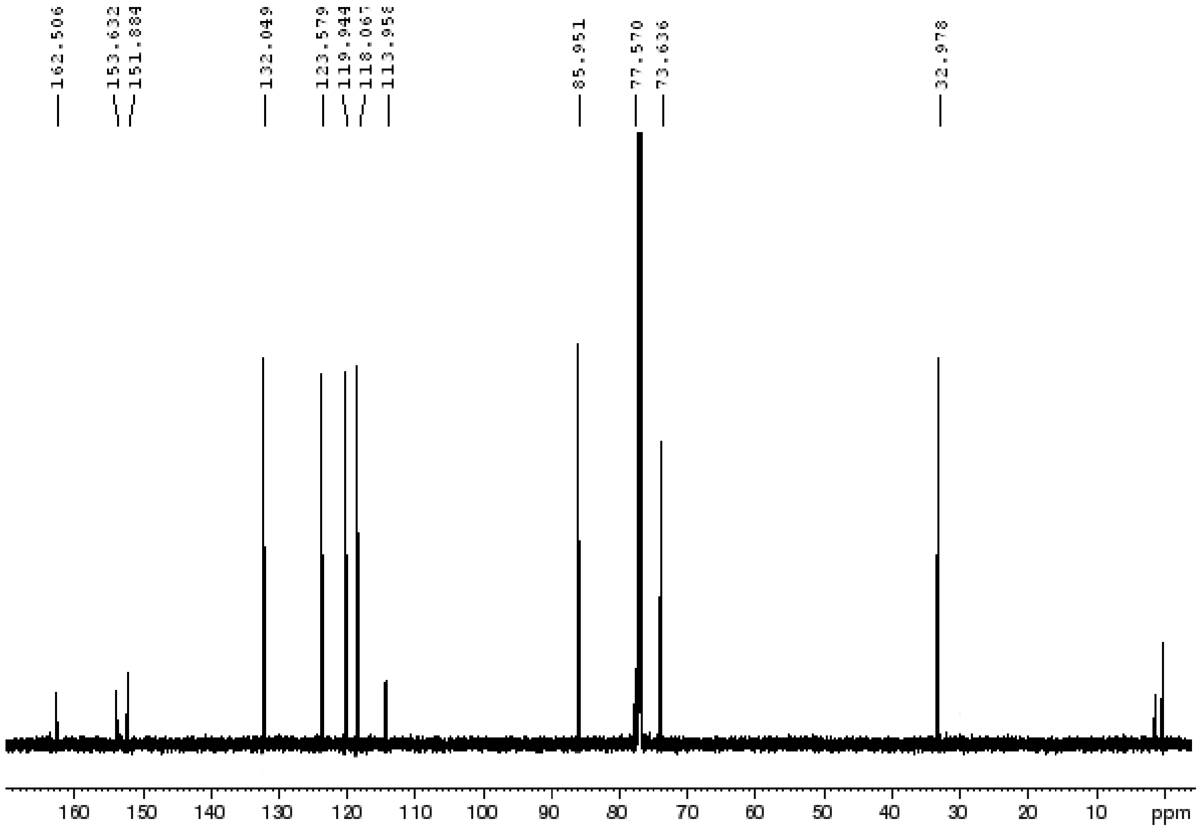

^{1}H-NMR ((CD3)2SO, 500 MHz) of compound 8a.

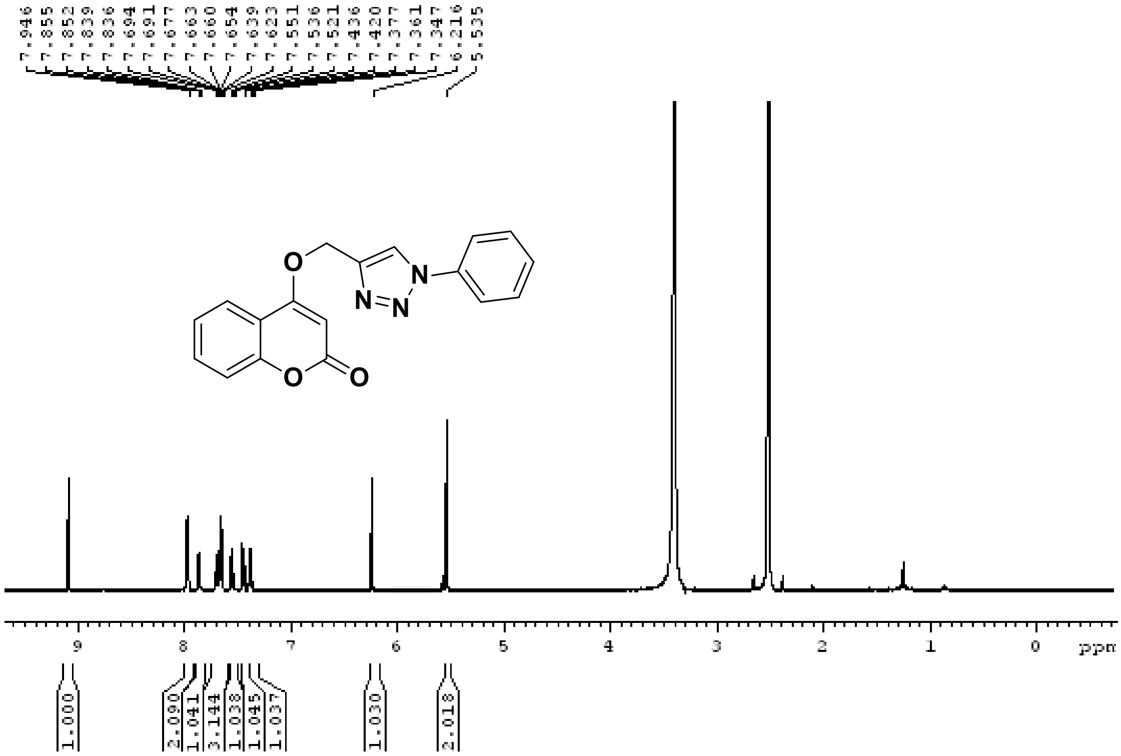

^{13}C-NMR ((CD3)2SO, 125 MHz) of compound 8a.

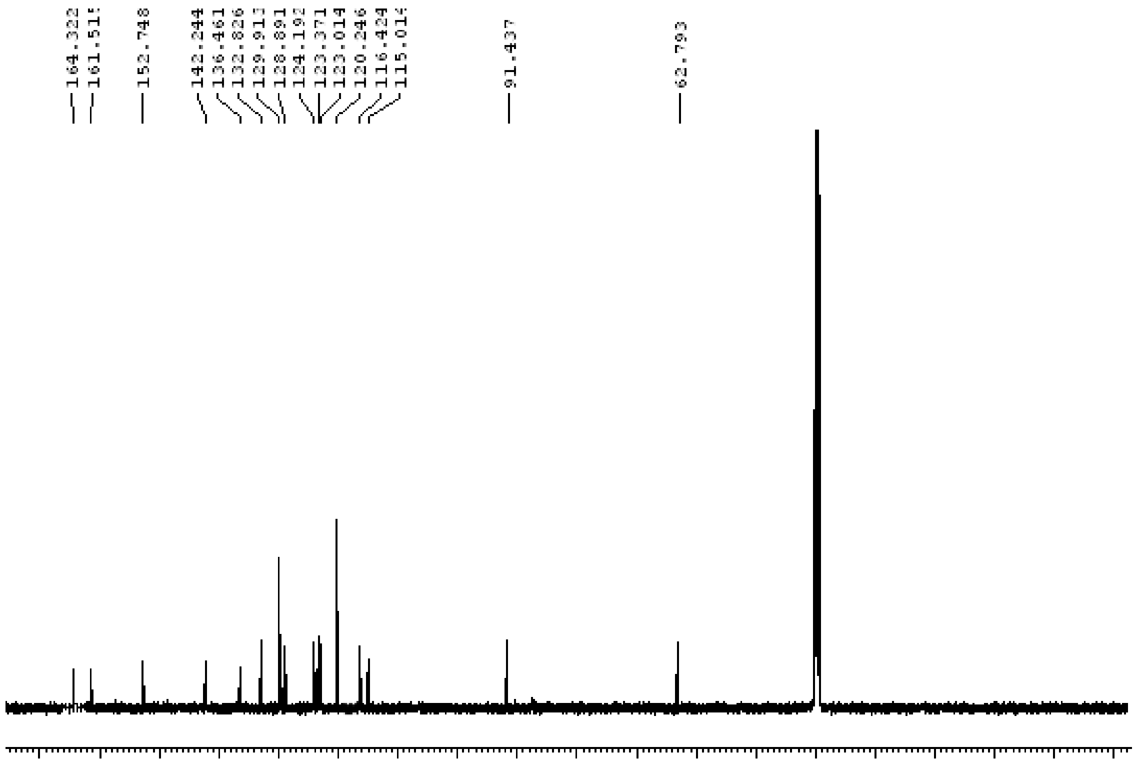

^{1}H-NMR ($CDCl_3$, 500 MHz) of compound 8b.

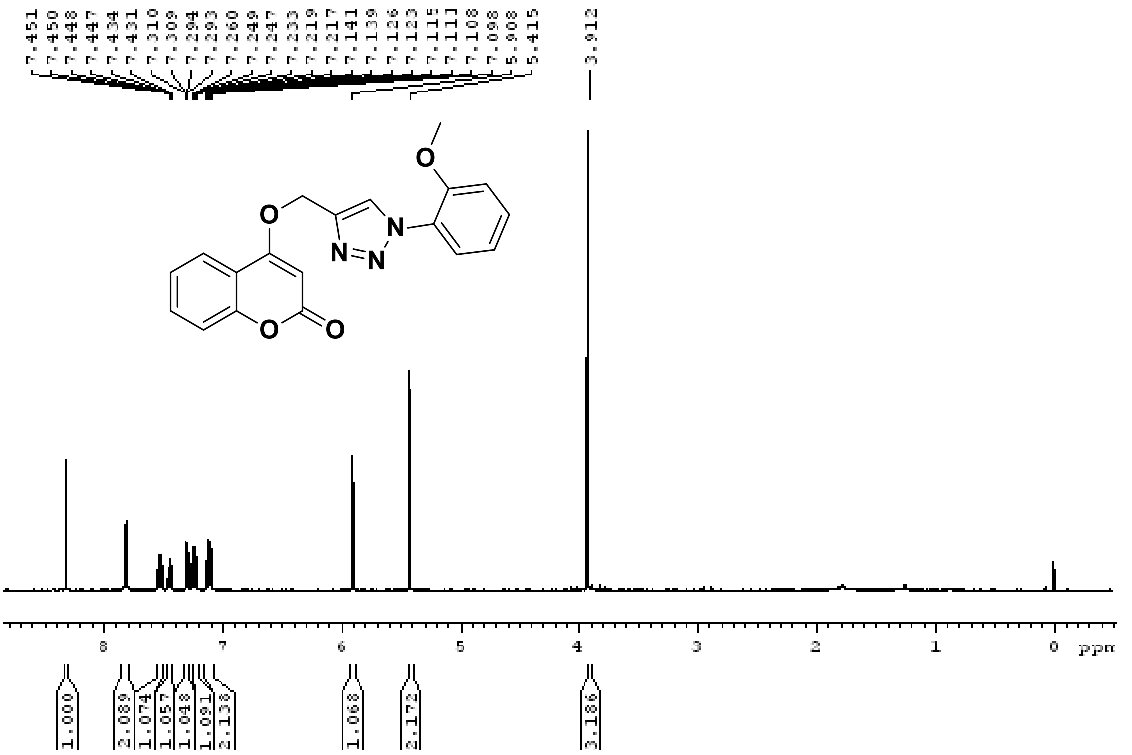

^{13}C-NMR ($CDCl_3$, 125 MHz) of compound 8b.

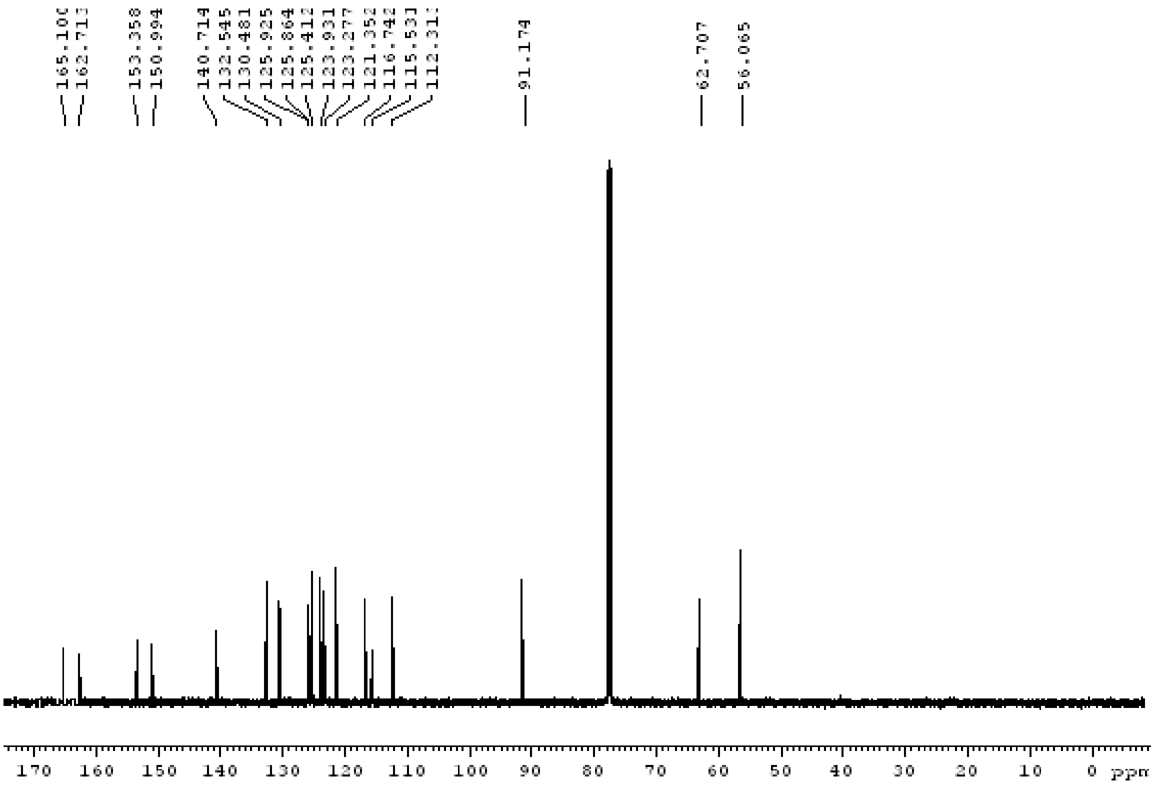

^{1}H-NMR ($CDCl_3$, 500 MHz) of compound 8c.

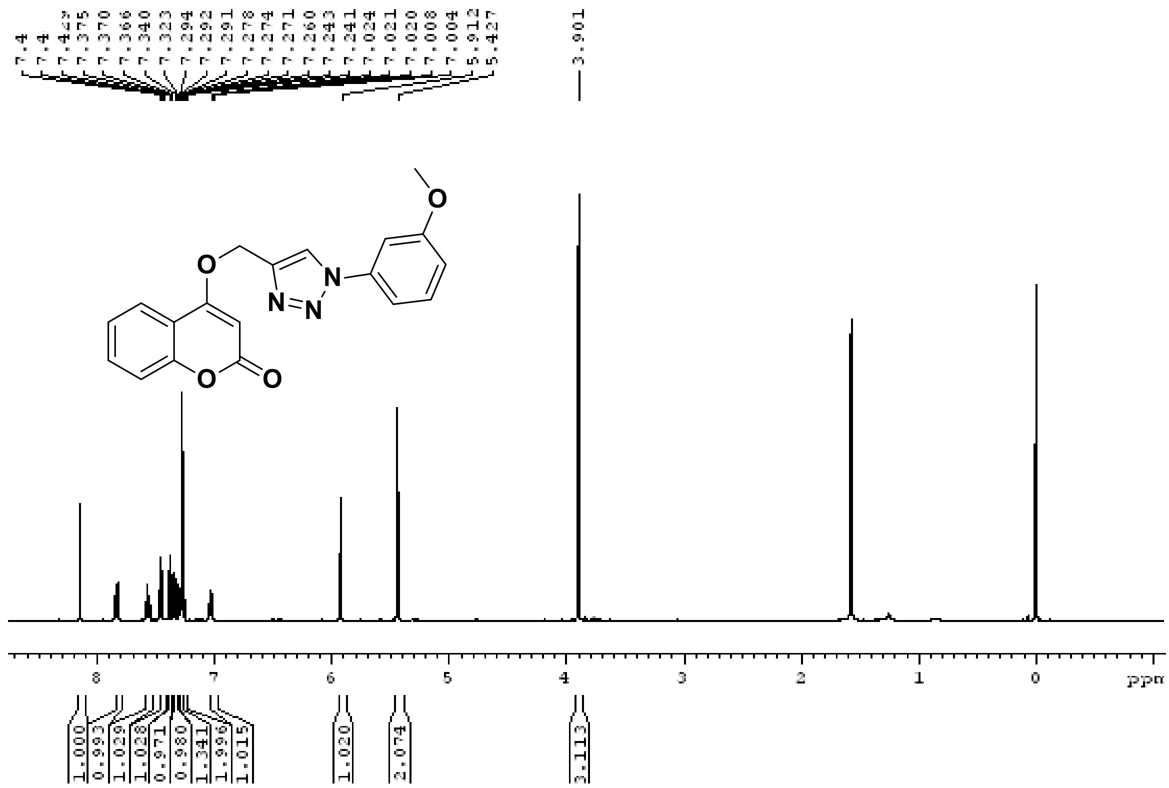

^{13}C-NMR ($CDCl_3$, 125 MHz) of compound 8c.

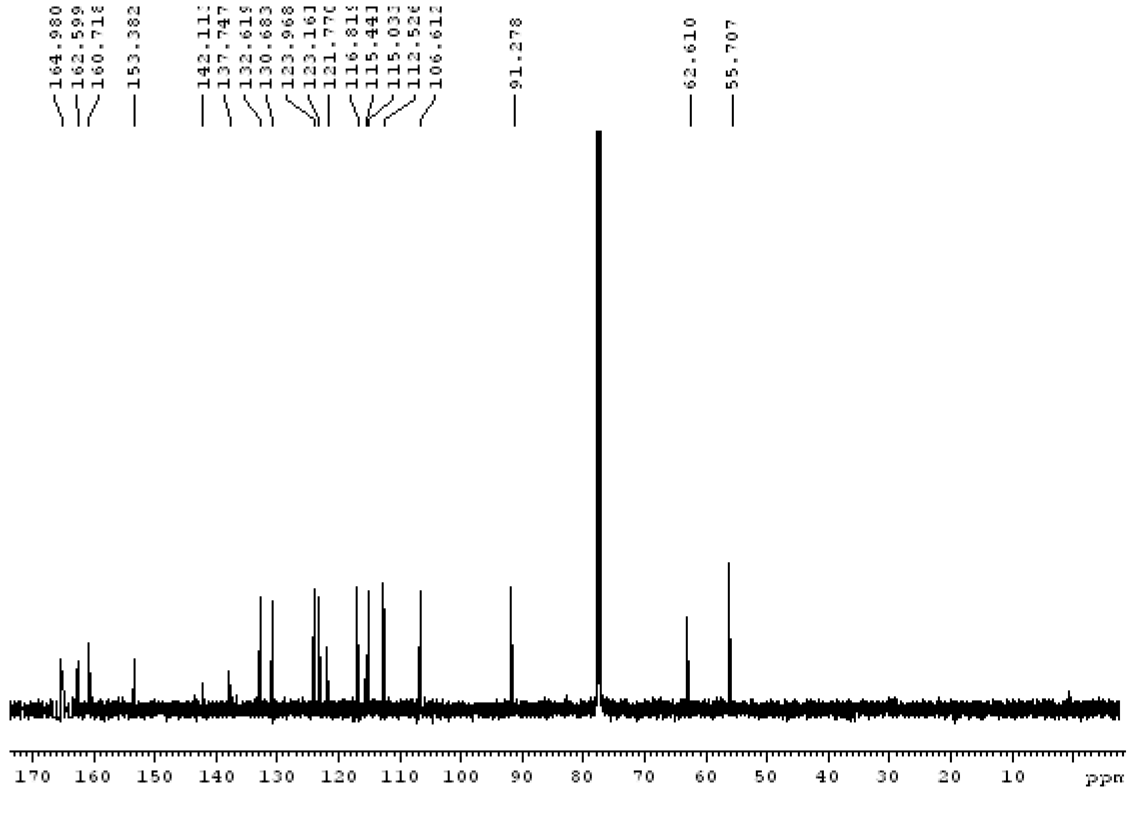

^{1}H-NMR ($CDCl_3$, 500 MHz) of compound 8d.

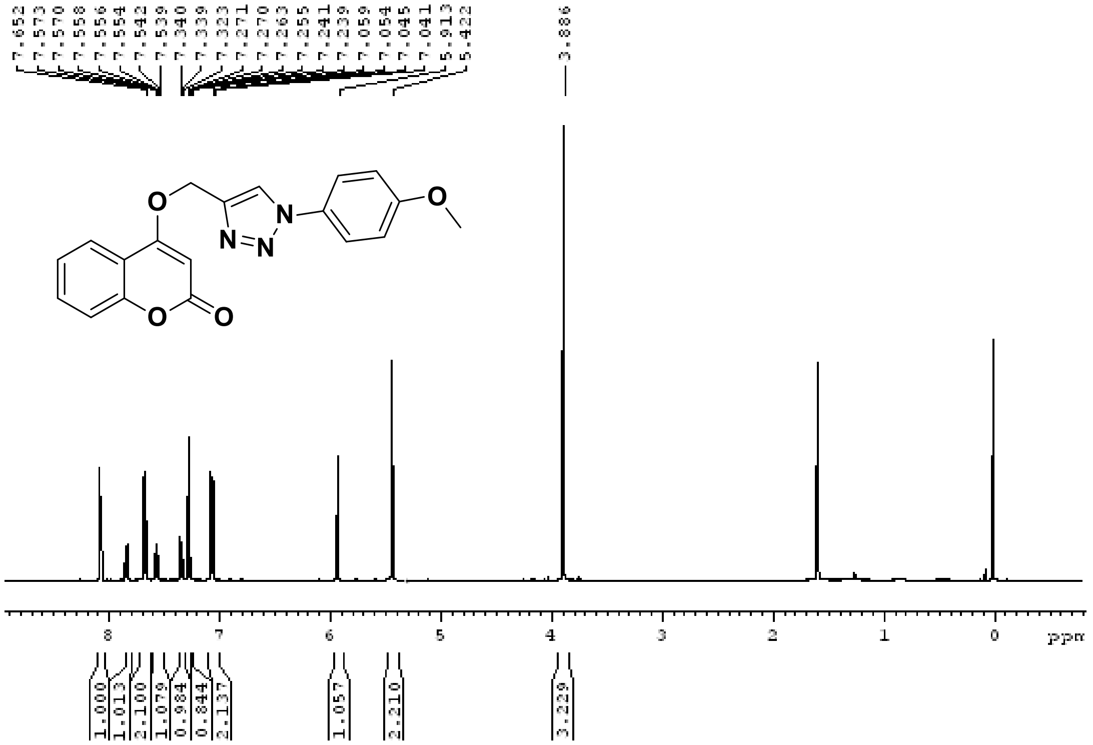

^{13}C-NMR ($CDCl_3$, 125 MHz) of compound 8d.

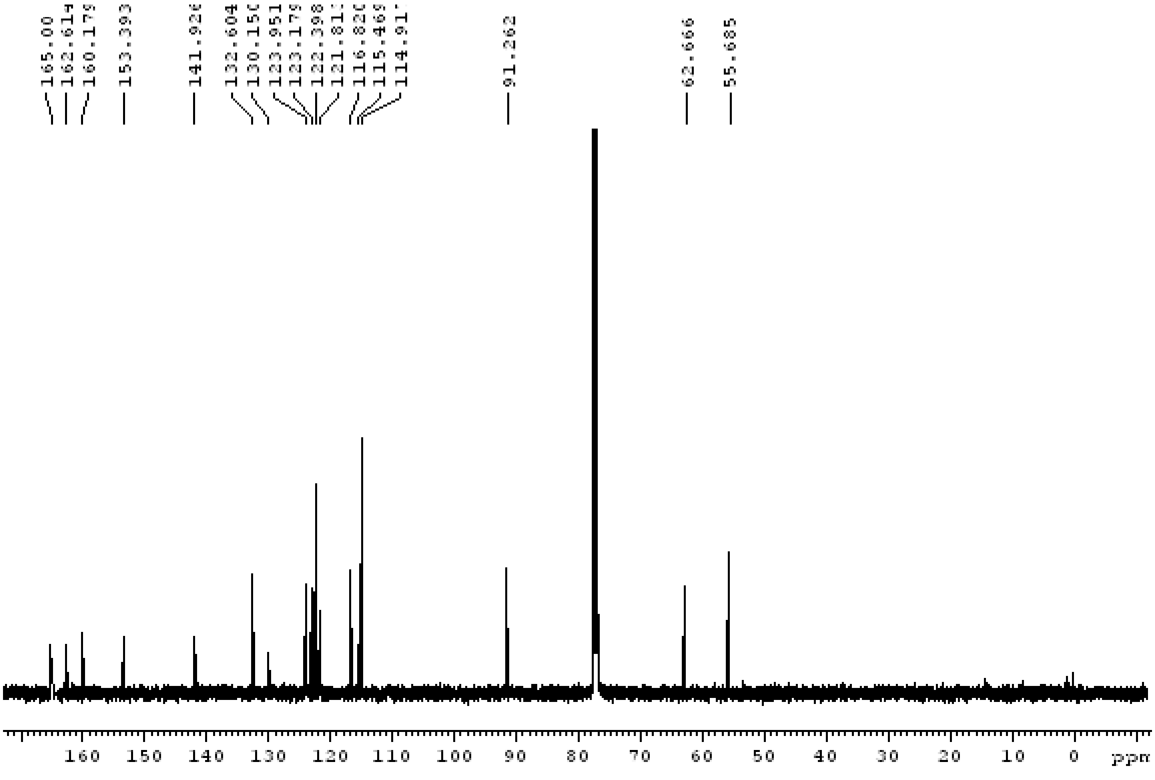

^{1}H-NMR (CDCl$_3$, 600 MHz) of compound 8e.

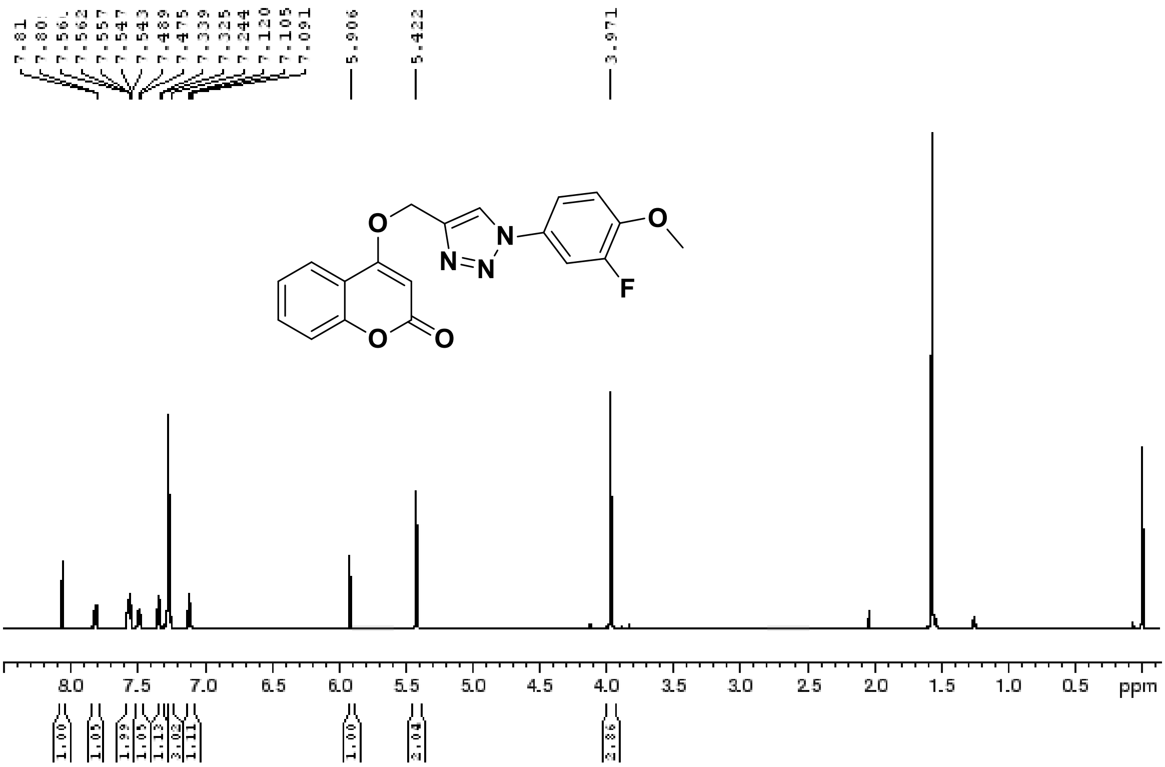

^{13}C-NMR (CDCl$_3$, 150 MHz) of compound 8e.

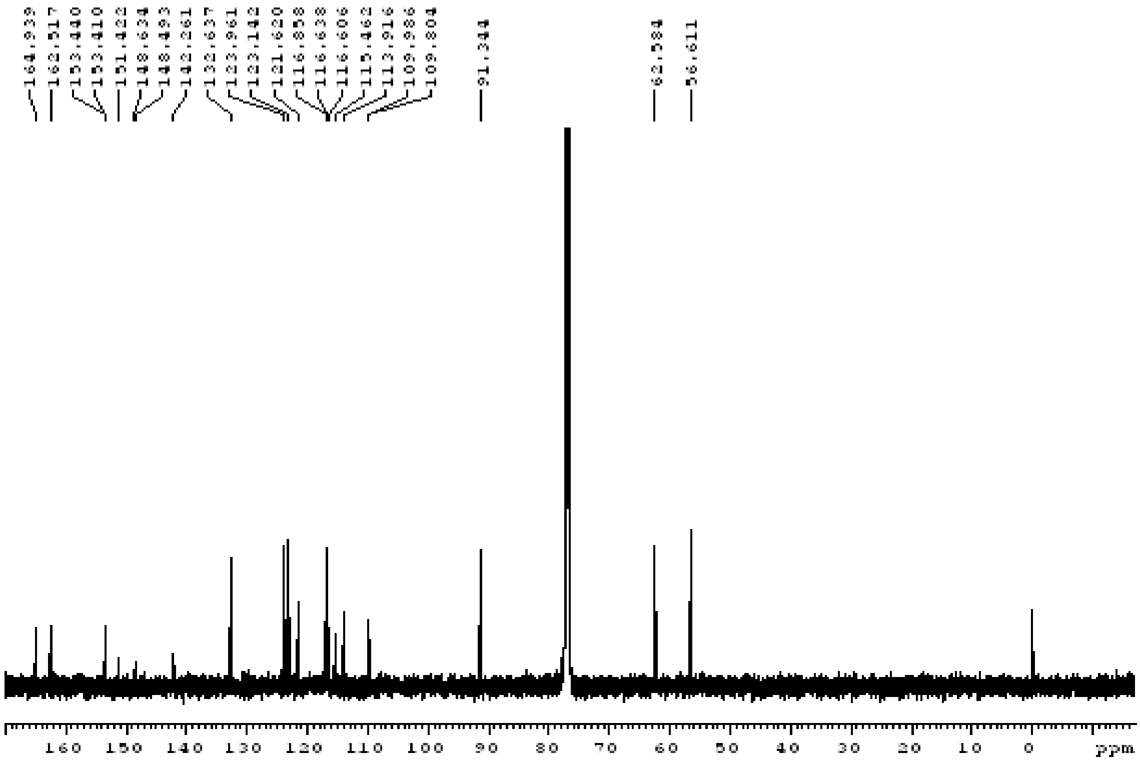

^{1}H-NMR ((CD$_3$)$_2$SO, 500 MHz) of compound 8f.

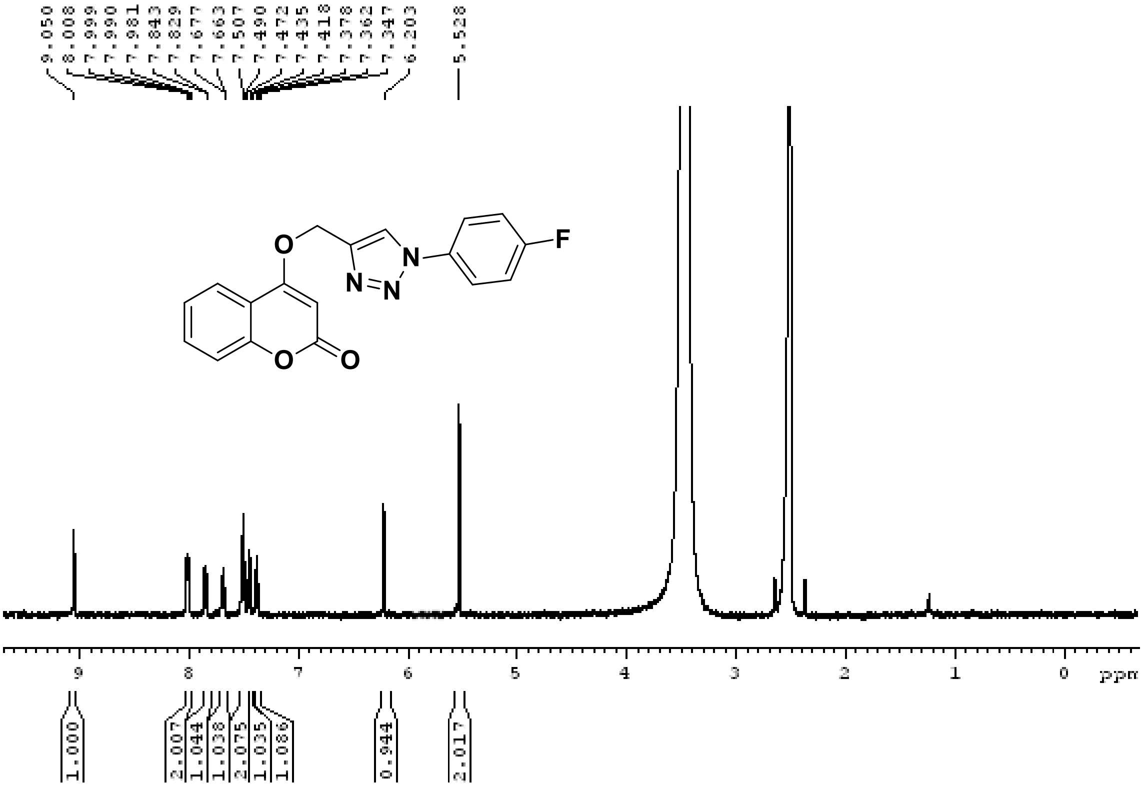

^{13}C-NMR ((CD$_3$)$_2$SO, 125 MHz) of compound 8f

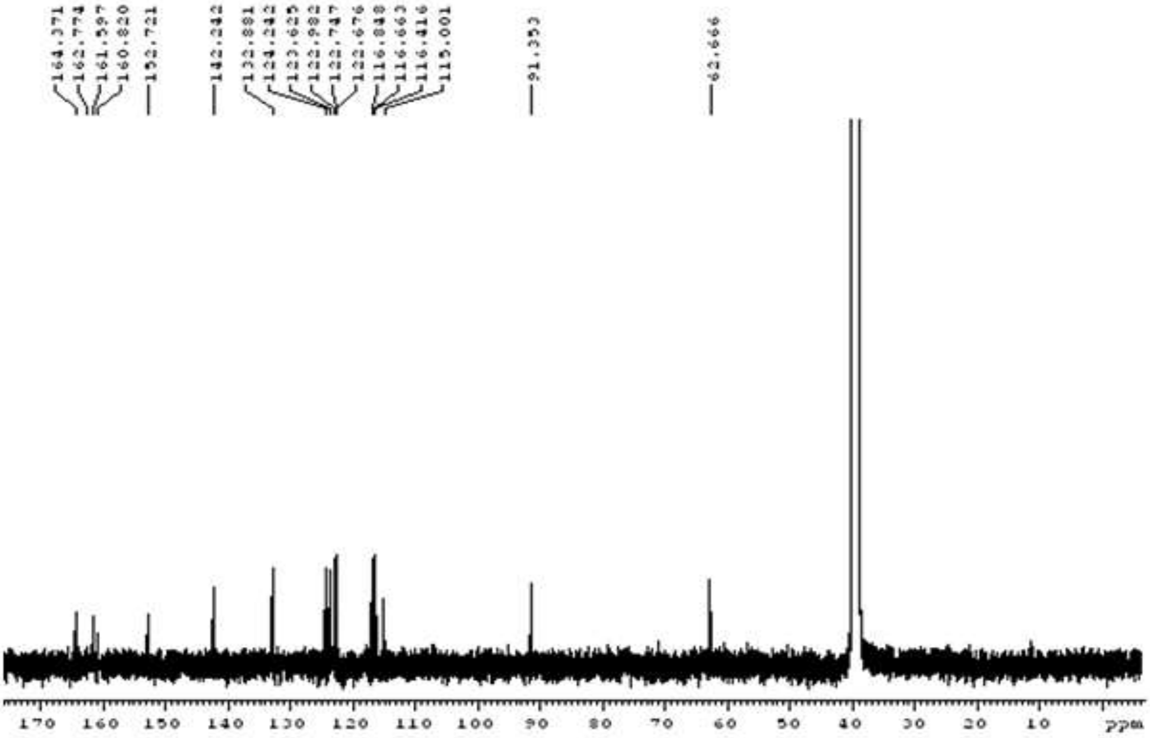

^{1}H-NMR (($(CD_3)_2SO$, 500 MHz) of compound 8g.

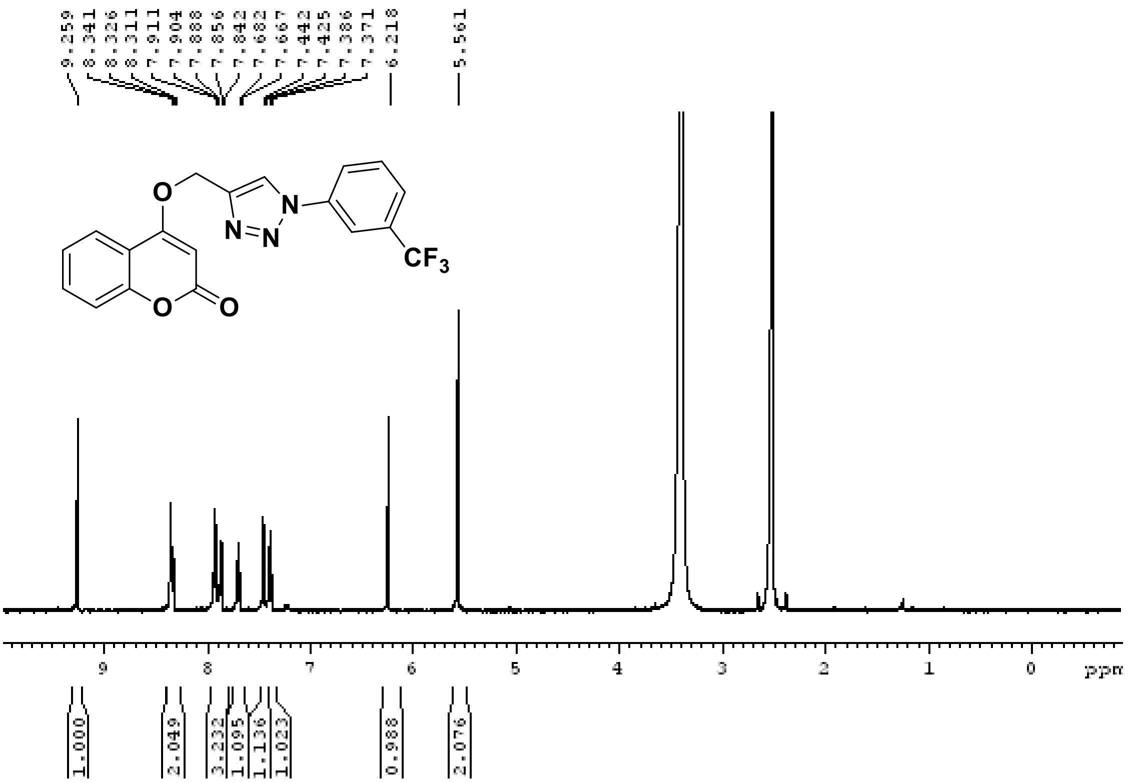

^{13}C-NMR (($(CD_3)_2SO$, 125 MHz) of compound 8g.

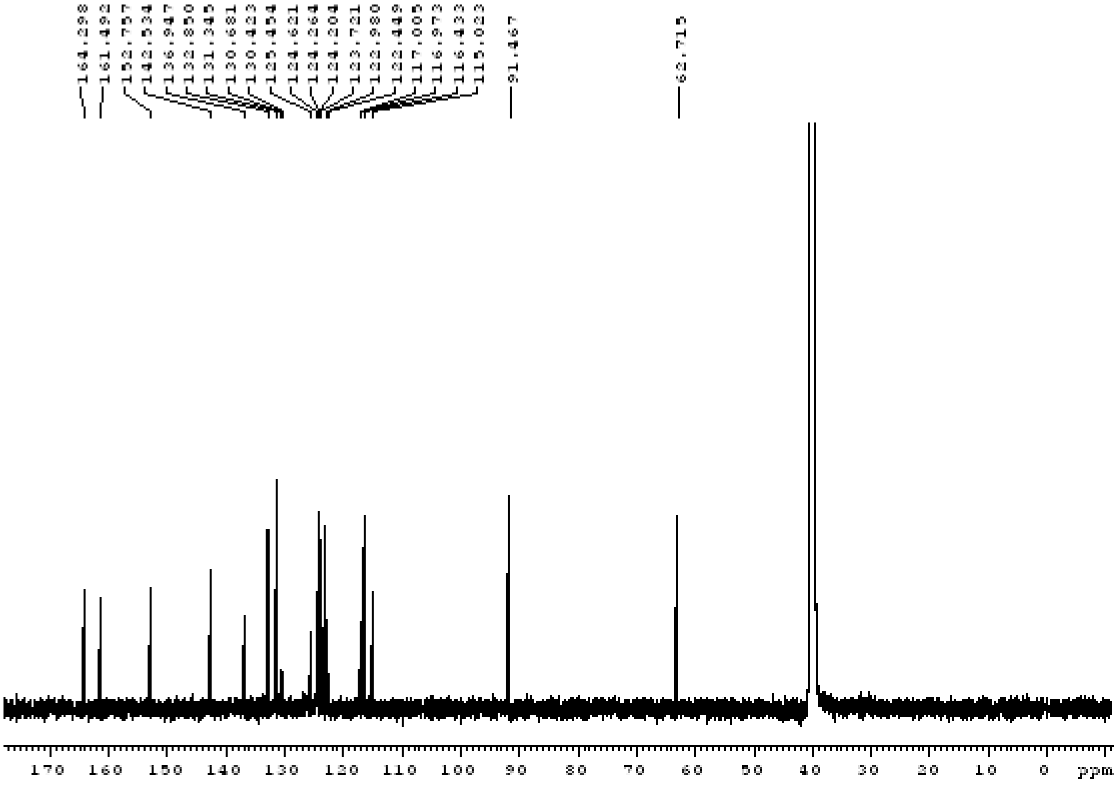

^{1}H-NMR (($(CD_3)_2SO$, 600 MHz) of compound 8h.

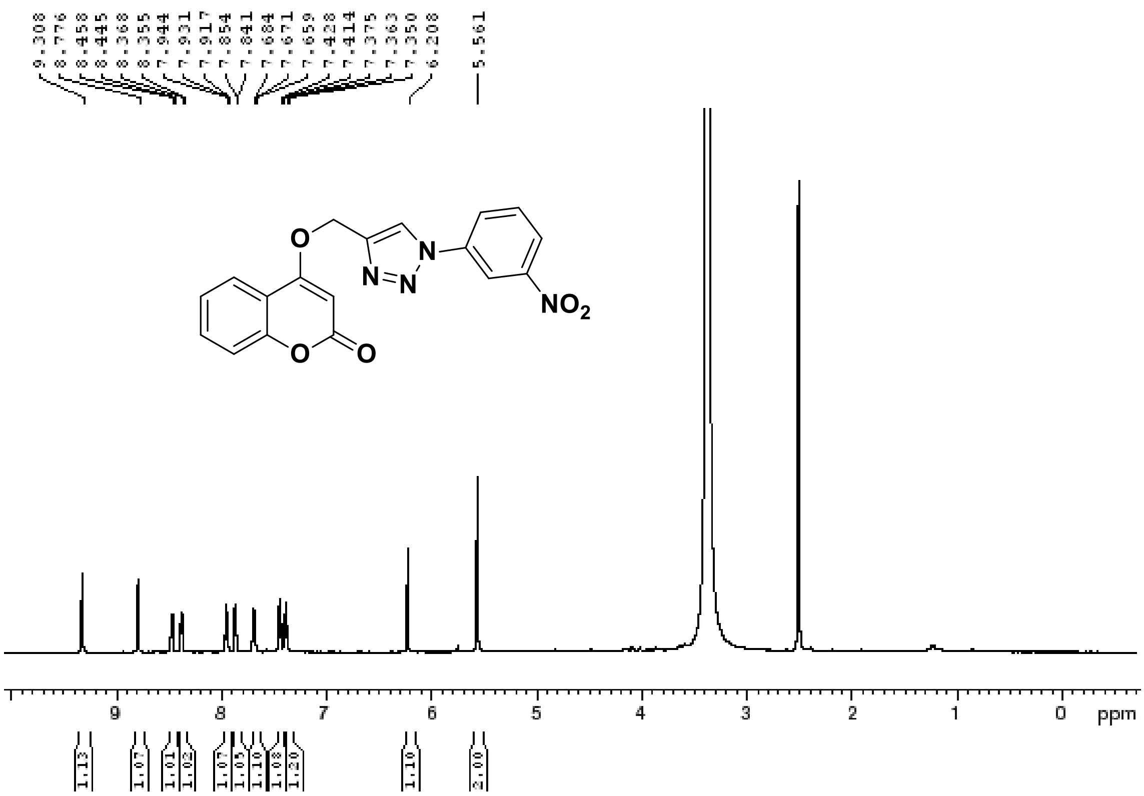

^{13}C-NMR (($(CD_3)_2SO$, 150 MHz) of compound 8h.

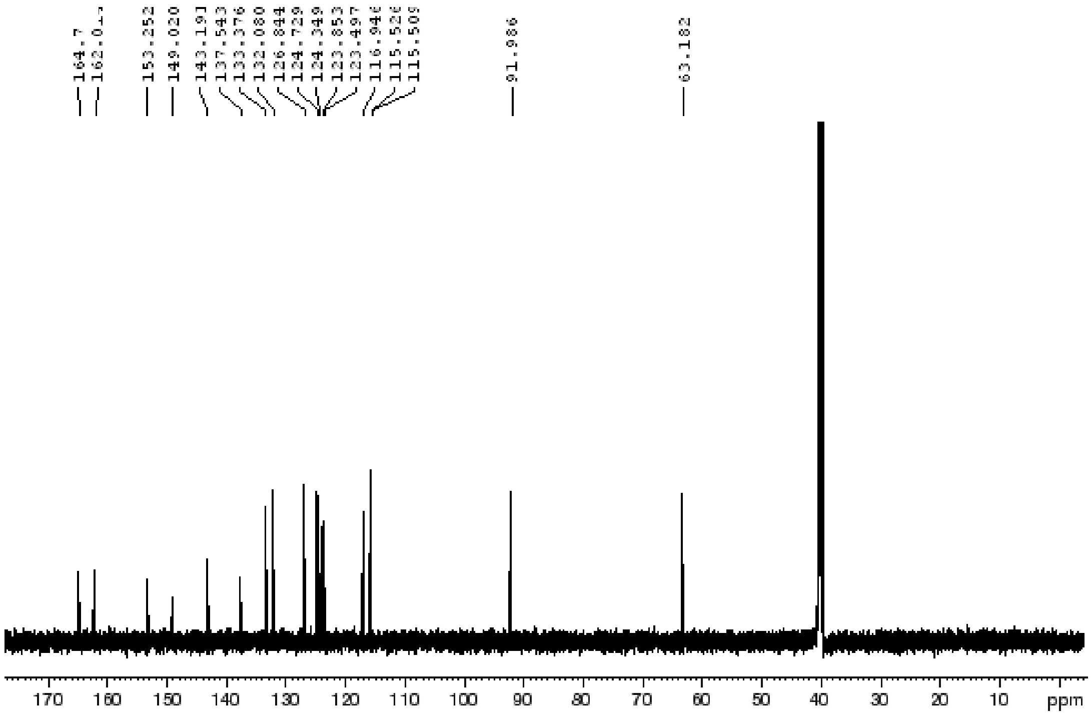

^{1}H-NMR ((CD$_3$)$_2$SO, 500 MHz) of compound 8i.

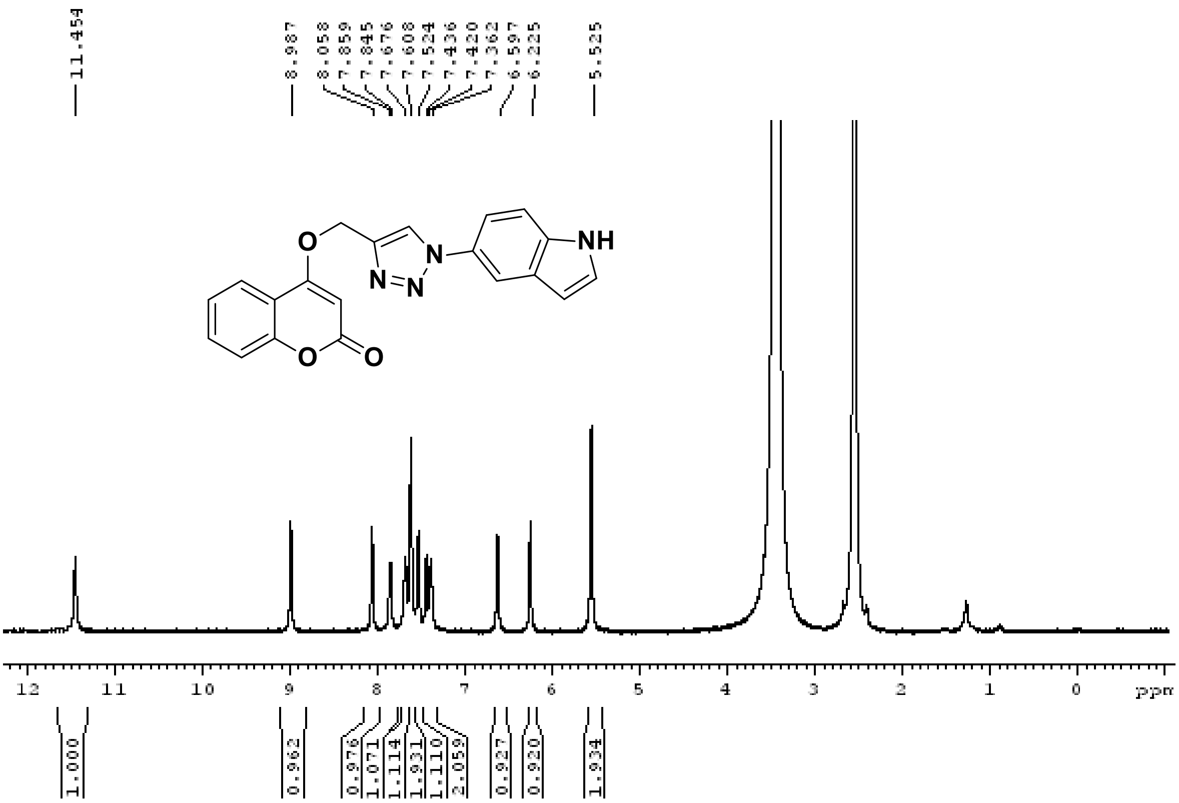

^{13}C-NMR ((CD$_3$)$_2$SO, 125 MHz) of compound 8i.

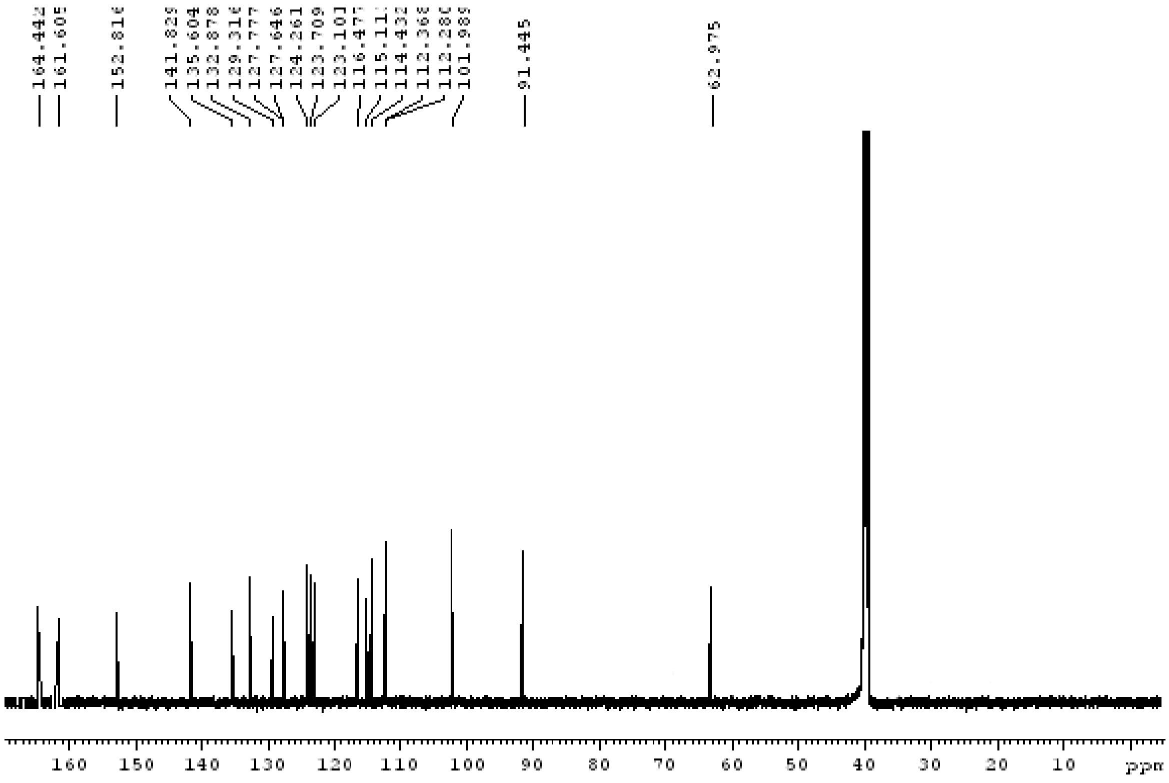

^{1}H-NMR (($(CD_3)_2$SO, 500 MHz) del compuesto8j.

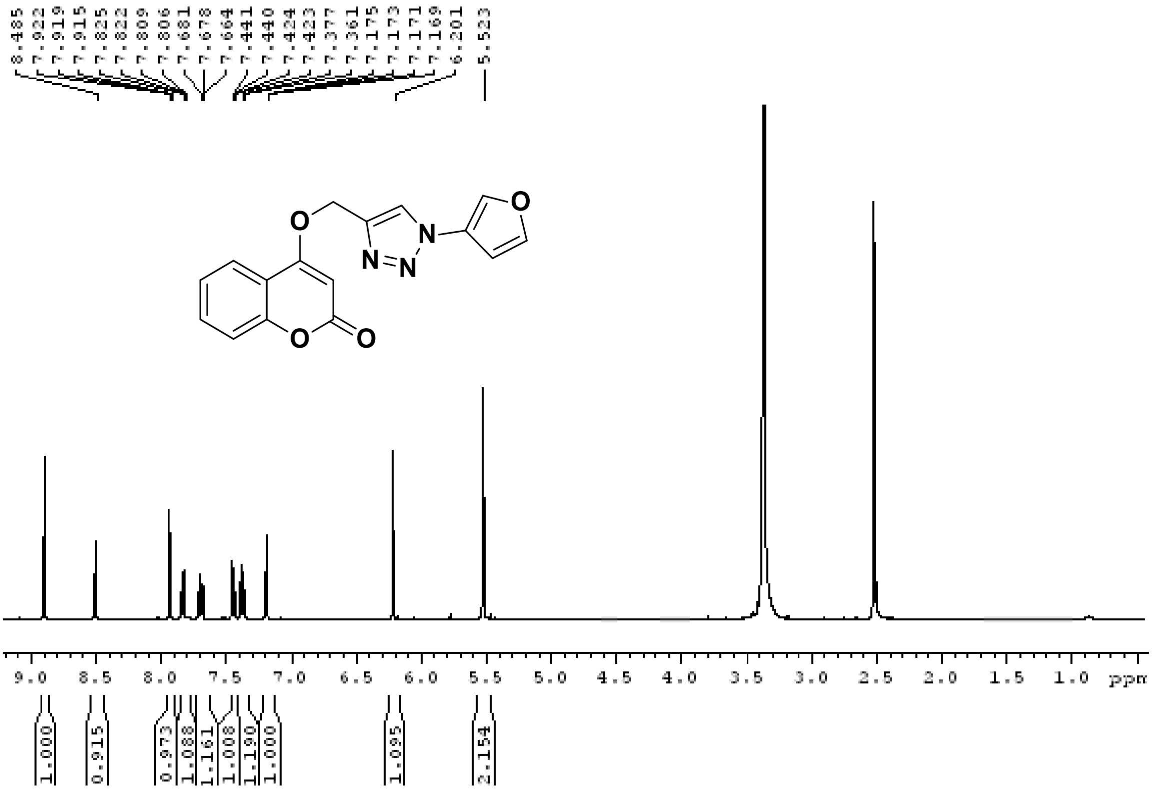

^{13}C-NMR (($(CD_3)_2$SO, 125 MHz) of compound 8j.

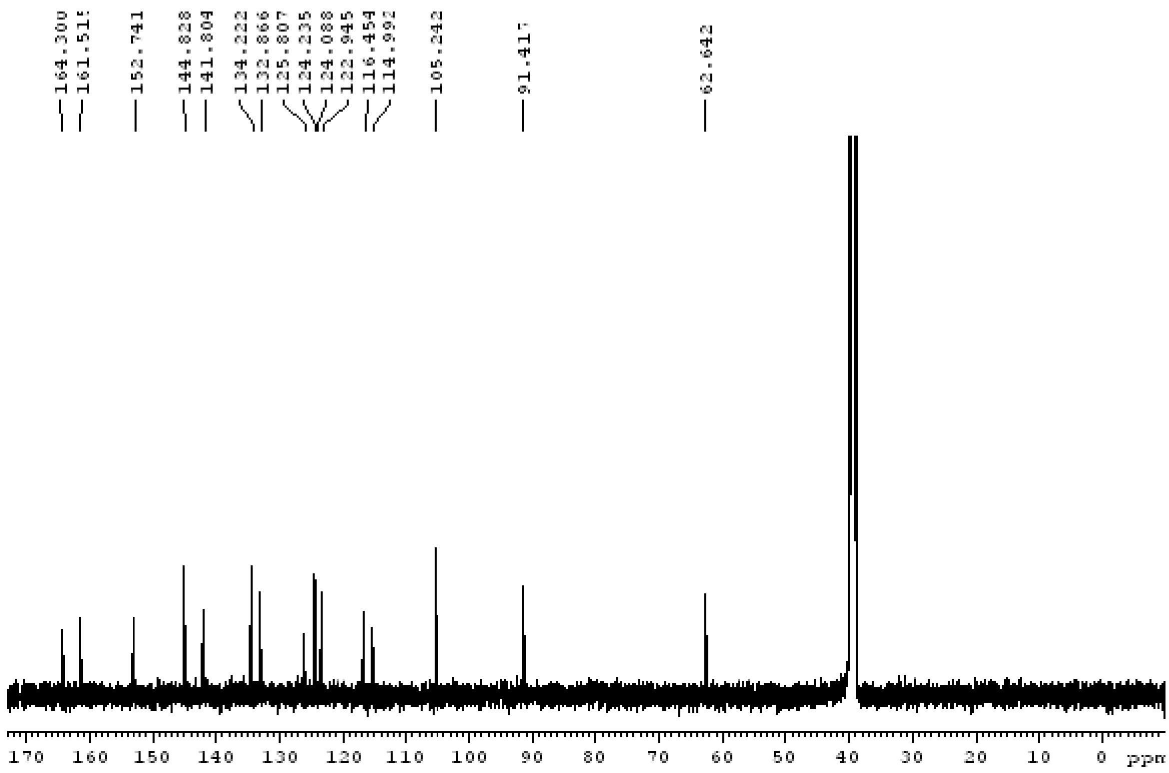

^{1}H-NMR ($CDCl_3$, 500 MHz) of compound 8k.

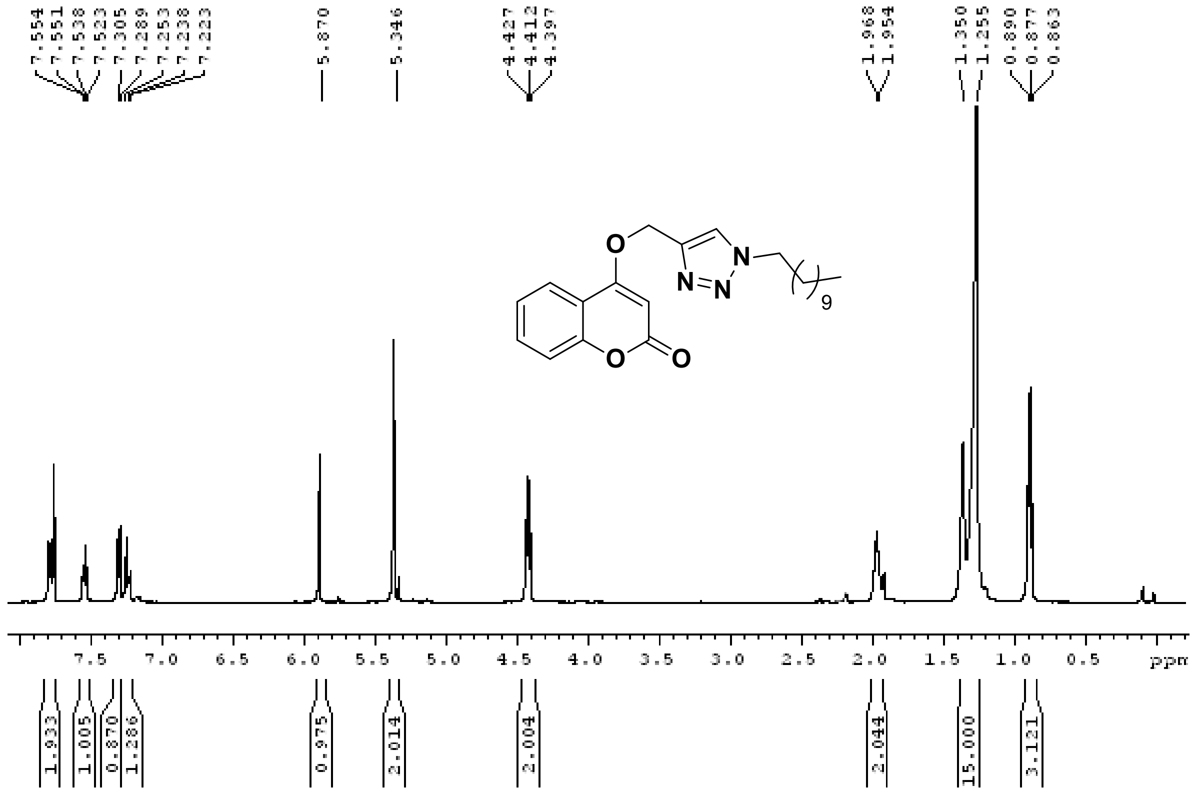

^{13}C-NMR ($CDCl_3$, 125 MHz) of compound 8k.

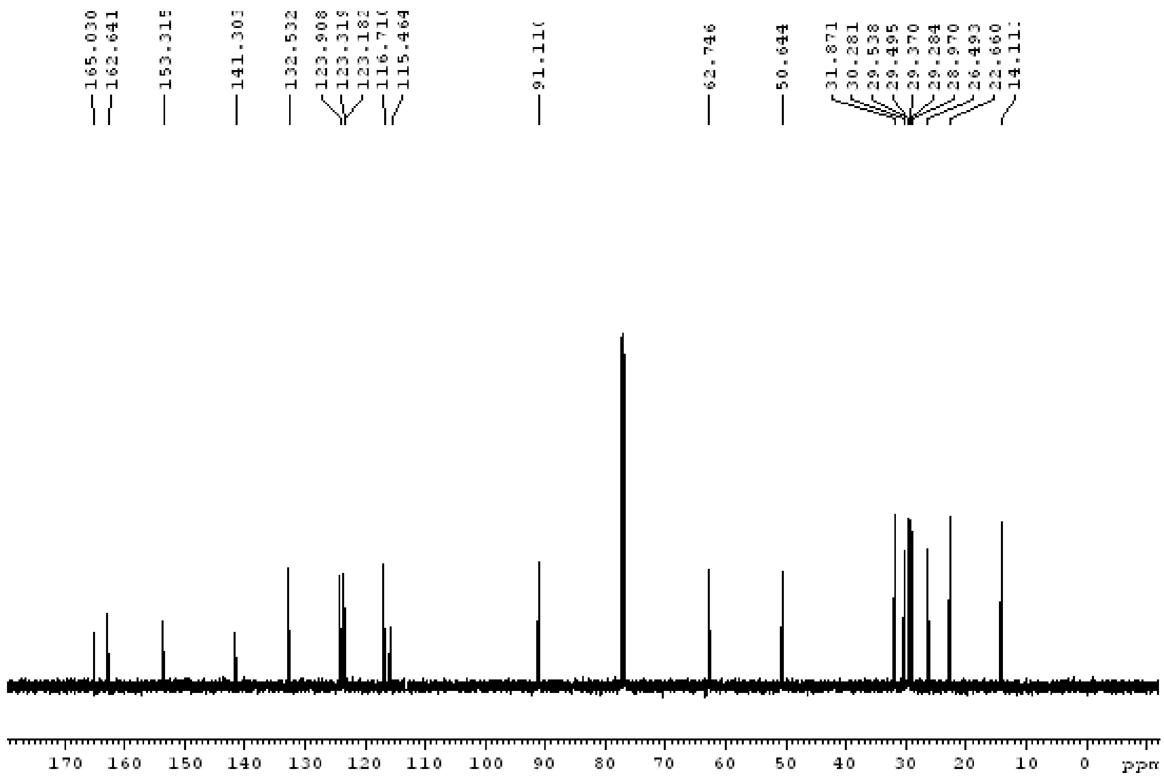

^{1}H-NMR ($CDCl_3$, 500 MHz) of compound 8l.

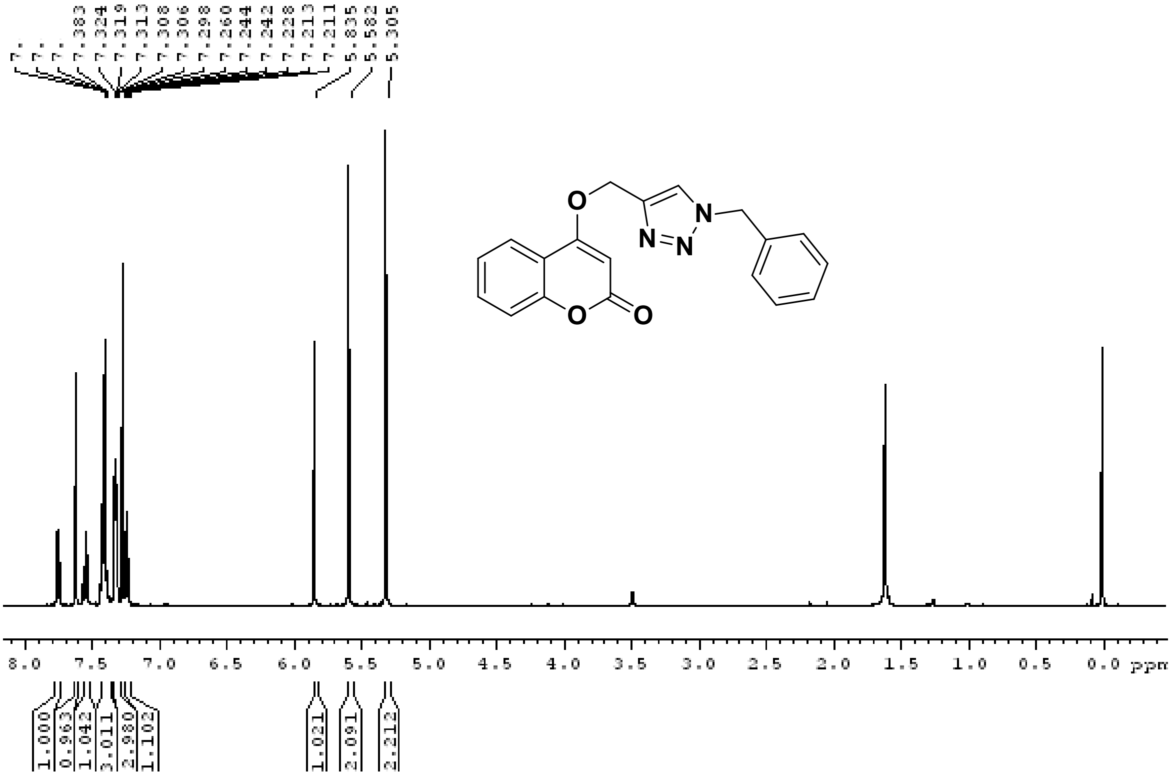

^{13}C-NMR ($CDCl_3$, 125 MHz) of compound 8l.

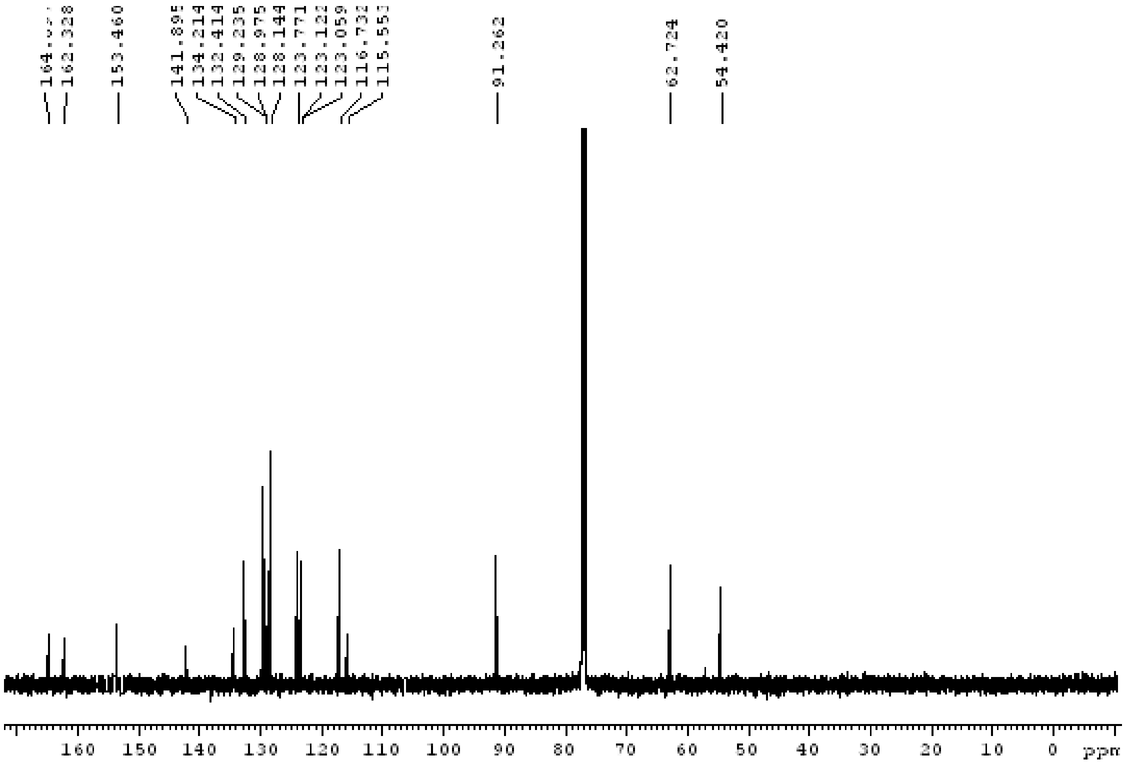

^{1}H-NMR ($CDCl_3$, 500 MHz) of compound 8m.

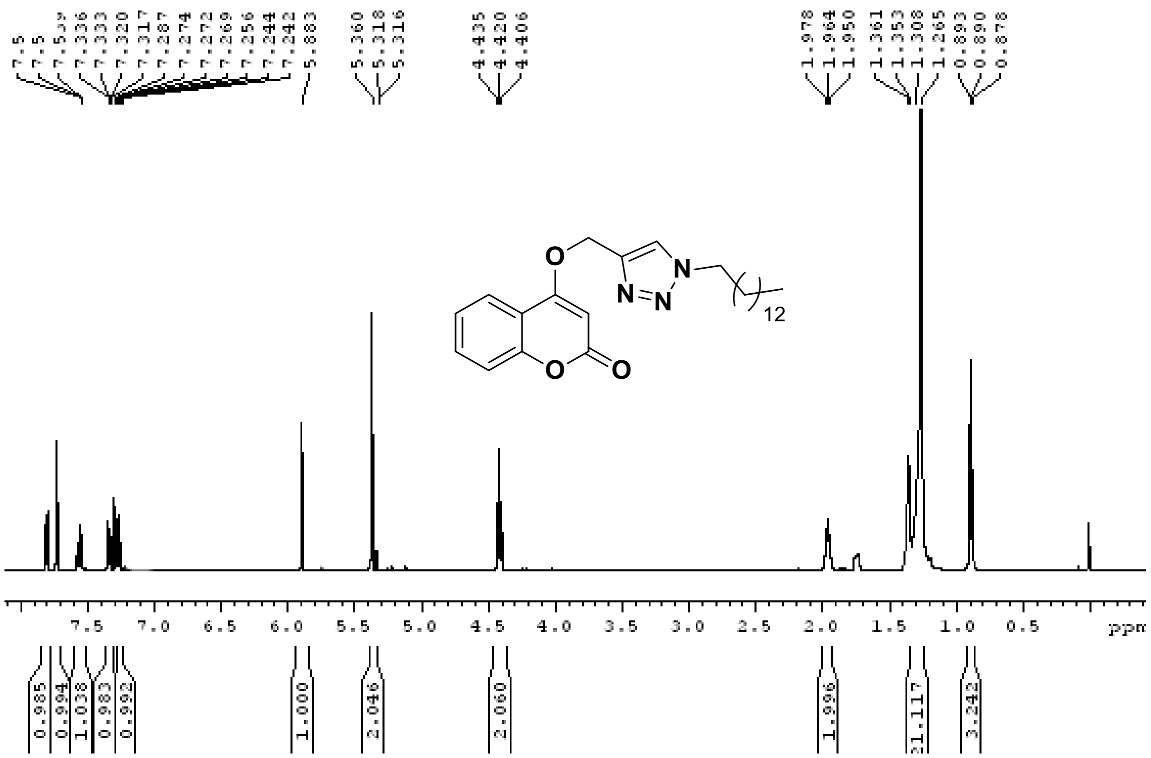

^{13}C-NMR (CDCl$_3$, 125 MHz) of compound 8m.

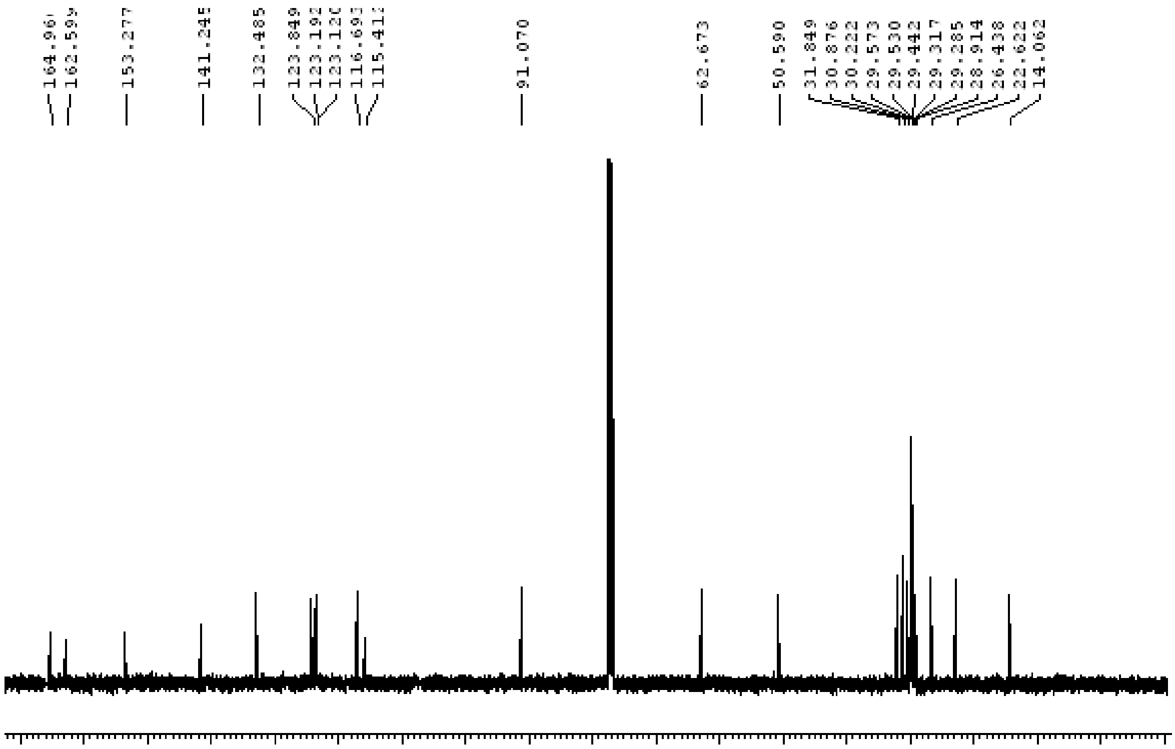

^{1}H-NMR ((CD$_3$)$_2$SO, 500 MHz) of compound 9a.

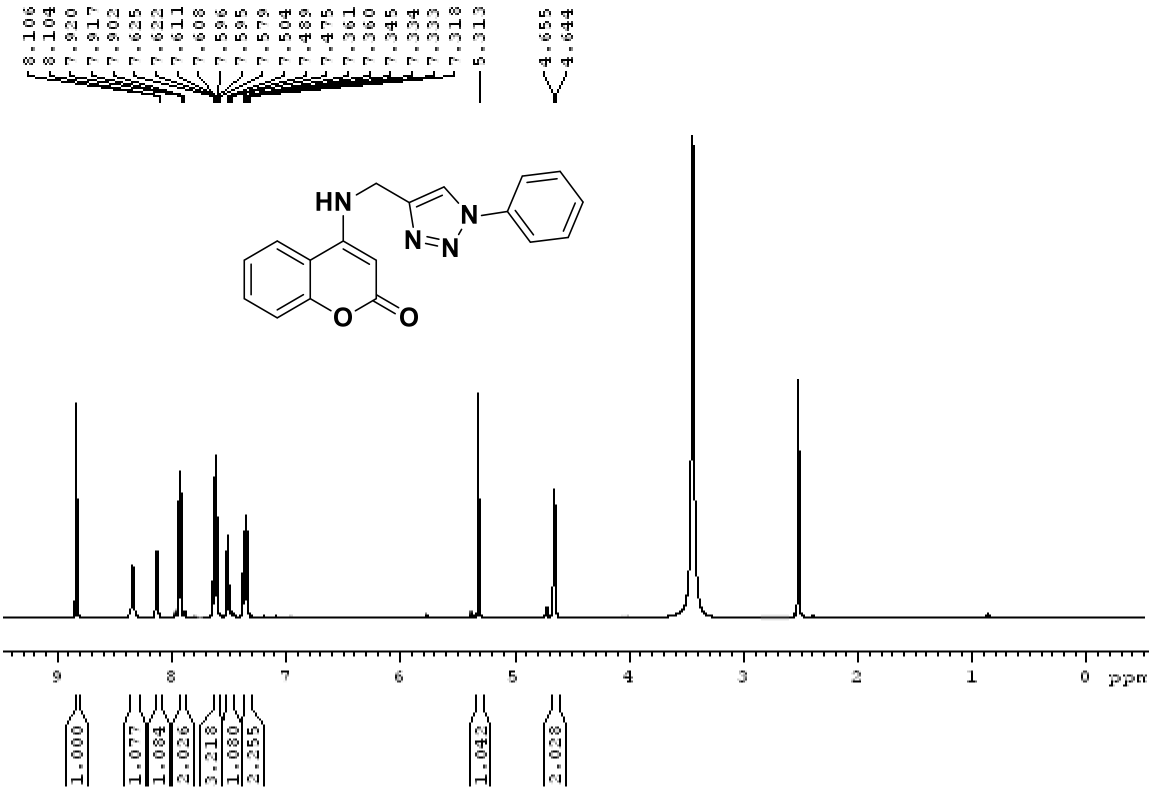

^{13}C-NMR ((CD3)2SO, 125 MHz) of compound 9a.

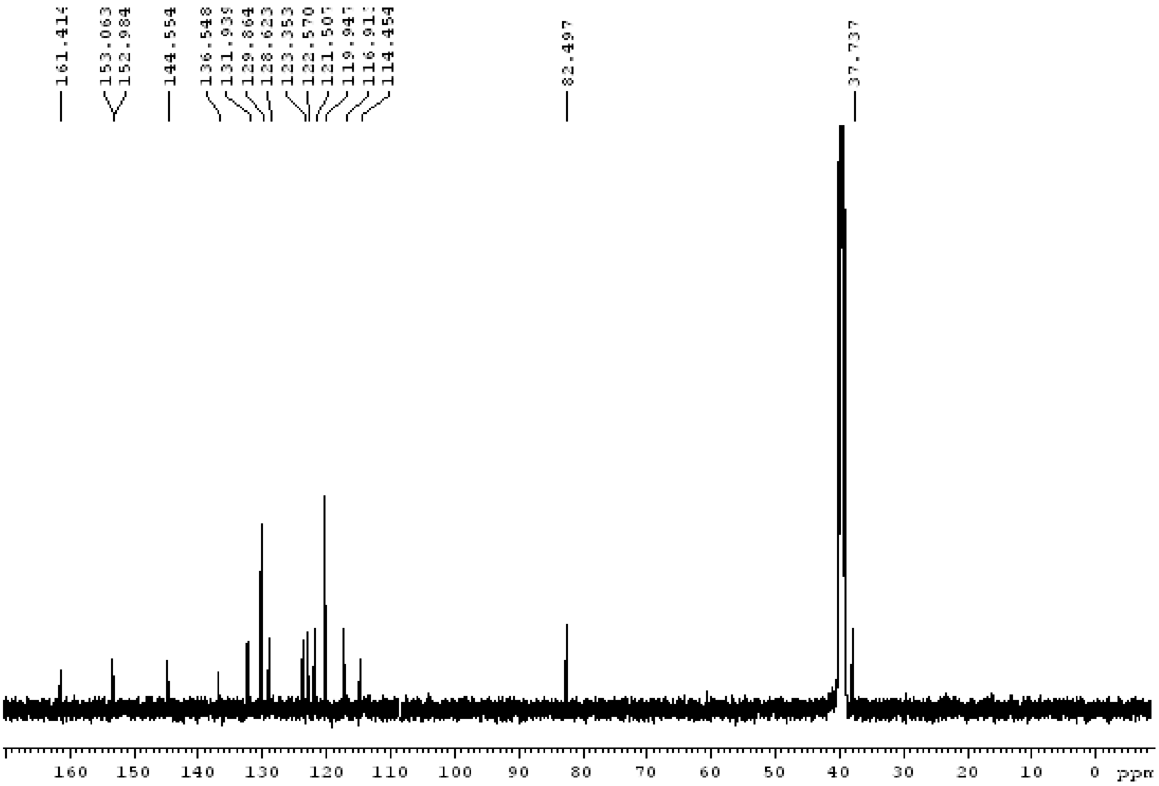

^{1}H-NMR ((CD3)2SO, 500 MHz) of compound 9b.

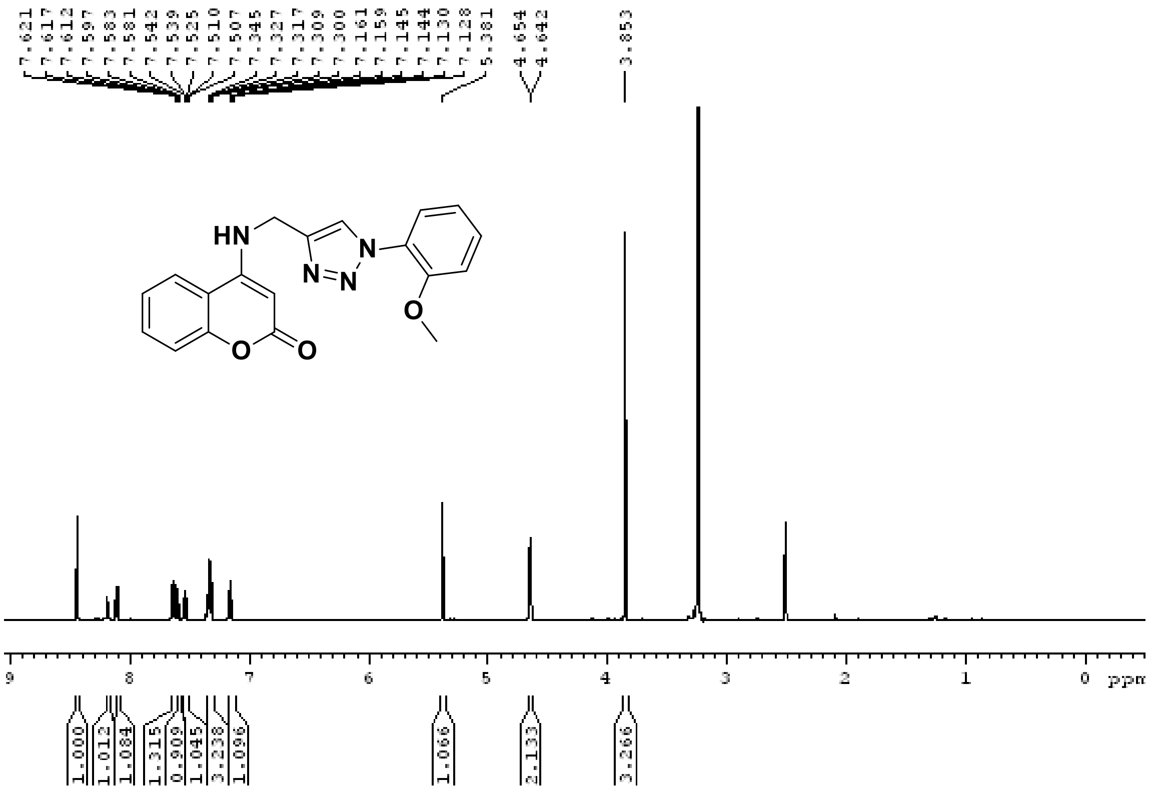

^{13}C-NMR ((CD3)2SO, 125 MHz) of compound 9b.

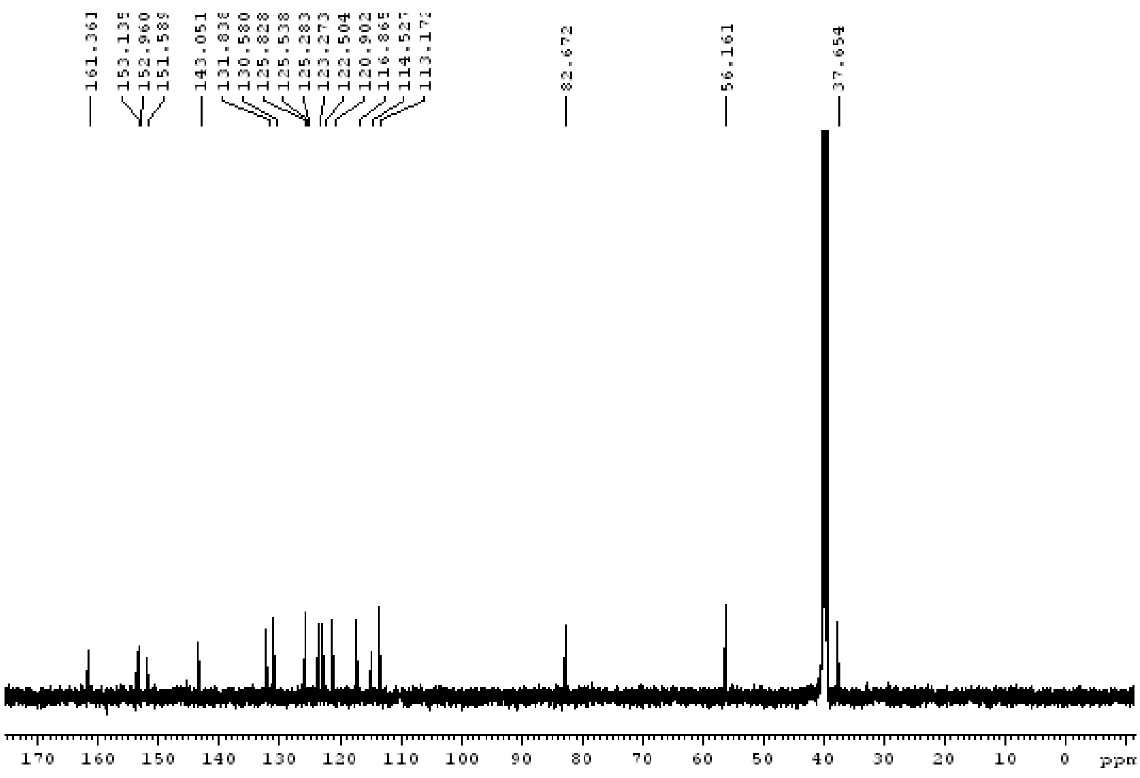

^{1}H-NMR ((CD3)2SO, 500 MHz) of compound 9c.

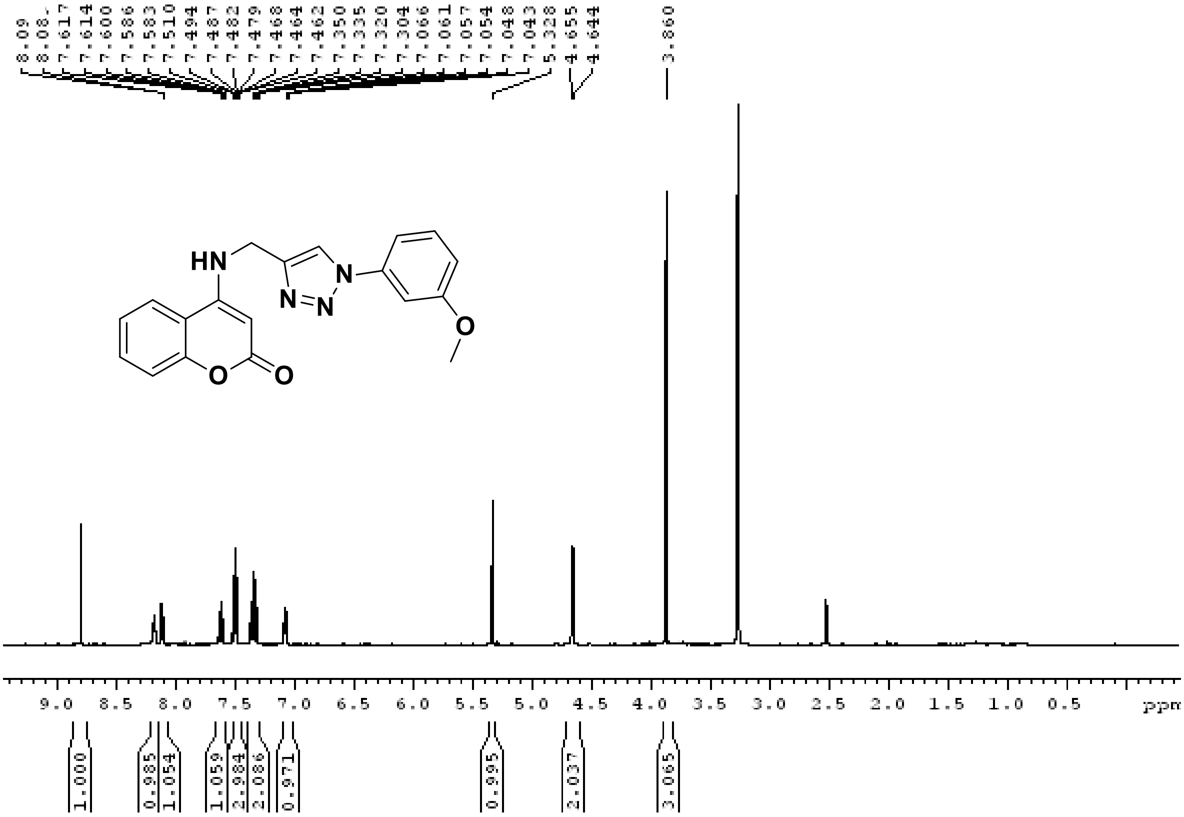

^{13}C-NMR ((CD_3)$_2$SO, 125 MHz) of compound 9c.

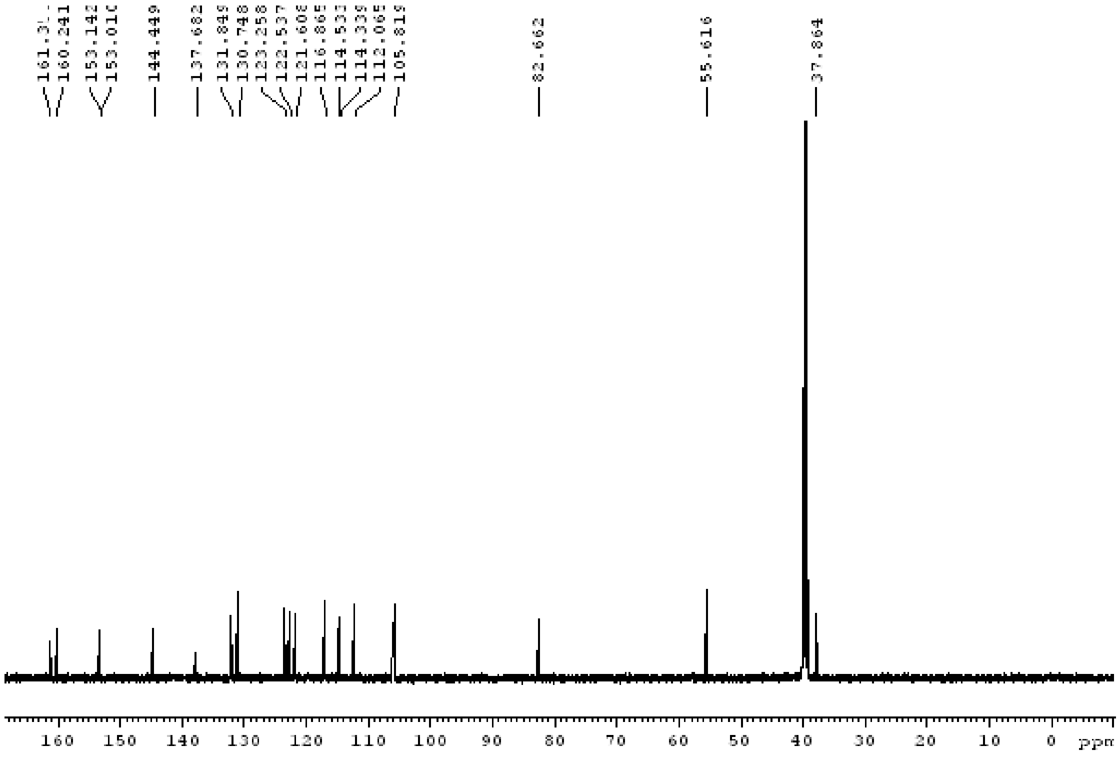

^{1}H-NMR ((CD_3)$_2$SO, 500 MHz) of compound 9d.

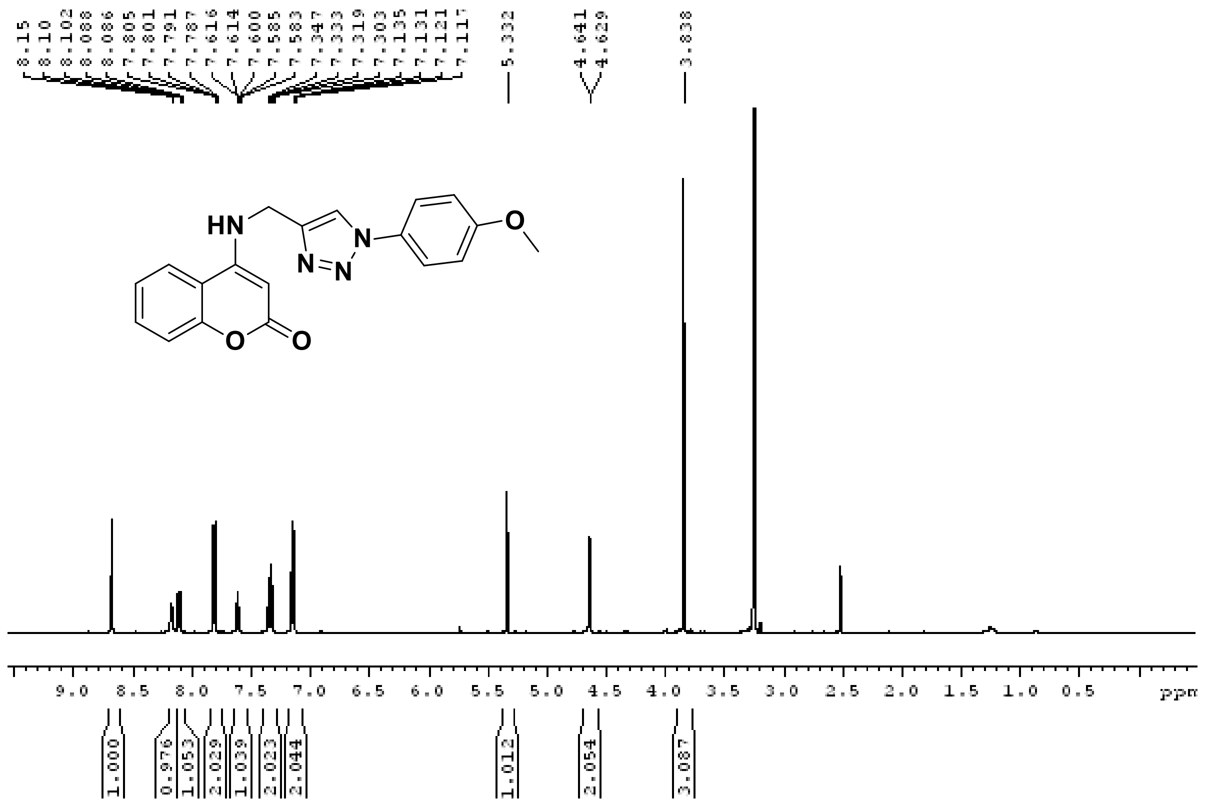

^{13}C-NMR ((CD3)2SO, 125 MHz) of compound 9d.

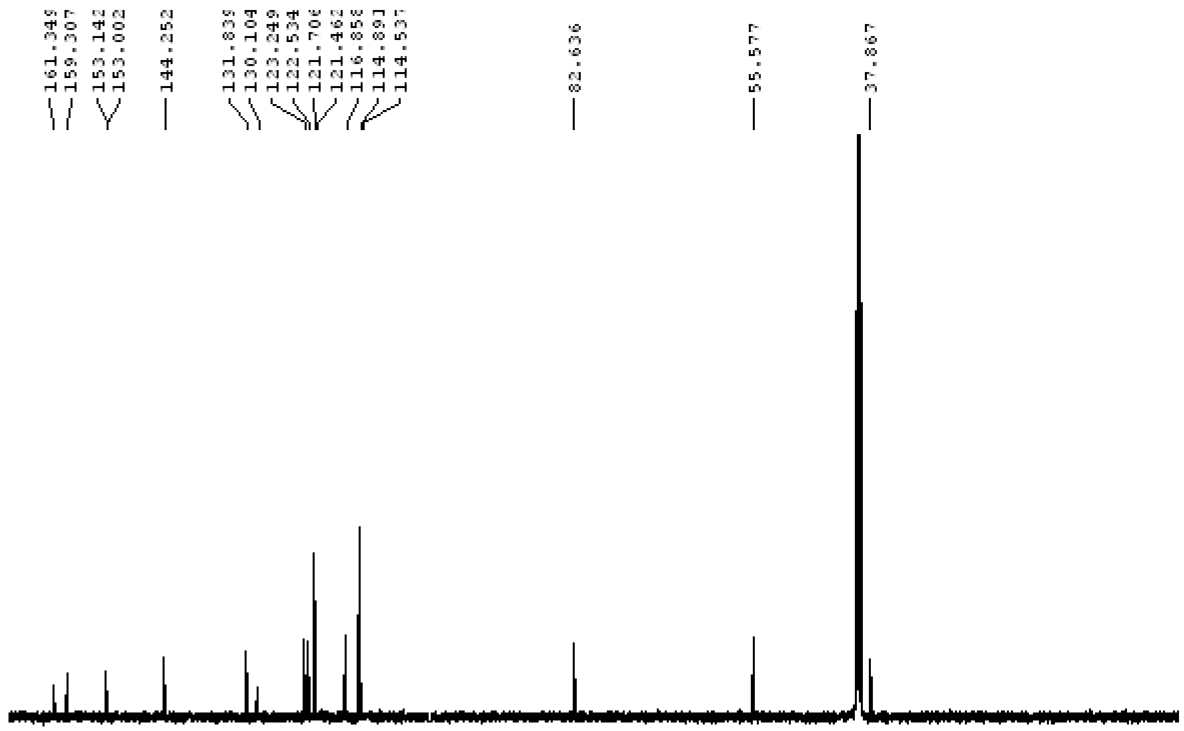

^{1}H-NMR (($(CD_3)_2SO$, 500 MHz) of compound 9e.

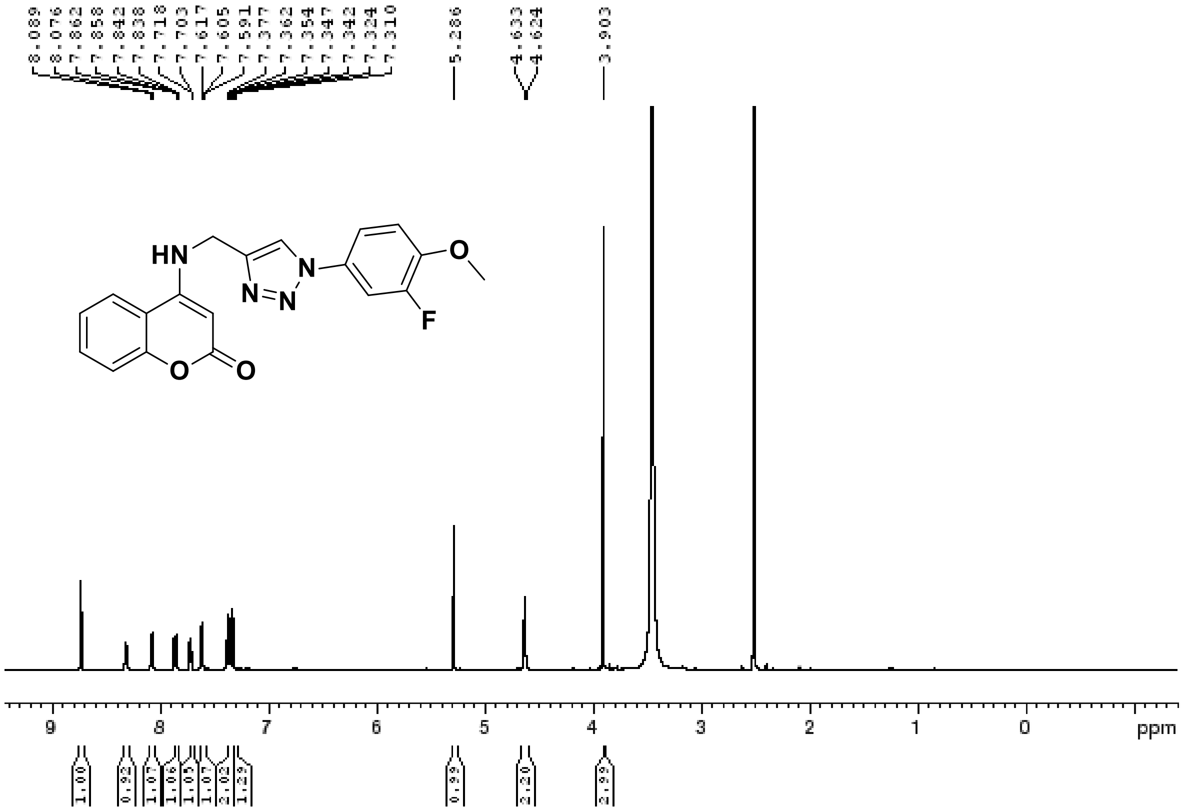

^{13}C-NMR (($(CD_3)_2SO$, 125 MHz) of compound 9e.

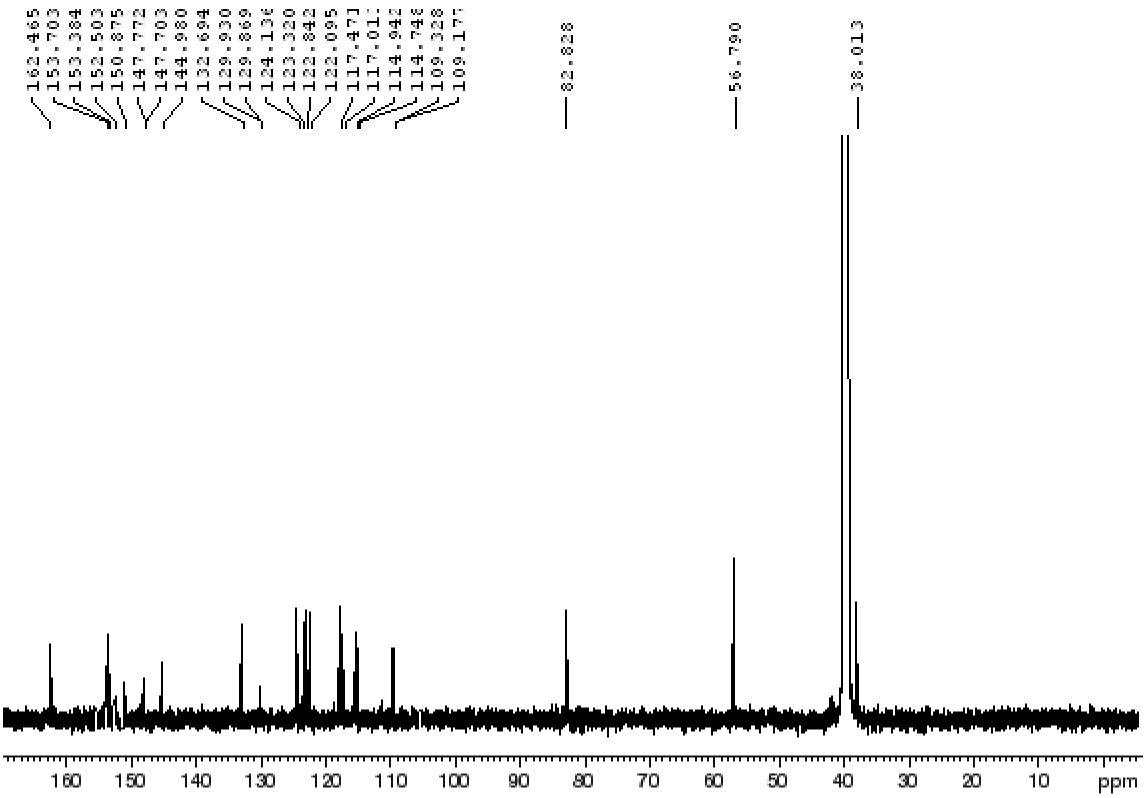

^{1}H-NMR ((CD$_3$)$_2$SO, 500 MHz) of compound 9f:

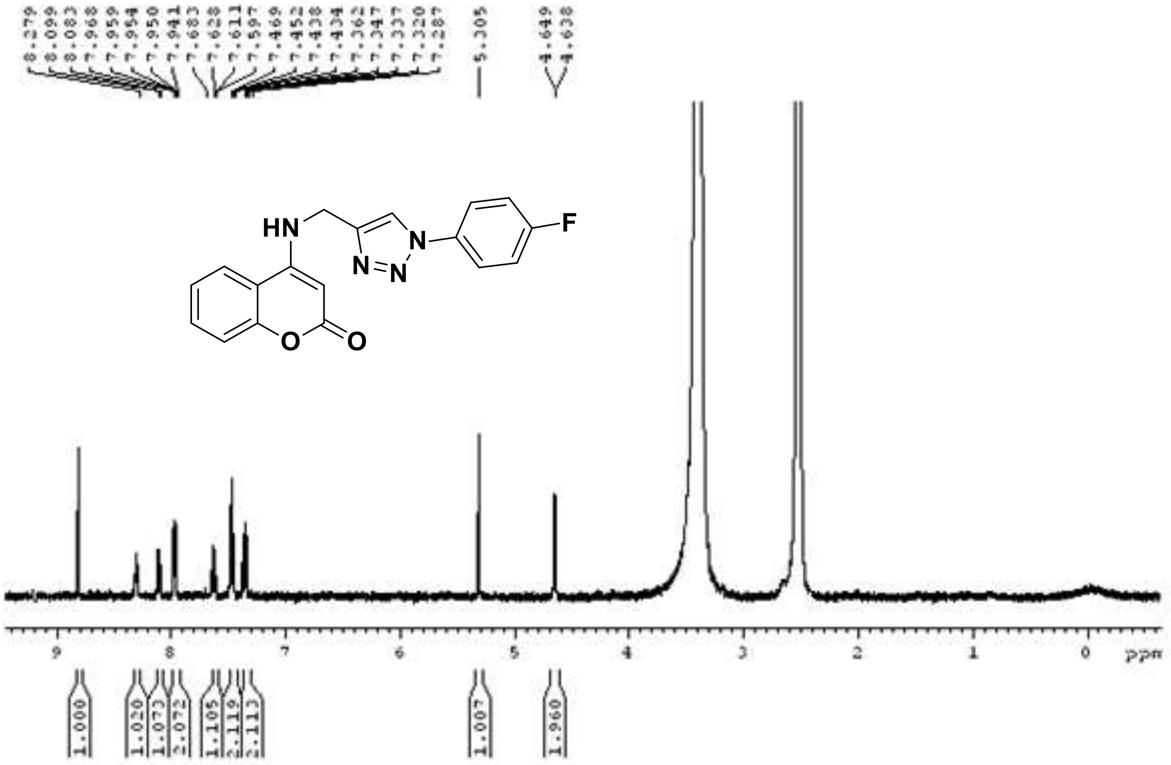

^{13}C-NMR ((CD$_3$)$_2$SO, 125 MHz) of compound 9f.

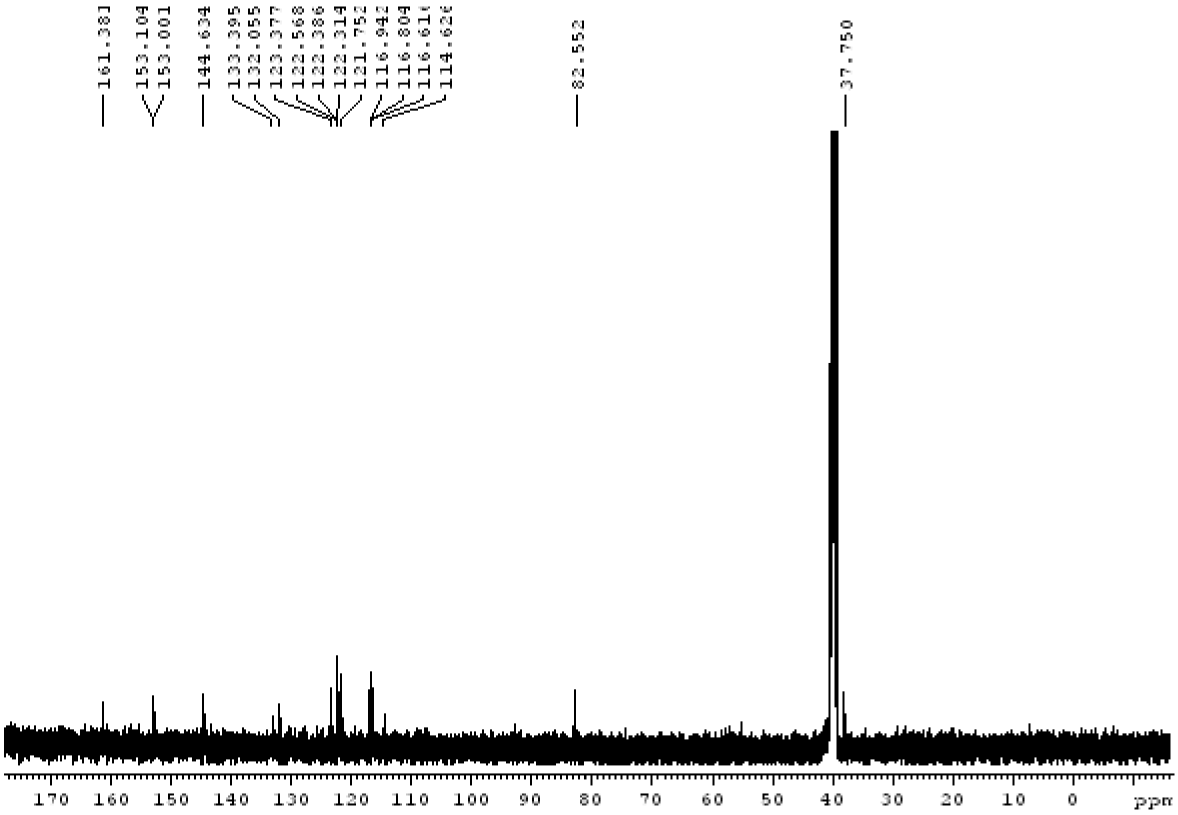

^{1}H-NMR ((CD3)2SO, 500 MHz) of compound 9g.

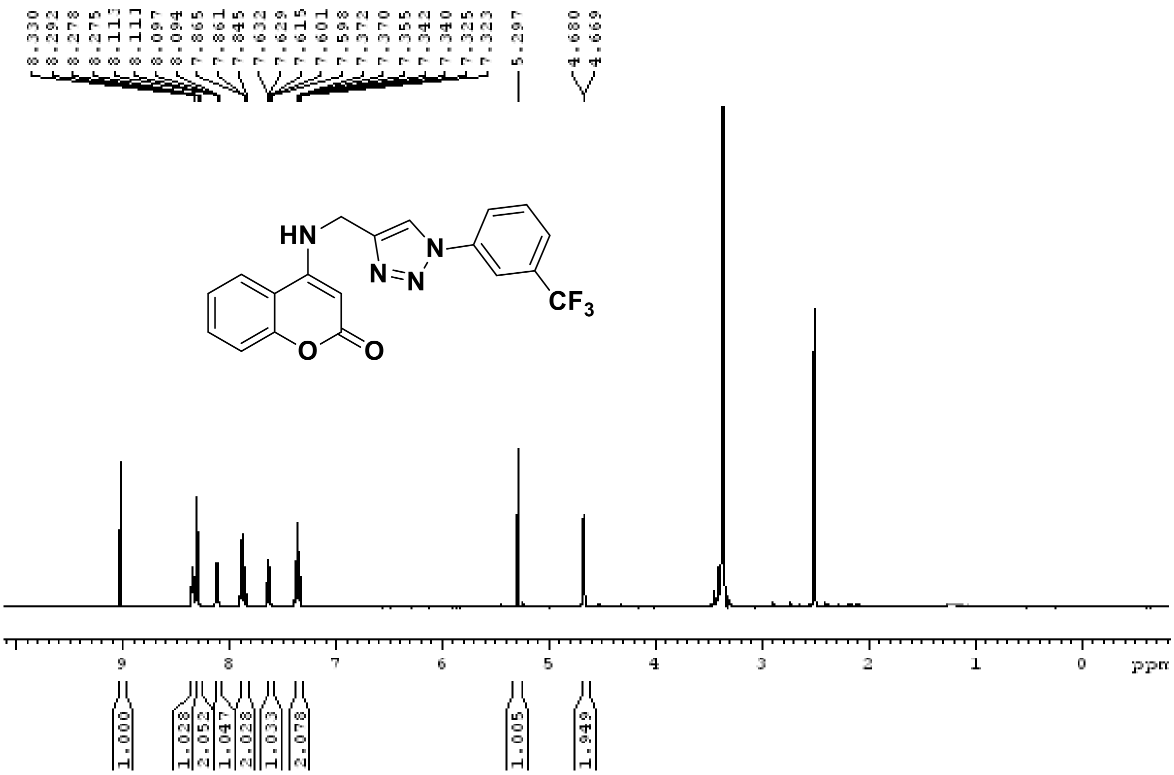

^{13}C-NMR ((CD3)2SO, 125 MHz) of compound 9g.

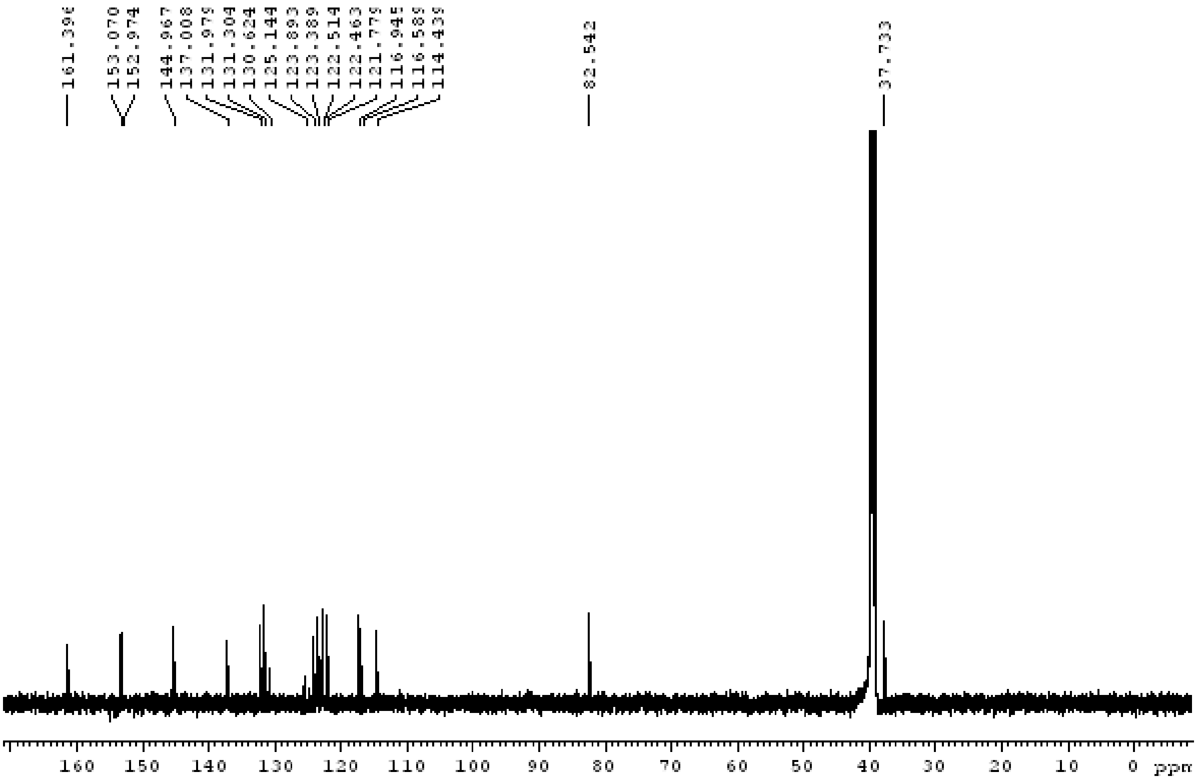

^{1}H-NMR ($(CD_3)_2SO$, 600 MHz) of compound 9h.

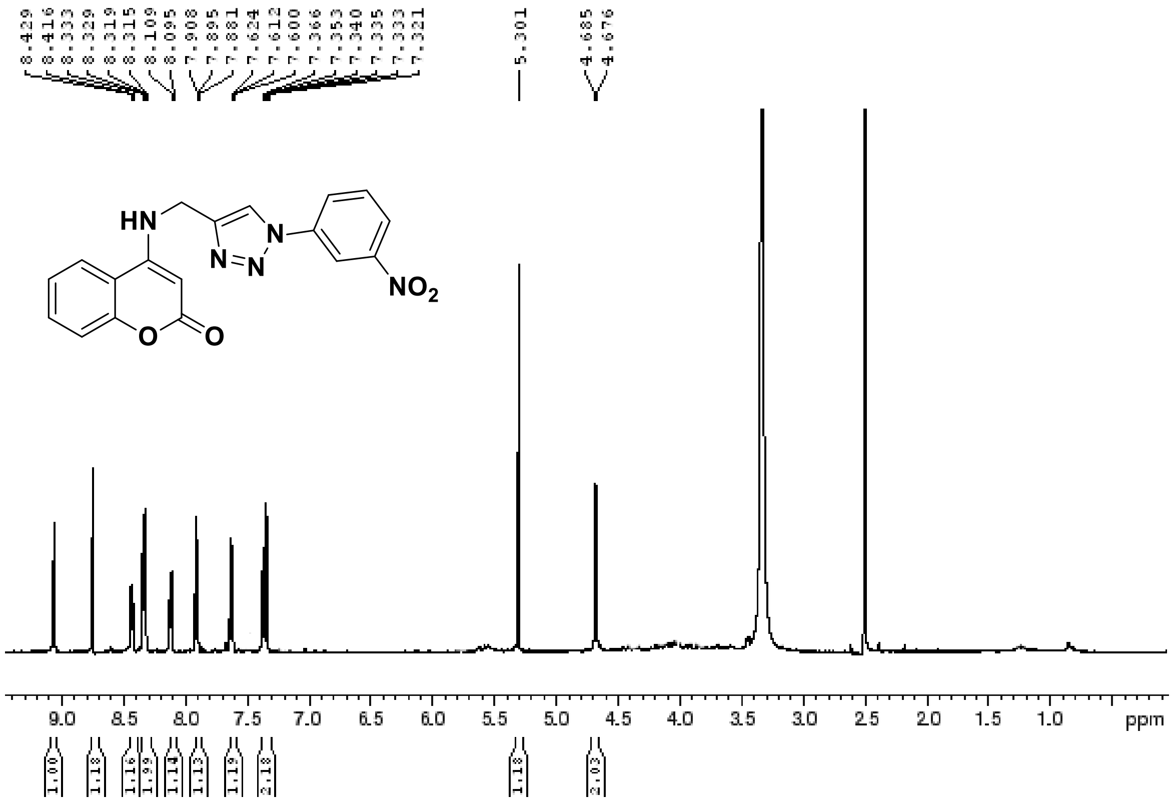

^{13}C-NMR ($(CD_3)_2SO$, 150 MHz) of compound 9h.

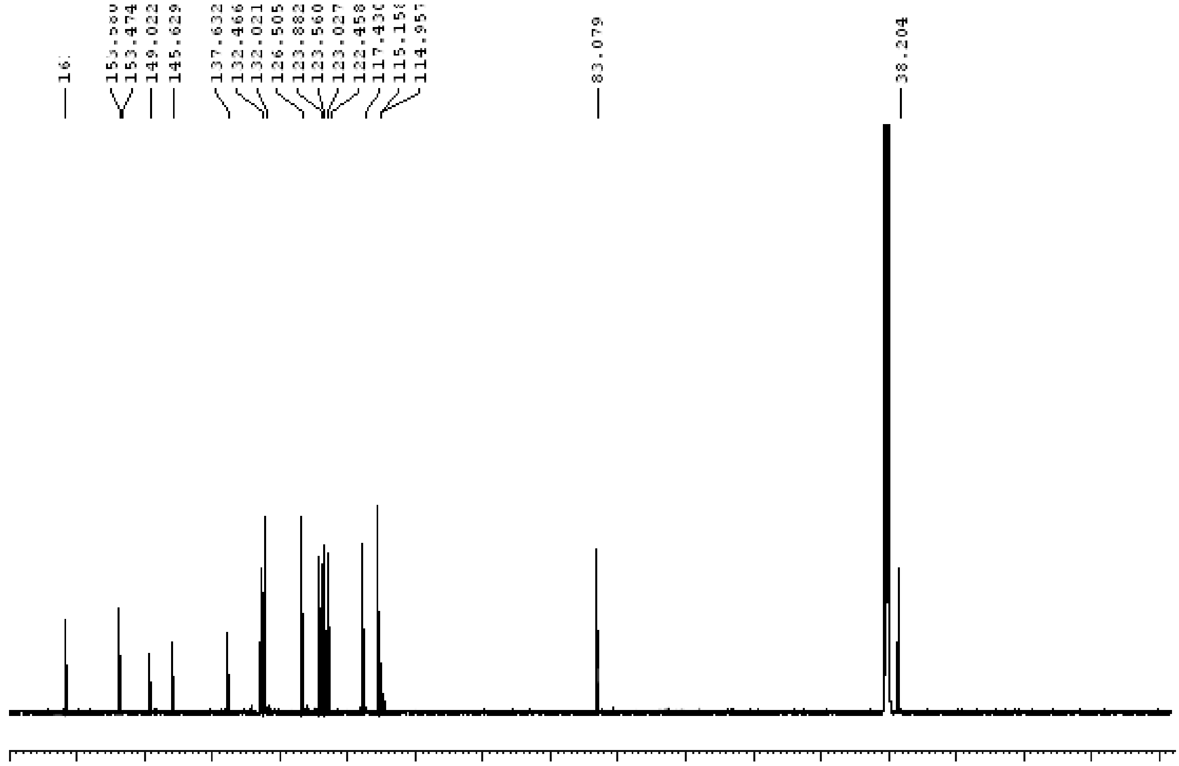

^{1}H-NMR ((CD$_3$)$_2$SO, 500 MHz) of compound 9i.

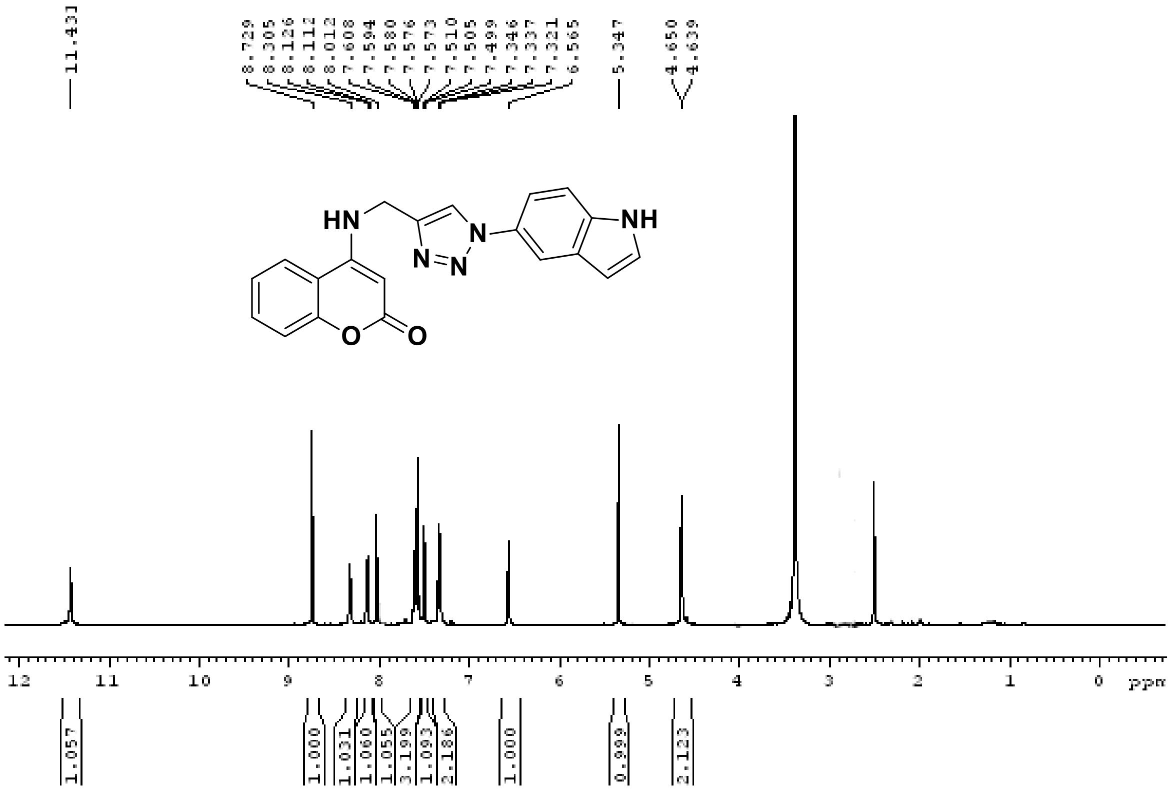

^{13}C-NMR ((CD$_3$)$_2$SO, 125 MHz) of compound 9i.

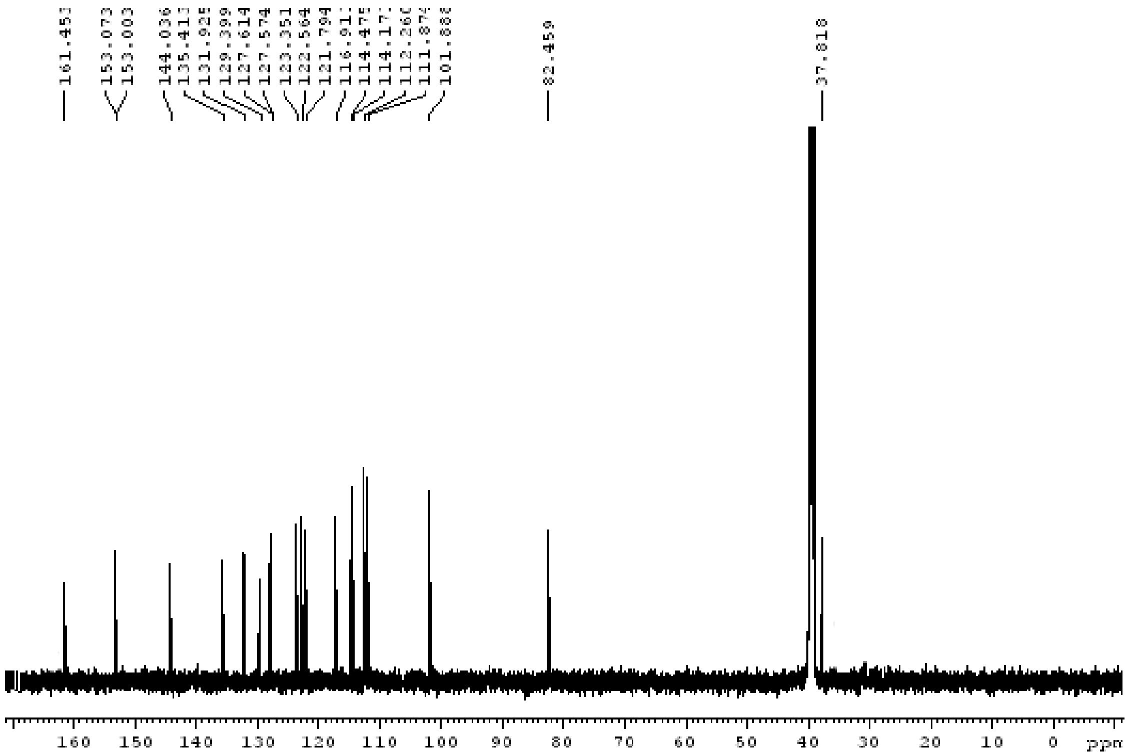

^{1}H-NMR (($(CD_3)_2SO$, 500 MHz) of compound 9j.

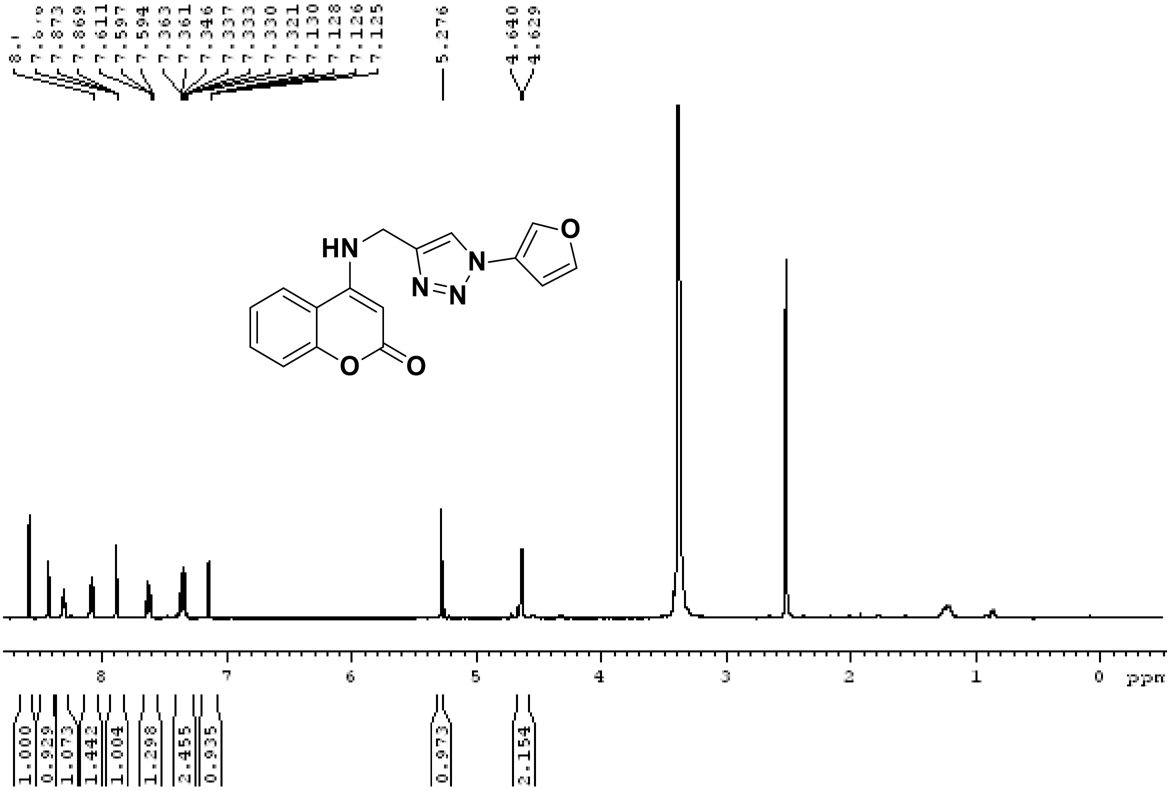

^{13}C-NMR (($(CD_3)_2SO$, 125 MHz) of compound 9j.

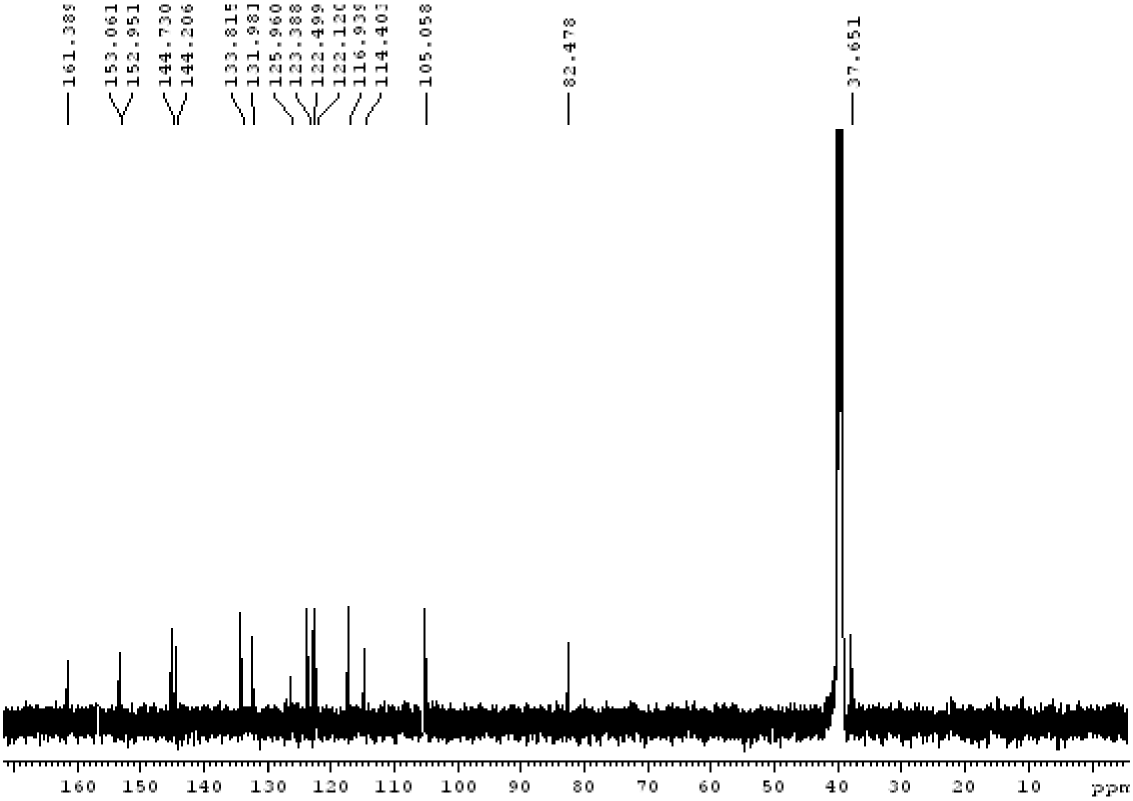

^{1}H-NMR ((CD_3)$_2$SO, 500 MHz) of compound 9k.

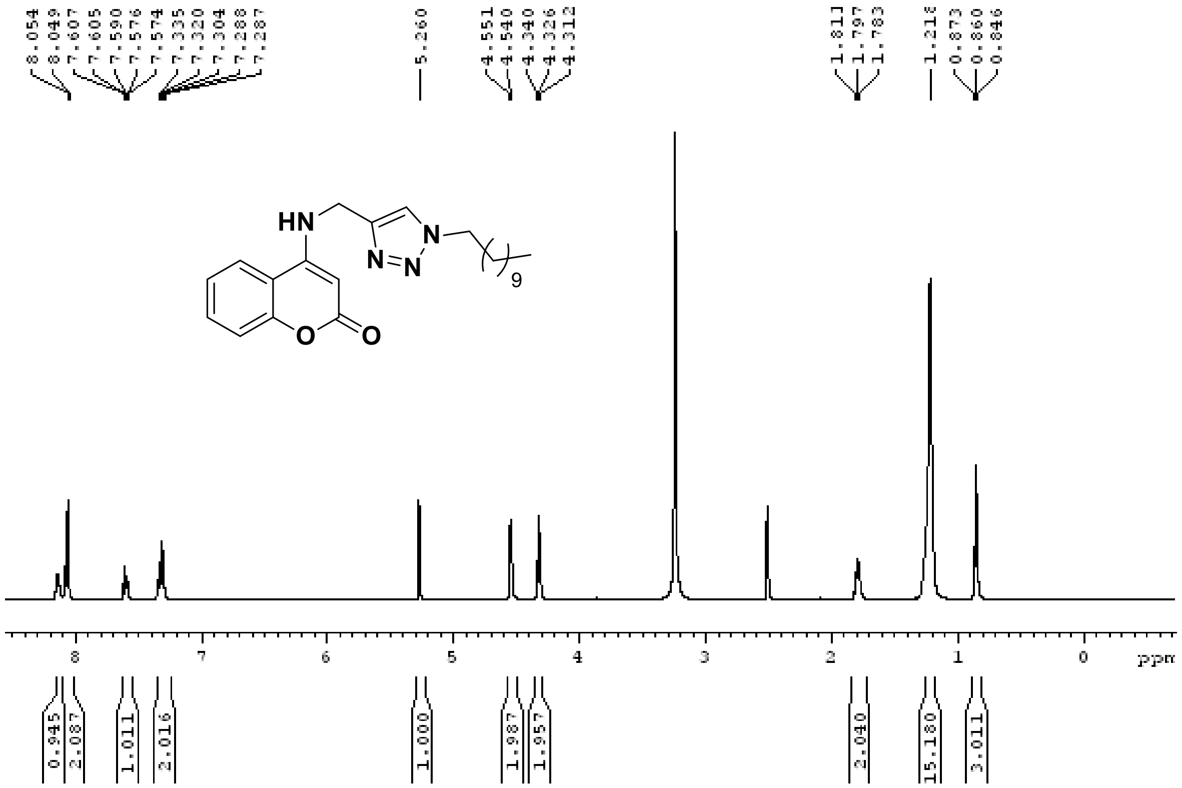

^{13}C-NMR ((CD_3)$_2$SO, 125 MHz) of compound 9k.

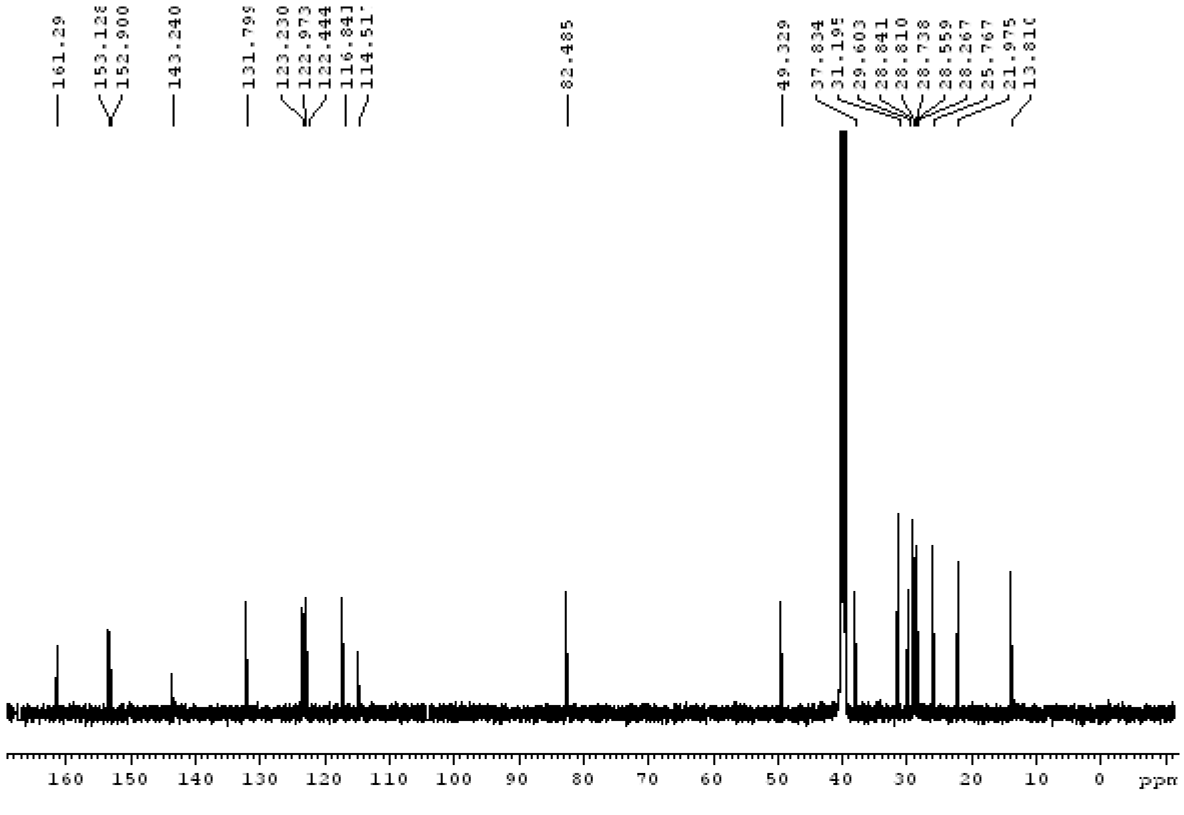

^{1}H-NMR ((CD$_3$)$_2$SO, 500 MHz) of compound 9l.

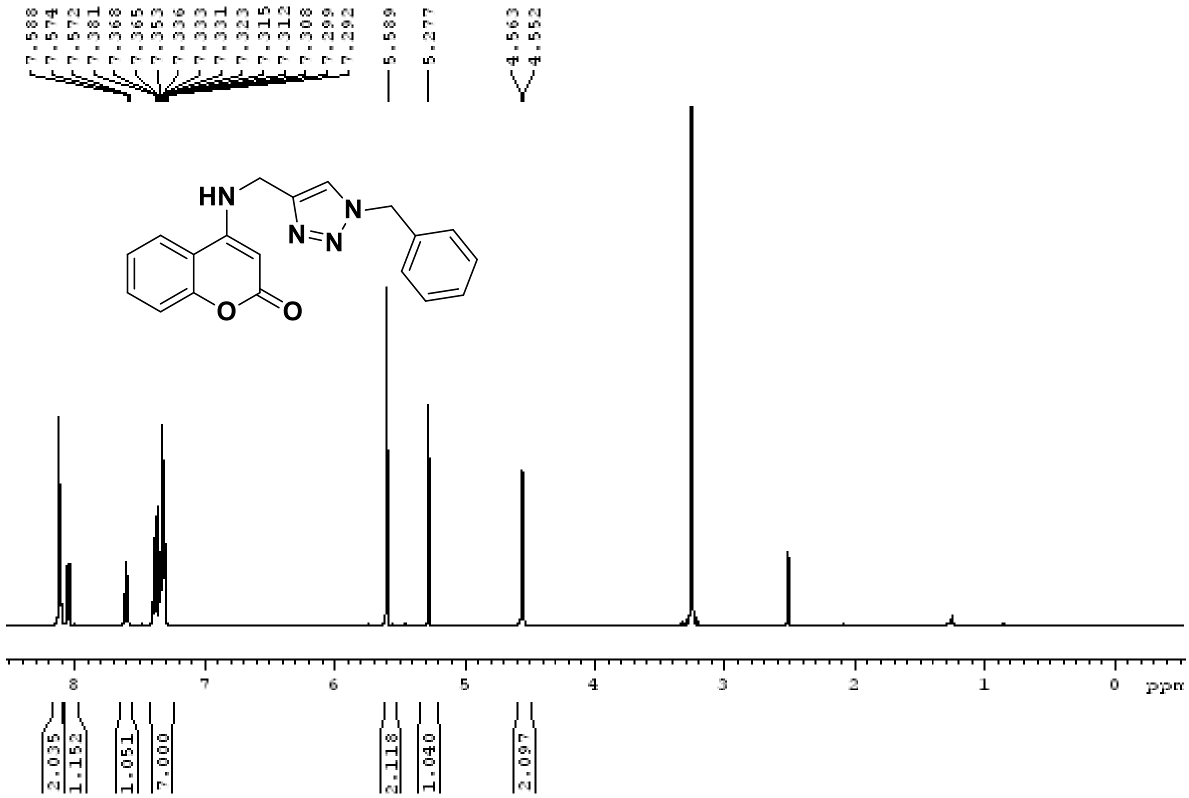

^{13}C-NMR ((CD$_3$)$_2$SO, 125 MHz) of compound 9l.

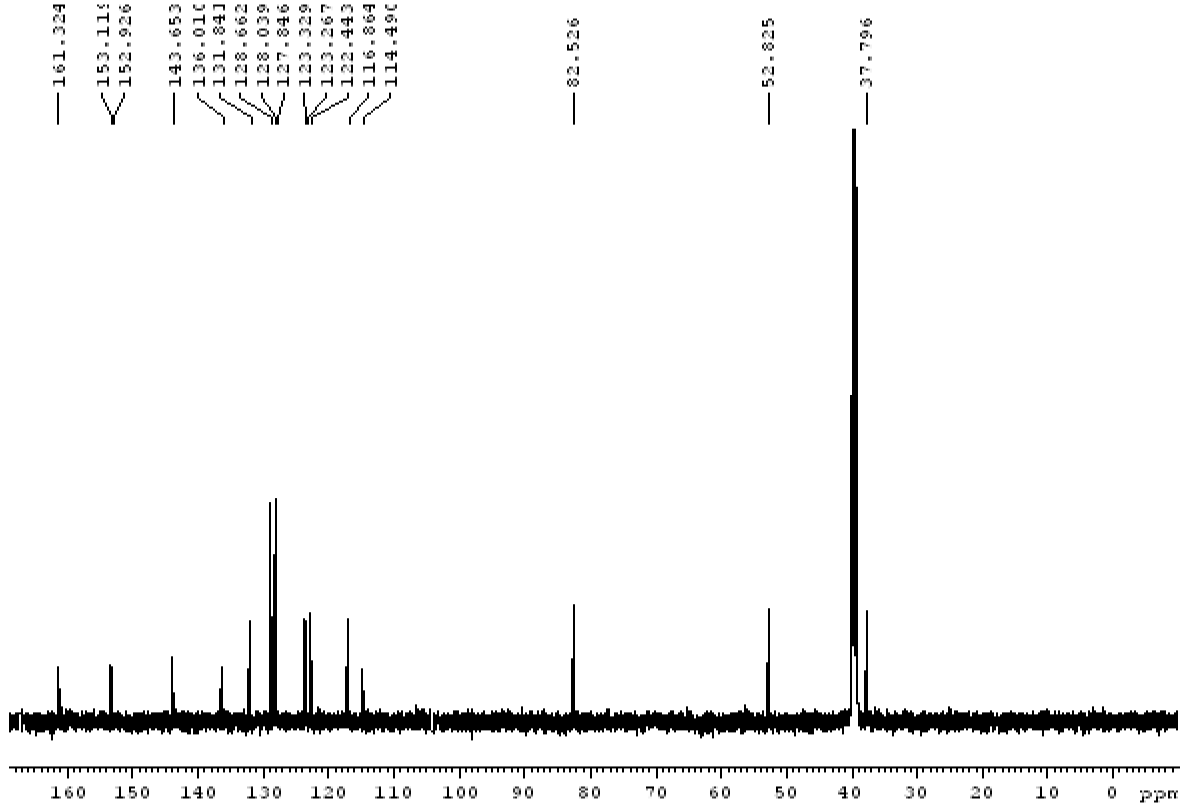

^{1}H-NMR ((CD3)2SO, 500 MHz) of compound 9m.

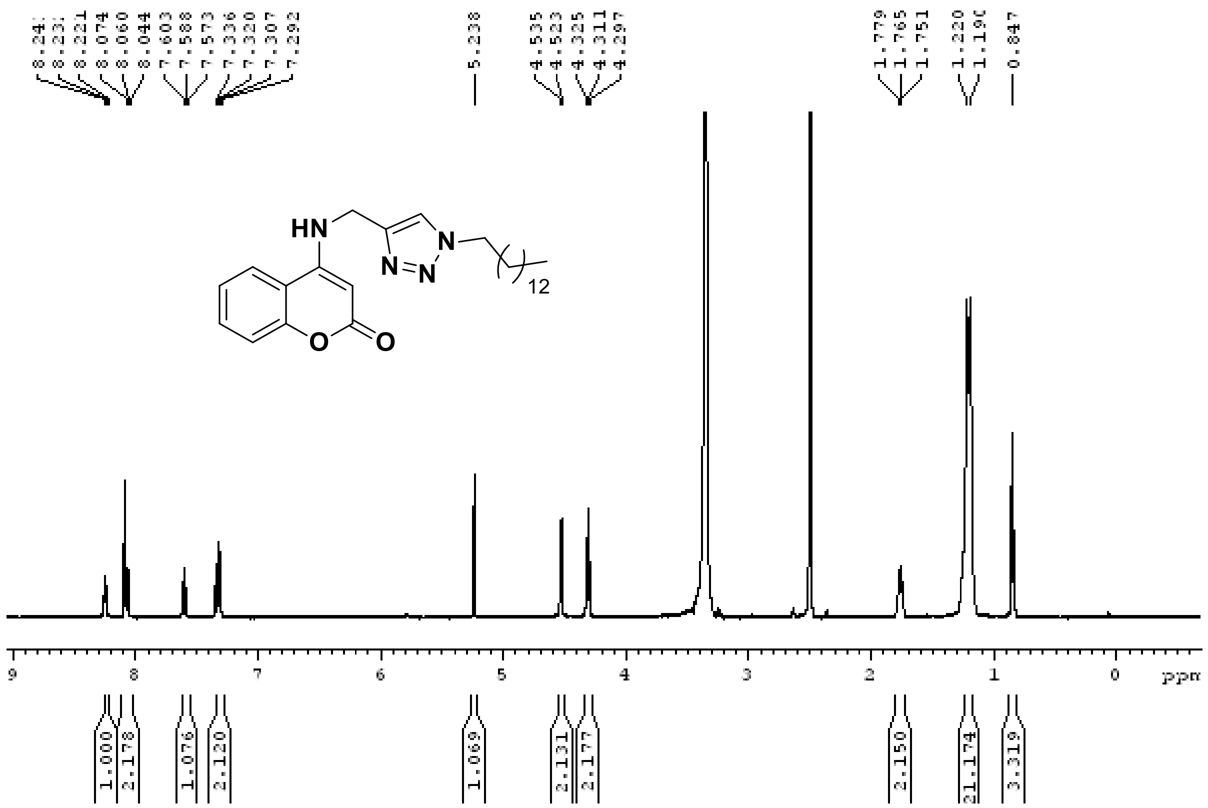

^{13}C-NMR ((CD3)2SO, 150 MHz) of compound 9m.

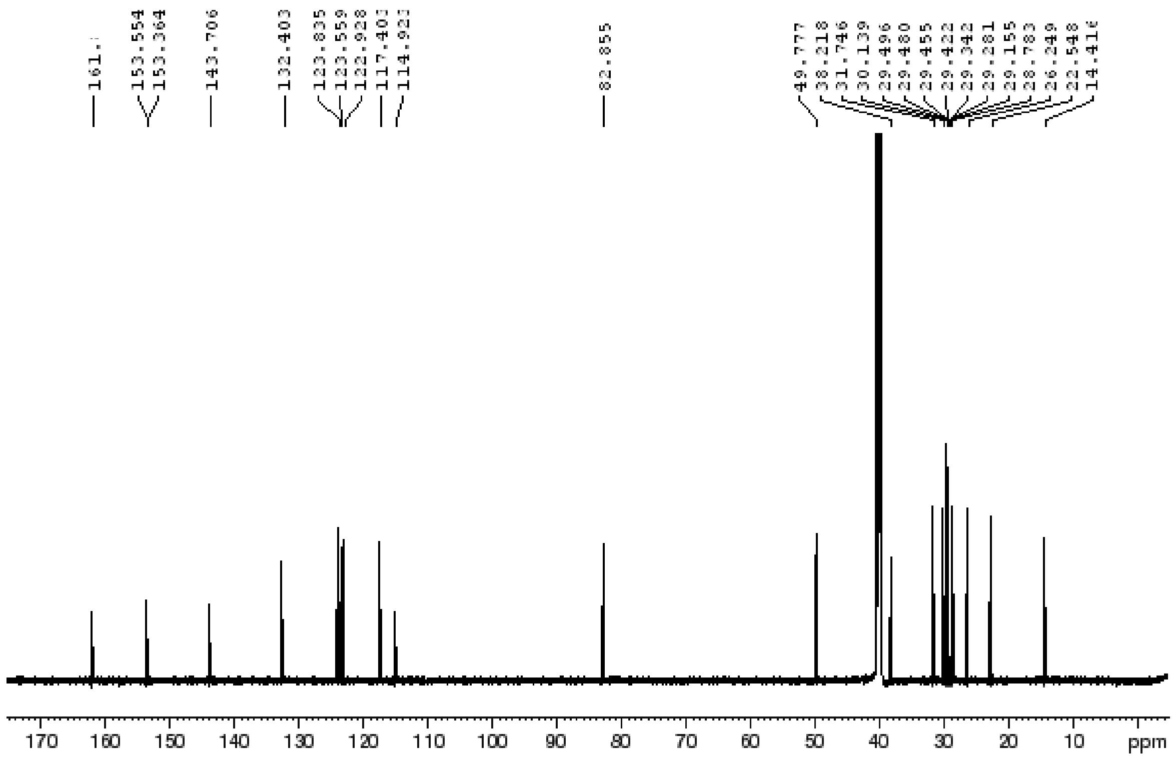

Supplement: Supplementary file 1 [file molecules-23-00199-s001.pdf]
